# Do psychosocial factors mediate the appearance of musculoskeletal symptoms? Evidence of an empirical study about the role of mental workload in computer workers

**DOI:** 10.1371/journal.pone.0252179

**Published:** 2021-06-17

**Authors:** María Soria-Oliver, Jorge S. López, Fermín Torrano, Guillermo García-González

**Affiliations:** 1 Facultad de Ciencias de la Salud, Universidad Pública de Navarra, Pamplona, Navarra, Spain; 2 IdiSNA-Instituto de Investigaciones Sanitarias de Navarra, Pamplona, Spain; 3 UNIR-Universidad Internacional de la Rioja, Logroño, La Rioja, Spain; Jonkoping University, SWEDEN

## Abstract

The emergence of musculoskeletal symptoms (MSSs) in computer workers is a relevant occupational health problem. This study tests a multilevel model of analysis of risk factors in the appearance of musculoskeletal pain and discomfort in computer workers that integrates indicators from different areas: temporal usage patterns, ergonomic factors, psychosocial factors, and individual variables, specifically testing the possible mediating role of the mental workload. A cross-sectional study was performed through online registration with a non-probabilistic sample of 1198 workers from Spanish organizations. The results show that mental workload has a higher association than the rest of the factors with the onset of pain and discomfort in various body areas: neck in men, neck, shoulders and upper back in women. They also support the mediation role of mental workload in the relationship between usage patterns and the appearance of musculoskeletal symptoms. The use of multilevel theoretical models that adequately consider the complexity of the relationships between the different risk factors is necessary for a better understanding and intervention on MSSs in computer workers.

## 1. Introduction

Advances in information and communication technologies (ICT) have made the computer an essential work element across all activity sectors and business-size classes [1, 2]. Among the different consequences that have been associated with this increasing computer usage is the appearance of musculoskeletal symptoms (MSSs) and musculoskeletal disorders (MSDs). Some reviews in the first decade of the 21st century already highlighted the greater incidence of de MSDs in computer workers and defended that computer use was a risk factor for the appearance of MSDs and MSs [3–5]. However, more recent reviews are cautious about establishing direct causal links between computer usage and the emergence of MSS and MSDs, noting the limited level of evidence and the inconsistency of the findings [6–8]. At present, there is a clear consensus that the occurrence of MSSs and MSDs in computer workers should be conceptualized as a multi-causal process that includes factors from different areas [9]. Among the most important features associated with MSSs as a whole in computer workers are the following: hours spent working at a computer and sustained awkward postures [10–16]; physical environment and design features of office workspace and buildings [17–21]; work organization and psychosocial factors (workload, job strain, job characteristics, high job demands, lack of job autonomy, limited support from coworkers or supervisors, among others) [14, 20–31]; and individual factors like age, gender and performance of physical activity outside of work [8, 13, 14, 21].

As mentioned, numerous studies have shown the association between organizational and psychological factors and the onset of MSSs in computer workers. However, few works have theoretically or empirically proposed the interaction between the physical and environmental dimensions of computer usage, on the one hand, and the organizational and psychological dimensions, on the other, in the explanation of the appearance of MSSs. In this sense, we note the study of Wahlström [31] in which he explored the interaction between job strain, perceived muscular tension, and physical exposure in Video Display Terminal users and concluded that the combination of high job strain and high perceived muscular tension was associated with a higher risk of developing neck pain. Also notable is the conceptual effort of that author [20], in which he proposed a model of overall relationships between the mental, organizational, and physical dimensions in the determination of MSDs, based on the ecological model of musculoskeletal disorders of Sauter and Swanson [32]. However, the multiple relationships postulated in this model have been very little explored in the literature. The study of Lapointe et al. [27], should also be mentioned, which analyzed the interaction between postural risk factors and job strain on the incidence of self-reported MSSs among users of video display terminals. These authors concluded that the simultaneous presence of postural risk factors and job strain seems to increase the pathogenic effect of each exposure on the incidence of musculoskeletal symptoms. More recently, Robertson et al. [14] analyzed the moderating role of supervisory relations and co-worker support in the relationship between computer work, environmental design, and musculoskeletal discomfort. They found that supervisor relations partially moderated the relationship between workspace design satisfaction and musculoskeletal discomfort.

The mentioned works support the interest of analyzing at a higher level of complexity the interactions between environmental and physical factors, on the one hand, and organizational and psychosocial factors, on the other, to understand the genesis of MSSs in workers exposed to ICT use. The formulation of empirically-based models that go beyond additive relationships and consider interaction and mediation/moderation relationships within factors may help to enhance the predictive power of physical/environmental, ergonomic, and psychosocial/organizational factors in the appearance of MSSs. Such models would reveal influences that may have been masked in past literature, thus helping to design adequate intervention and prevention strategies.

The analysis of the relationships of interaction, moderation, and mediation in global relationship structures among variables has recently undergone significant development in the behavioral sciences. This development has increased our ability to address the complexity of human behaviors and their manifestations. Since the pioneering theoretical work of Baron and Kenny [33] on the analysis of the effects of mediation and moderation, several authors have developed conceptual and analytical proposals that have allowed an adequate approximation and interpretation of this type of relationships [34, 35]. These advances can be used to more accurately analyze the relationship structures between the different factors that determine the occurrence of MSs in computer workers.

The objective of this work is to empirically test the potential mediator role of a specific psychosocial/organizational factor, Mental Workload (MWL), in the relationships between computer use patterns, on the one hand, and the appearance of self-reported MSSs, on the other hand, also considering the joint influence of ergonomic and individual factors. For this purpose, we will use a conceptual framework that integrates indicators related to different areas and we will apply recent advances in mediation process analysis strategies. This conceptual framework and the relationship hypotheses it comprises are presented in the next section.

## 2. Conceptual framework and hypotheses

**Fig 1:**
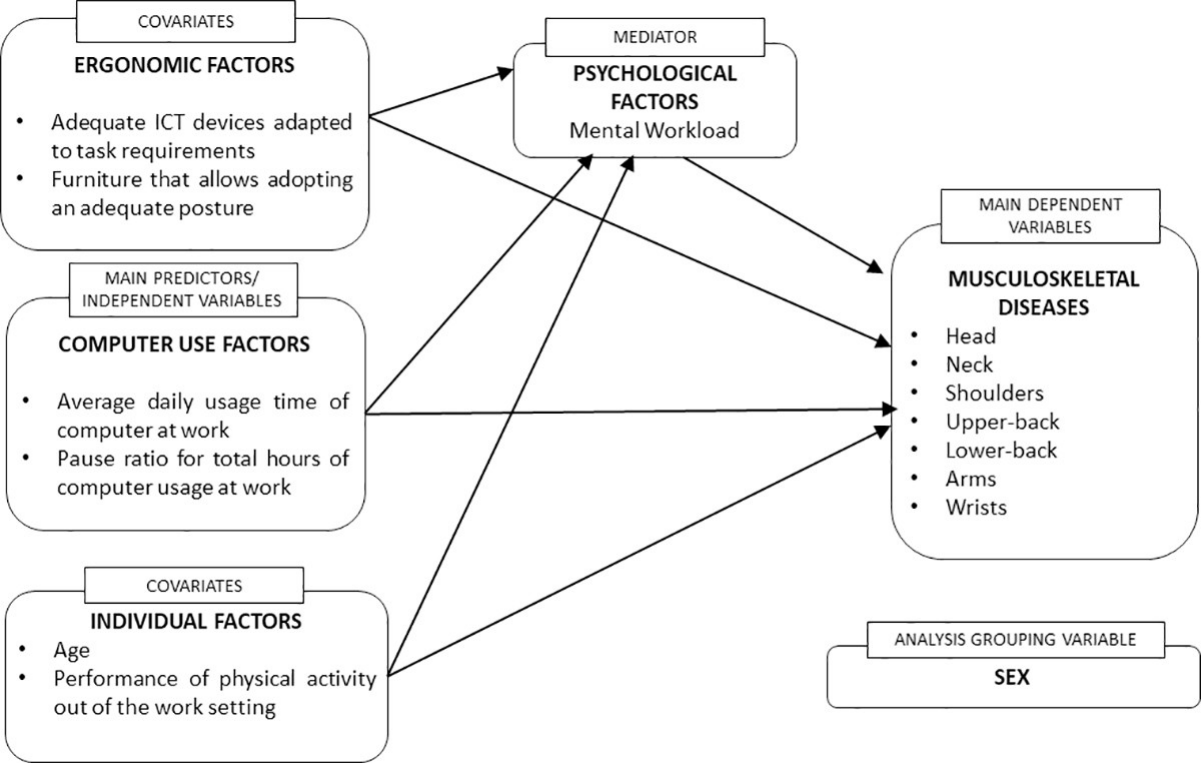
Conceptual framework and structure of relations among indicators.

When designing the relationship structure proposed in this study, we drew on the evidence in the literature of the most relevant factors in the emergence of MSSs in computer workers, reflected globally in some of the most recent reviews [6–9, 13, 36]. Due to parsimony criteria and to be able to integrate the different variables into a global model, the conceptual framework has been reduced to a certain number of relevant indicators in the following areas: usage factors, ergonomic factors, psychological factors, and individual factors. Our interest, in this case, is not to carry out a thorough exploration of all possible indicators, but to perform a first approximation to the study of the inter-relationships between the different areas. The conceptual framework is reflected in Fig 1.

The occurrence of MSSs in different body areas is considered as a dependent variable/essential criterion. To operationalize it, we selected the appearance of MSSs in those areas of the body that have shown greater impairment due to computer usage: head, neck, shoulders, upper and lower back, arms and wrists [7, 8, 12, 13, 19].

Indicators referring to computer usage at work are conceptualized as the main independent/predictor variables of MSSs. We selected indicators related to the use of desktop computers and laptop computers because their usage has been more clearly related to MSSs than other moving elements such as tablets or smartphones [15, 37, 38]. Although laptop and desktop use may have different incidence on MSSs, we decided to use a single measure of average daily time of computer usage to obtain a global indicator of physical strain suffered by workers due to computer usage. This decision was made taking as reference similar measures performed in relevant studies [15, 31, 39]. Two indicators were selected to operationalize global computer usage: usage time, which has emerged in the most recent reviews as one of the strongest factors associated with MSSs in computer workers [7, 10–16]; and frequency of pauses, which was operationalized as the ratio of pauses/hour, also based on its relevance in classic and recent literature about the effect of ergonomic interventions on MSSs [17, 23, 34, 40].

Psychological factors are considered in this conceptual framework as mediators of the relationship between computer usage and the occurrence of MSSs, as suggested in recent models [20, 32]. In this sense, according to the mentioned works, we assume that sustained computer usage would increase the appearance of MSSs due in part to an increase in psychological overload. We assume, in any case, that there will also be a direct effect of computer usage on the appearance of MSSs, as biomechanical studies seem to show [41, 42]. Among the diversity of concepts and indicators used in the literature, we have chosen a construct of great tradition in the ergonomic field that very adequately reflects the influence of different factors on the worker’s psychological dimension: Mental Workload (MWL) [43, 44]. MWL allows dimensioning the extent to which occupational activity matches or exceeds the worker’s resources. Current perspectives conceptualize MWL as a consequence of the relation between task demands and the subject’s skills. In this sense, the MWL construct integrates different aspects: time pressure, the processing resources required by the task, and related emotional aspects, such as stress, perception of fatigue, and perceived consequences for health [45, 46]. This concept, therefore, reflects the essential aspects that have been addressed in the literature under psychological and organizational dimensions, such as job strain, job stress, or high job demands. The "Escala Subjetiva de Carga Mental de Trabajo" (ESCAM, [Subjective Mental Workload Scale]) was used [47] to assess the MWL from the workers’ perception. This general scale presents good psychometric properties and allows assessing the mental load in different jobs of different work sectors, both in the services sector and in the industrial sector. Following the most recent trends in the field of mediation relationship analysis [34], we will analyze the total effect that computer usage has on the occurrence of MSSs and the extent to which part of that effect is divided between its direct and indirect action through the increase in MWL.

Ergonomic factors are included in the conceptual framework as covariates; that is, factors whose contribution must be considered as an additional source of variation to adequately interpret the relationship between predictor variables and the main criterion variables. We selected two indicators that have shown a potential effect to reduce the appearance of MSSs in computer workers: the existence of adequate ICT devices adapted to task requirements and the existence of furniture that allows adopting an adequate posture to perform the task [8, 13, 17, 19, 37].

Individual factors have been integrated in the proposed model as covariates to control for their influence on MSSs and thus make a more accurate estimate of the influence of the main predictor variables. In this sense, although the evidence of the relationships between age an MSSs in computer users is limited mainly to cross-sectional studies, age was included due to the relevant number of works that suggest its role in the increase of MSSs incidence [7, 16, 48, 49]. The performance of intense physical activity outside the work environment was also included as covariate because, according to literature, it could represent an additional risk factor for the appearance of MSSs in computer workers [8, 13, 14]. Concerning sex, diverse studies have shown the existence of distinct patterns of relationship between the indicated factors in men and women [13, 27, 31]. Therefore, taking as reference the strategy used by other authors [27, 31], their effect will be analyzed stratified by sex.

**Fig 2:**
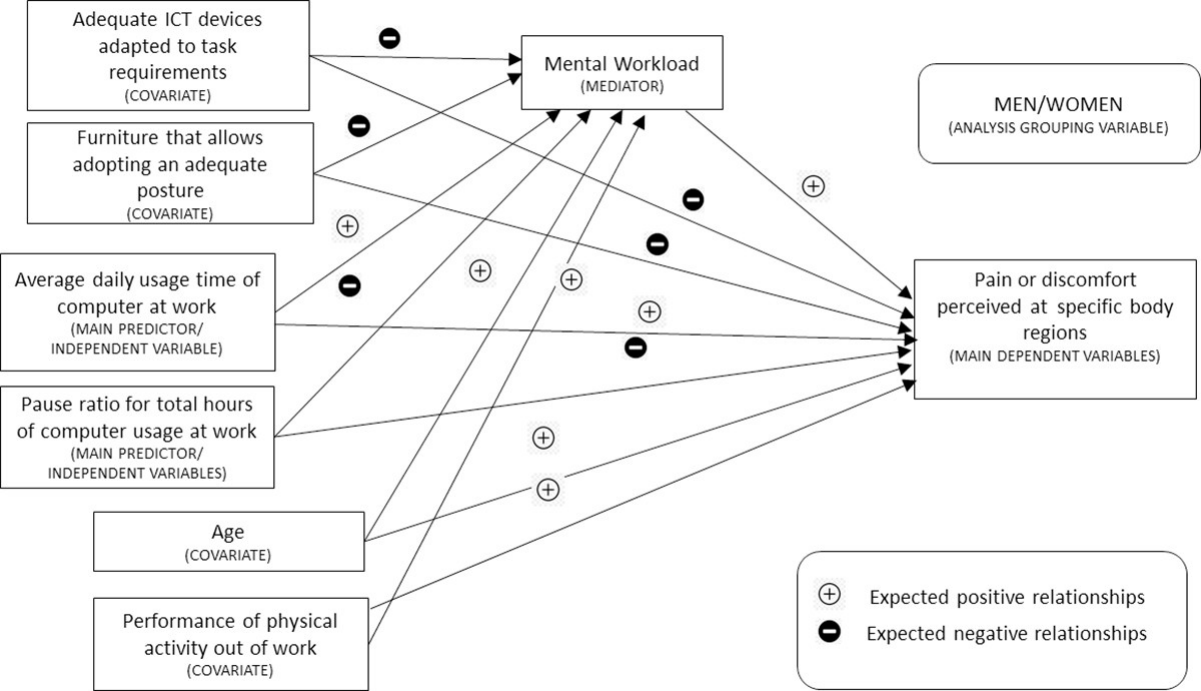
Hypothesis of relationships among indicators.

The relationship hypotheses among indicators are specified in greater detail in Fig 2. Concerning the direct relationships between the emergence of MSSs and the different indicators, the following relationships are expected: a positive relationship with MWL, a negative relationship with the adoption of ergonomic measures, a positive relationship with the total time of desktop and laptop computer usage, a negative relationship with pause ratio for total hours of computer work, a positive relationship with age, and a positive relationship with the performance of intense physical activity outside of work. Regarding relations with MWL, the selected indicators are expected to maintain the following relationships: a negative relationship with the adoption of ergonomic measures, a positive relationship with the total time of desktop and laptop computer usage, a negative relationship with pause ratio for total hours of computer work, a positive relationship with age, and a positive relationship with the performance of intense physical activity outside of work.

## 3. Materials and methods

### 3.1. Subjects

A non-probabilistic sample of 1198 subjects (527 men and 671 women) of Spanish organizations belonging to different productive sectors were recruited to complete an on-line instrument. For sample selection, the research team contacted 512 prevention managers of different companies at a national level who had accepted to collaborate in previous studies. In this case, the research team sent to the involved managers an email message in which study objectives were presented and a link to the on-line instrument was included, requesting their collaboration. Prevention managers forwarded the email to workers, ensuring the anonymity of the answers. A total of 1198 workers sent the completed questionnaire. Table 1 presents the distribution of the sample according to different activity sectors. It also includes the percentage of workers included in each sector in Spain, to provide a global view of sample concordance with the Spanish sectors of activity. However, the sample selection procedure does not allow making direct inferences about the incidence of MSSs in Spanish organizations. Table 2 presents additional sociodemographic characteristics of the sample.

**Table 1:** Sector of activity of the sample and of the total Spanish workers.

		Sample				Spain [50]	
		Men		Women		Men	Women
		n	Column %	n	Column %	Column%	Column%
Sector of activity	Agriculture	14	2.7	21	3.1	5.7	2.0
	Construction	66	12.5	56	8.3	10.8	1.3
	Industry	120	22.8	96	14.3	18.9	8.1
	Services	327	62.0	498	74.2	64.6	88.6

**Table 2:** Main socio-demographic characteristics of the sample.

		Men		Women	
		n	%Column	N	%Column
Type of job	Management, administration, reception	272	51.6	487	72.6
	Production, operator	42	8.0	11	1.6
	Commercial department	14	2.7	11	1.6
	Services and activities	46	8.7	9	1.3
	Others	153	29.0	153	22.8
Job tenure	Less than 1 year	17	3.2	28	4.2
	1–5 years	133	25.2	222	33.1
	6–10 years	57	10.8	97	14.5
	11–15 years	86	16.3	113	16.8
	>15 years	234	44.4	211	31.4
Age	18–25	9	1.9	10	1.7
	26–35	51	11.2	102	17.1
	36–45	171	37.7	154	42.6
	46–55	169	37.2	186	31.2
	56–65	52	11.5	44	7.4
	> 65	2	.4	0	0

### 3.2. Instrument

An on-line questionnaire was used, including the following measures presented in Table 3.

**Table 3:** Variables and indicators.

*Sociodemographic data*:	Sex, age
*Job data*:	Sector of activity, type of job, job tenure.
*ICT usage in the work activity*:	Average daily usage time (hours) of desktop for work-related activities.
	Average daily usage time (hours) of laptop for work-related activities.
	Average number of daily pauses during ICT usage (informed by subjects)
*Musculoskeletal Symptoms (MSSs)*	Nordic Musculoskeletal Questionnaire (NMQ) [51]. Check-list of 9 items referring to pain or discomfort perceived by workers in different body parts suffered for seven days following the use of ICT. Response scale ranging from 1 (no pain or discomfort) to 10 (maximum intensity of pain or discomfort).
*Mental Workload*	“Escala Subjetiva de Carga Mental de Trabajo”(ESCAM [Subjective Mental Workload Scale]) [47]. This estimates the mental workload construct through 5 dimensions presented in the 20 items of the questionnaire: Cognitive Demands and Task Complexity (Items 1, 2, 3, 4, and 8); Task Characteristics (Items 6, 7, 10, and 13); Temporal Organization (Items 18, 19, and 20), Work Rhythm (Items 11, 12, and 14) and Health Consequences (Items 9, 15, 16, and 17).
*Ergonomic factors*	Furniture that allows adopting an adequate posture to perform the task (Yes/no).
	Appropriate devices or equipment for the task being performed (Yes/no).
*Performance of intense physical activity outside of the work setting*:	An item that shows the extent to which workers practice hobbies or intensive sport activities, play musical instruments, or practice activities with instruments that produce vibrations (lawnmower, motor saw, Do-it-yourself tools, etc.) in their free time. Scale from 0 (Never) to 10 (High frequency/intensity) points.

### 3.3. Procedure

The prevention services managers who accepted to participate in the study sent an email to the workers, presenting the main objectives of the study, requesting their collaboration, and ensuring the anonymity of their responses. After requesting and obtaining written informed consent, the questionnaire was sent to participants, who completed it anonymously. The questionnaire did not include any identification or personal or company data. The workers completed the questionnaire during April and May of 2018. The responses were collected directly in the database linked to the questionnaire.

### 3.4. Ethical considerations

Study procedures were designed according to the principles expressed in the Declaration of Helsinki, were also elaborated in consensus with those in charge of the Spanish National Institute of Safety and Health at Work and obtained previous ethical approval by the Committee of the involved academic institution.

### 3.5. Data analysis

The following analysis will be performed: 1) Application of factor-reduction strategies to ESCAM measurements in order to generate a single and reliable score that can be included in the following phases of analysis; 2) Descriptive analysis of main variables, including calculation of centrality and deviation indexes and normality test; 3) Exploratory analysis of bivariate relationships between main predictors/independent variable and mediator, on the one hand, and dependent variable, on the other hand, to determine whether the suggested linear association patterns are supported by the data; 4) Correlation analysis between all variables included in the conceptual model (main predictors/independent variables, mediator, covariates, and dependent variable); according to significance levels of this correlation matrix, the MSSs indicators that have relevant relationships with the indicators of computer usage and MWL will be selected in each of the groups (men and women) to perform the following phases of analysis; 5) Structural Equation Modeling (SEM) to demonstrate the overall relationships between variables of the proposed conceptual model; 6) Mediation analysis with estimation of total, direct and indirect effects in order to quantify the contribution of each predictor/independent variable to the dependent variable and the potential mediation effect of MWL on MSSs. The analyses will be carried out with the following software: IBM®-SPSS Statistics® V26.0 (basic analysis), IBM®-AMOS® 24.0 (structural equation modeling), and the PROCESS macro for SPPS (mediation analysis) developed by Hayes and Preacher [52].

## 4. Results

### 4.1. Factor reduction of MWL measure (ESCAM scale)

For reasons of parsimony, in order to generate a limited number of mediation models, it was necessary to reduce the MWL construct measurement to a single score. To ensure adequate reliability of the MWL measure, the set of scores of the ESCAM dimensions (Cognitive Demands and Task Complexity, Task Characteristics, Temporal Organization, Work Rhythm and Health Consequences) was subject to a principal component factor analysis (PCFA). PCFA allows grouping the maximum percentage of variance observed in a set of observed variables into a reduced number of new variables [53]. PCFA guarantees a greater contribution to the total score of the dimensions that are more consistent with the construct, thus offering a more reliable measure than their simple addition [53]. PCFA was performed in this case to generate a single factor, in order to retain the factorial score obtained as an MWL measure for further analysis. The single factor captured 44% of the total variance contained in the ESCAM dimensions.

### 4.2. Distribution properties and descriptive results

Shapiro-Wilk test was performed to examine the distribution of the selected numerical variables. The analysis showed that, with the exception of MWL measure (Shapiro-Wilks statistic = .999; p = .880), the selected study variables do not fit a normal distribution.

In Table 4, the descriptive results of the main indicators for each of the groups (men and women) are shown. We added the results of Student’s *t* mean comparison to show the significance of the differences between scores. As can be seen, in the sample used, the men reported less computer time usage, a greater ratio of pauses per hour, and a lower mean age, although neither the presence of ergonomic factors nor the mental load showed sex differences. In contrast, the incidence of MSSs was, in all cases, higher in women.

**Table 4:** Descriptive statistics of the main variables.

		Group			
		Men		Women	
	Student’s t	Mean	Standard Deviation	Mean	Standard Deviation
Average daily time of computer usage	7.041 (p < 001)	4.78	2.67	5.79	2.18
Pause ratio for total hours	6.089 (p < .001)	.79	.91	.50	.57
MWL Factor	.048 (p = 962)	-.01	.95	.00	1.04
Adequate ICT devices adapted to top task requirements	.913 (p = .362)	.63	.48	.60	.49
Furniture that allows adopting an adequate posture	1,099 (p = .272)	.66	.48	.69	.46
Performance of intense physical activity outside of work	10.075 (p < .001)	4.30	3.05	2.67	2.54
Age	4.167 (p < .001)	45.0	8.79	42.93	8.39
MSSs Wrists	5.423 (p < .001)	2.27	1.94	2.96	2.48
MSSs Shoulders	8.353 (p < .001)	3.20	2.45	4.55	3.02
MSSs Neck	8.044 (p < .001)	4.48	2.76	5.81	2.90
MSSs Head	6.780 (p < .001)	3.53	2.56	4.60	2.90
MSSs Upper back	8.098 (p < .001)	4.22	2.83	5.61	3.05
MSSs Lower back	3.691 (p < .001)	3.96	2.82	4.58	2.92

Abbreviations: MWL: Mental Work Load; MSSs: Musculoskeletal Symptoms.

### 4.3. Exploratory analysis of bivariate relationships and correlation matrix

We performed an exploratory analysis of bivariate relationships between the variables included in our conceptual framework, in order to test the assumption of linearity. Partial regression analysis between the daily time of computer usage and MWL, by one side, and MSSs indicators, by the other side, were generated. The resulting partial regression plots and standardized residual distribution are included in S1 File. Partial regression plots are compatible with a linear regression pattern of relationships between MSSs and the considered variables in all cases. Additionally, standardized residual distributions follow a normal distribution trend, especially for those partial regression models that involve MSSs in the head, shoulders, and upper and lower back. The distribution pattern shows some asymmetry in the case of MSSs in the wrists.

Kendall’s tau-b correlation coefficient was applied to explore bivariate association between the complete set of variables. This coefficient is adequate for data sets that include variables with a non-normal distribution and dichotomic indicators [54], which is the case of the data presented. Kendall’s rank correlation measures the strength of the monotonic association between the selected pair of variables and can range between -1 (perfect negative relationship) and 1 (perfect positive relationship). Tables 5 and 6 present the resulting correlation matrix of the selected variables in men’s and women’s groups and include the significance level based on a two-tailed test according to an IBM®-SPSS® algorithm.

**Table 5:** Correlation matrix. Men’s group.

		1	2	3	4	5	6	7	8	9	10	11	12	13
1. Average time of computer use		1.000												
2. Pause ratio for total hours	Tau-b correlation	-.282	1.000											
	*p (two-tailed)*	.*000*												
3. MWL Factor	Tau-b correlation	.127	-.077	1.000										
	*p (two-tailed)*	.*000*	.*016*											
4. Adequate ICT devices	Tau-b correlation	.034	-.019	-.088	1.000									
	*p (two-tailed)*	.370	.626	.016										
5. Furniture that allows adequate posture	Tau-b correlation	.148	-.030	-.054	.446	1.000								
	*p (two-tailed)*	.*000*	.*432*	.*139*	.*000*									
6. Physical activity outside of work	Tau-b correlation	-.026	-.017	.038	.056	.002	1.000							
	*p (two-tailed)*	.*417*	.*602*	.*229*	.*137*	.*951*								
7. Age	Tau-b correlation	.007	.040	-.065	-.062	.010	-.061	1.000						
	*p (two-tailed)*	.*841*	.*232*	.*040*	.*104*	.*786*	.*066*							
8. MSSs Wrists	Tau-b correlation	-.036	.064	.202	-.155	-.079	.083	-.014	1.000					
	*p (two-tailed)*	.*287*	.*067*	.*000*	.*000*	.*046*	.*016*	.*687*						
9. MSSs Shoulders	Tau-b correlation	.029	.072	.276	-.114	-.066	.063	.001	.517	1.000				
	*p (two-tailed)*	.*378*	.*033*	.*000*	.*003*	.*087*	.*058*	.*977*	.*000*					
10. MSSs Neck	Tau-b correlation	.116	-.043	.312	-.103	-.054	.077	-.042	.375	.578	1,000			
	*p (two-tailed)*	.*000*	.*192*	.*000*	.*006*	.*150*	.*018*	.*200*	.*000*	.*000*				
11. MSSs Head	Tau-b correlation	.067	-.008	.341	-.068	-.095	.046	-.092	.357	.427	.486	1.000		
	*p (two-tailed)*	.*127*	.*800*	.*000*	.*075*	.*013*	.*166*	.*006*	.*000*	.*000*	.*000*			
12. MSSs Upper back	Tau-b correlation	.048	-.004	.296	-.085	-.065	.031	-.019	.396	.522	.623	.455	1.000	
	*p (two-tailed)*	.*137*	.*900*	.*000*	.*025*	.*083*	.*337*	.*572*	.*000*	.*000*	.*000*	.*000*		
13. MSSs Lower-back	Tau-b correlation	-.028	.039	.264	-.100	-.080	.124	-.051	.306	.389	.443	.432	.495	1.000
	*p (two-tailed)*	.*388*	.*238*	.*000*	.*008*	.*034*	.*000*	.*124*	.*000*	.*000*	.*000*	.*000*	.000	

Abbreviations: ICT: Information and Communication Technologies; MWL: Mental Work Load; MSSs Musculoskeletal Symptoms

Note: Correlation indexes with a significance level of *p* < .05 have been highlighted.

**Table 6:** Correlation matrix. Women’s group.

		1	2	3	4	5	6	7	8	9	10	11	12	13
1. Average time of computer use		1.000												
											
2. Pause ratio for total hours	Tau-b correlation	-.275	1.000											
	*P (two-tailed)*	.*000*		1.000										
3. MWL Factor	Tau-b correlation	.162	-.162											
	*P (two-tailed)*	.*000*	.*000*											
4. Adequate ICT devices	Tau-b correlation	.001	.090	-.147	1.000									
	*P (two-tailed)*	.*976*	.*006*	.*000*										
5. Furniture that allows adequate posture	Tau-b correlation	.056	.079	-.102	.440	1.000								
	*P (two-tailed)*	.*100*	.*016*	.*002*	.*000*									
6. Physical activity outside of work	Tau-b correlation	-.033	.048	.014	.072	.024	1.000							
	*P (two-tailed)*	.*288*	.*111*	.*632*	.*042*	.*494*								
7. Age	Tau-b correlation	-.044	.025	.005	-.061	-.012	-.022	1.000						
	*P (two-tailed)*	.136	.390	.872	.075	.731	.482							
8. MSSs Wrists	Tau-b correlation	.079	-.104	.202	-.145	-.113	.019	-.004	1.000					
	*P (two-tailed)*	.009	.000	.000	.000	.001	.543	.886						
9. MSSs Shoulders	Tau-b correlation	.131	-.103	.295	-.132	-.046	.022	-.018	.398	1.000				
	*P (two-tailed)*	.000	.000	.000	.000	.167	.479	.551	.000					
10. MSSs Neck	Tau-b correlation	.139	-.112	.331	-.169	-.138	.009	-.028	.373	.624	1.000			
	*P (two-tailed)*	.000	.000	.000	.000	.000	.766	.334	.000	.000				
11. MSSs Head	Tau-b correlation	.073	-.106	.321	-.161	-.116	.046	-.119	.319	.414	.496	1.000		
	*P (two-tailed)*	.013	.000	.000	.000	.000	.126	.000	.000	.000	.000			
12. MSSs Upper back	Tau-b correlation	.131	-.096	.299	-.120	-.087	.010	-.072	.324	.558	.664	.481	1.000	
	*P (two-tailed)*	.000	.001	.000	.000	.009	.745	.013	.000	.000	.000	.000		
13. MSSs Lower-back	Tau-b correlation	.052	-.049	.234	-.151	-.128	.045	-.045	.314	.419	.412	.378	.419	1.000
	*P (two-tailed)*	.074	.079	.000	.000	.000	.138	.122	.000	.000	.000	.000	.000	

Abbreviations: ICT: Information and Communication Technologies; MWL: Mental Work Load; MSDs Musculoskeletal Symptoms

Note: Correlation indexes with a significance level of *p* < .05 have been highlighted.

In the group of men, the appearance of neck pain was the only MSSs that had significant correlations with computer usage time. In turn, we found no relevant correlations between the ratio of hourly pauses with any of the MSSs considered. However, the relevant correlations of the MWL levels with all the MSSs included were quite noteworthy. Likewise, the ergonomic conditions determined by adequate ICT devices showed negative correlations with all MSSs and adequate furniture showed negative correlations with MSSs in wrists, head, and lower-back. Age seemed to show a trend towards negative correlations with the MSSs, which only reached significance in MSSs in head. Intense physical activity outside of work in the men’s group showed a positive correlation with MSSs in wrists, neck, and lower-back.

The women’s correlation matrix showed relevant differences with the men’s matrix. First, computer usage time correlated with a greater number of MSSs and, specifically, with the appearance of pain in wrists, shoulders, neck, head, and upper back. The relationship between the ratio of pauses per hour and the appearance of MSSs was also clearer in women, as inverse significant correlations appeared between the ratio of pauses per hour and the appearance of MSSs in wrists, shoulders, neck, head, and upper-back. Ergonomic factors showed significant and negative correlations for all MSSs with the exception of shoulders in the case of adequate furniture. As in the men’s group, MWL correlated positively and significantly with of all MSSs although, in the women’s group, with the exception of wrists, the absolute value of the correlation indexes are higher for all MSSs. Ergonomic factors also showed negative and notable correlations with different MSSs in this group. Age showed only significant correlations with MSS in head and upper-back, with no relationships between intense physical activity outside the workplace and the emergence of MSSs in the group of women.

Covariance analysis was also performed, to clarify how the existing differences between the men’s and women’s groups in variables such as usage time, pauses, or age could condition the increased presence of MSSs in women. When covariance analysis was performed discounting the effect of these variables, the difference between men and women in the incidence of all relevant MSSs was maintained (F = 21.13; p < .001 for MSSs in neck; F = 19.20; p < .001 for MSSs in shoulders; and F = 20.47; p < .001 for upper back MSSs)

### 4.4. Structural equation modeling and mediation analysis

In this section, we analyzed the relationships between the selected indicators and the MSSs through their inclusion in structural equation models. Similarly, we analyzed the potential mediation effect of MWL on the relationship between computer usage patterns, and ergonomic and individual factors on the one hand, and MSSs on the other, proposed in the initial conceptual framework.

**Fig 3:**
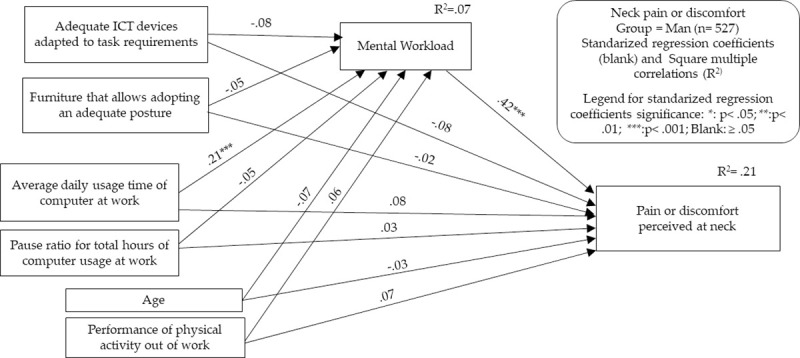
Structural equation modeling results. Neck MSSs in the men’s group.

**Fig 4:**
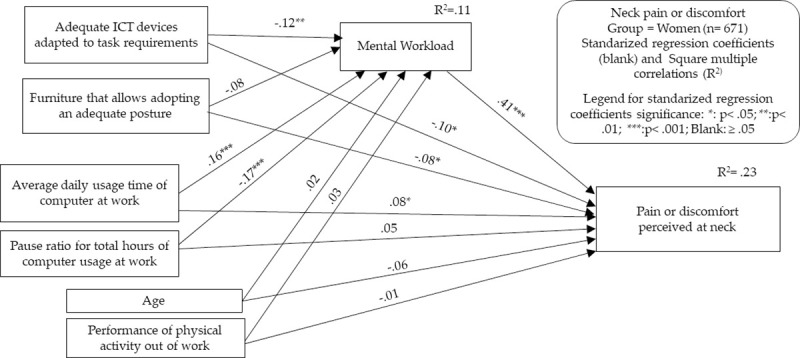
Structural equation modeling results. Neck MSSs in the women’s group.

**Fig 5:**
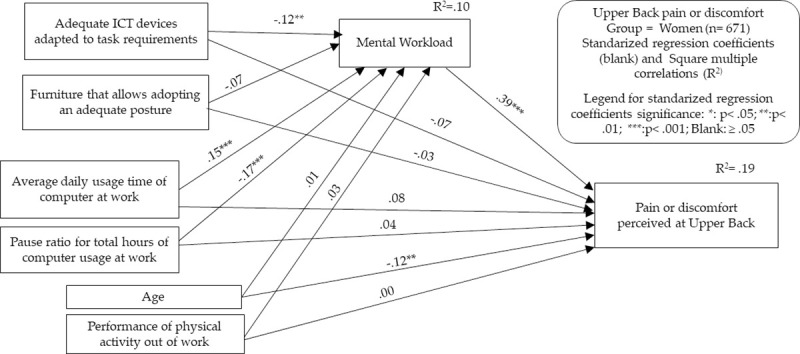
Structural equation modeling results. Upper back MSSs in the women’s group.

**Fig 6:**
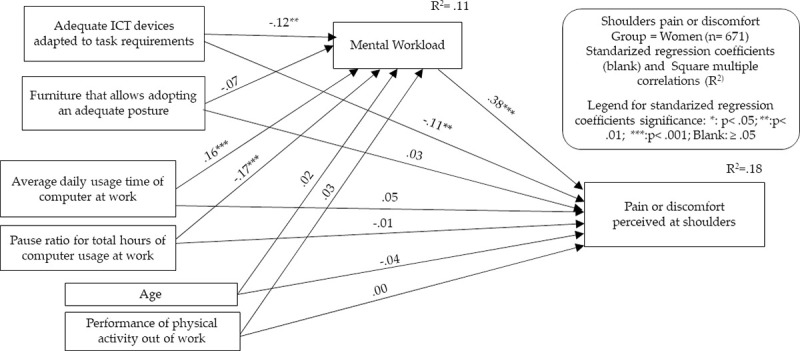
Structural equation modeling results. Shoulders MSSs in the women’s group.

To perform both types of analyses, we decided to select for each group only those MSSs that showed relevant relationship (P < .05 and correlation coefficient > .10 or < -.10) with the main predictor/independent variable of our theoretical framework: the total usage time of desktop and laptop computers. Contrary to the proposal in the classical formulation of Baron and Kenny [31], the current literature no longer considers it essential for significant relationships between the predictor/independent variable and the criterion/dependent variable to exist as a preliminary step to justify mediation analysis [34]. However, for reasons of parsimony and conceptual clarity, we drew on these requirements to provide only the relevant results. It was also considered that it is appropriate to use these criteria given the cross-sectional nature of our study, in which hard affirmations about unidirectional cause-effect relationships between indicators cannot be made. Therefore, models for the following MSSs were developed: neck in men, neck in women, and shoulders and upper back in women. In Figs 3–6 and Tables 7–10, the models generated are summarized, indicating in each one the standardized effects between the different indicators and the estimated percentage of explained variance (R^2^) for the endogenous variables. Maximum likelihood was chosen as the parameter estimation method, including means and intercepts estimation. For clarity, the estimated covariances between indicators are not included in the figures, but it should be noted that these are saturated models in which no parameter was fixed to 0. Therefore, the presentation of fit indicators is not necessary. It has also to be considered that maximum likelihood estimation procedure does not require the fulfillment of parametric assumptions. Complete statistical outputs of the exposed models can be found in S2 File.

**Table 7:** Mediation analysis results. Neck MSSs in the men’s group.

	Effects of: Daily usage time of computer				
MEDIATION ANALYSIS	On: Neck pain or discomfort				
	Mediated by: Mental Workload				
	Effect	SE	t	p	CI (p < .05)
Total effect	.179*	.058	3.069	.002	.064; .293
Direct effect	.126*	.054	2.325	.021	.019; .233
Indirect effect	.053*	.023	NP	NP	.008; .099
St. indirect effect	.045*	.019	NP	NP	.007; .086

Abbreviations: SE: standard error; CI: Confidence Interval; St.: Standardized; NP: Not provided by analysis

*: Non-zero (p < .05) effects

**Table 8:** Mediation analysis results. Neck MSSs in the women’s group.

	Effects of: Daily usage time of computer					Effects of: Pause ratio for hours of computer use				
MEDIATION ANALYSIS	On: Neck pain or discomfort					On: Neck pain or discomfort				
	Mediated by: Mental Workload					Mediated by: Mental Workload				
	Effect	SE	T	p	CI (p < .05)	Effect	SE	t	p	CI (p < .05)
Total effect	.243*	.069	3.542	.000	.108; .378	.030	.130	.235	.814	-.224; .285
Direct effect	.158*	.063	2.508	.012	.034; .282	.193	.138	1.398	.163	-.078;.464
Indirect effect	.085*	.030	NP	NP	.010; .051	-.162*	.091	NP	NP	-.393; -.032
St. indirect effect	.060*	.021	NP	NP	.019; .101	-.056*	.025	NP	NP	-.109; -.012

Abbreviations: SE: standard error; CI: Confidence Interval; St.: Standardized; NP: Not provided by analysis

*: Non-zero effects (p < .05)

**Table 9:** Mediation analysis results. Upper back MSSs in the women’s group.

MEDIATION ANALYSIS	Effects of: Daily usage time of computer					Effects of: Pause ratio for hours of computer use				
	On: upper back pain or discomfort					On: Upper back pain or discomfort				
	Mediated by: Mental Workload					Mediated by: Mental Workload				
	Effect	SE	t	P	CI (p < .05)	Effect	SE	T	p	CI (p < .05)
Total effect	.210*	.061	3.419	.001	.090; .331	-.010	.132	-.078	.938	-.269; .248
Direct effect	.122*	.058	2.104	.036	.008; .236	.130	.123	1.057	.291	-.112; .372
Indirect effect	.088*	.027	NP	NP	.037; .145	-.140*	.077	NP	NP	-.322; -.032
St. indirect effect	.062*	.019	NP	NP	.026; .101	-.046*	.020	NP	NP	-.089; -.011

Abbreviations: SE: standard error; CI: Confidence Interval; St.: Standardized; NP: Not provided by analysis

*: Non-zero effects (p < .05).

**Table 10:** Mediation analysis results. Upper back MSSs in the women’s group.

MEDIATION ANALYSIS	Effects of: Daily usage time of computer					Effects of: Pause ratio for hours of computer use				
	On: Shoulders pain or discomfort									
						On: Shoulders pain or discomfort				
	Mediated by: Mental Workload									
						Mediated by: Mental Workload				
	Effect	SE	t	P	CI (p < .05)	Effect	SE	t	p	CI (p <. 05)
Total effect	.180*	.071	2.526	.012	.040;.319	-.145	.125	-1.164	.245	-.390; .100
Direct effect	.099	.066	1.513	.131	-.030; .228	.011	.127	.085	.932	-.239; .260
Indirect effect	.080*	.010	NP	NP	.009; .046	-.156*	.087	NP	NP	-.373; -.031
St. indirect effect	.054*	.019	NP	NP	.018; .093	-.052*	.023	NP	NP	-.100. -.011

Abbreviations: SE: standard error; CI: Confidence Interval; St.: Standardized; NP: Not provided by analysis

*: Non-zero effects (p < .05)

A summary table of the mediation analysis has been included at the bottom of each figure. This mediation analysis was carried out following the guidelines proposed by Hayes and Rockwood [34]. In our case, we intended to explore the extent to which the MWL acts as a mediating variable of the effect of computer usage patterns in the appearance of MSSs. Conceptually, this implies contrasting the extent to which the effect of computer usage in the emergence of MSSs occurs not only directly, but also through its relationship with an increase in workers’ MWL. It also implies showing what part of the potential effect of MWL on MSSs is due to its modification because of the pattern of computer usage and what part is derived from its modification due to other factors. For the statistical interpretation of these conceptual relationships, the estimation of several parameters is presented, carried out through bootstrapping procedures with the PROCESS macro for SPPS developed by Hayes and Preacher [45]. These parameters and their interpretation are as follows: 1) Total effect: it measures the relationship of the computer usage pattern as a whole with the appearance of each MSS, including both their direct relationship and the relationship derived from the association with MWL; 2) Direct effect: it measures the direct relationship between the computer usage pattern and the appearance of each MSS; 3) Indirect effect: it measures the relationship of the computer usage pattern with the appearance of each MSS based on their relationship with MWL; 4) Standardized indirect effect: it represents the previous standardized parameter, thus allowing a better comparison with the remaining standardized effects by neutralizing the effects of the differences in the measurement dimension. The type of analysis performed establishes the significance of the mediation effect through the analysis of the confidence intervals of the indirect effect. In this way, a mediation effect will be significant when the confidence interval of the indirect effect (standardized or unstandardized) does not include zero. In the group of men, mediation analysis was performed only for the conjoint time of laptop and desktop usage, as the ratio of pauses per hour did not present significant relationships with neck MSS. In contrast, in the women’s group, mediation analyses were performed both for the total laptop and desktop usage time and the hourly pause ratio, as they did show a relationship with the appearance of the selected MSSs.

Concerning the first model, which analyzed the appearance of MSSs in the neck in the men’s group, the direct effects of the pattern of computer usage and the appearance of neck symptoms did not reach significance. However, the relationships between daily usage time of computer and MWL and the relationships between MWL and the emergence of MSSs in the neck reached significance. The set of contemplated factors, in any case, explained just over 20% of the variance of neck MSSs. The analysis showed that MWL acts potentially as a mediator of the relationship between the pattern of computer usage and the appearance of pain or discomfort in the neck, although the dimension of the indirect effect was low. Nevertheless, the indirect effect/total effect ratio of computer usage time on MSSs shows that the indirect effect of computer usage time accounts for 30% of neck MSSs in men through MWL. In any case, only 11% of MWL was explained by the variables included in the model.

The model of neck MSSs in women showed a greater number of relevant relationships. The strongest association with neck pain or discomfort occurred through MWL, but computer usage time and ergonomic factors maintained significant but smaller relationships. Likewise, the use of adequate ICT devices and the two indicators of computer usage pattern maintained significant relationships with MWL. The indirect effects analysis showed that MWL acts potentially as a mediator for the two usage pattern indicators. In the case of usage time, the indirect effect accounted for approximately one-third of the total effect. In the case of the ratio of hourly pauses, the indirect effect was very relevant and accounted for 84% of the total effect. As these are opposite-direction effects, the direct effect of the number of pauses on the reduction of neck MSSs is masked by the indirect effect produced through the reduction of MWL. Nevertheless, the indirect effect/total effect ratio of computer usage time on MSSs shows that the indirect effect of computer usage time accounts for 42% of neck MSSs in women through MWL. As in all the other models, approximately 10% of MWL was explained by the variables included in the model.

In the model of the appearance of MSSs in the shoulders in the group of women, the pattern was very similar to the previous model, only with lower effects. Again, the potential mediation effects of MWL achieved significance both on the effect of usage time and the hourly pause ratio. As in the case of the neck, the indirect effect of the ratio of pauses per hour was greater than the direct effect, as its effect on MWL seemed to mask the direct effect on MSSs. The indirect effect/total effect ratio of computer usage time on MSSs shows that the indirect effect of computer usage time accounts for 42% of upper-back MSSs in women through MWL.

Finally, in the model referring to MSSs in upper back, the patterns found in the group of women for other MSSs were also reproduced with slight variations. The potential mediating effect of MWL on the usage time was again evident, since the indirect effect of computer usage time on MSSs accounts for 44% of shoulders MSSs in women through MWL. There was also evidence of an important mediating effect on the total effect of the ratio of pauses per hour. Additionally, an inverse association with age in the appearance MSSs in the upper back.

## 5. Discussion

This study analyzed the relationship between various factors and the appearance of different MSSs in computer workers, jointly considering the associations between ergonomic elements, usage patterns, and psychological and individual factors. The purpose was not so much to comprehensively collect all the factors involved in the emergence of MSSs but to perform a detailed analysis of the inter-relationships between the factors of different levels and their differential association with these disorders. Specifically, we focused on examining the potential mediation role of psychological factors in the effect of temporal computer usage patterns, focusing on a relevant construct in the field of ergonomics, the MWL. Our results offer interesting evidence, which must be contextualized and interpreted.

As a starting point, the results showed that MSSs in the neck area have the highest incidence of the sample of workers. In our case, a probabilistic sample to make inferences about the general working population was not used, but these data reflect the general pattern that indicates this area as the most vulnerable in workers using ICTs in different epidemiological studies [38, 55]. On the other hand, the overall view of our results showed that men and women have different incidences and association patterns of MSSs. As the covariance analysis showed, the differences of MSSs incidence between men and women in our sample cannot be explained by differences in usage time, pauses, or age. As different studies show [13, 27, 31, 56, 57], sex is a distinctly discriminant category in the emergence of MSSs. However, it represents a substantively invariant factor with scarce explanatory potential because it joins biological and cultural elements. Therefore, performing separate analyses for groups of men and women is highly recommended, as we have done, taking as reference other authors [27, 31].

The initial exploration of the bivariate relationships has only revealed relevant associations between total computer usage time and MSSs in some areas, specifically in the neck in men, and in the neck, shoulders, and upper back in women. These relationships are consistent with the existing literature that indicates, on the one hand, a higher incidence of MSSs in women using the computer and, on the other hand, greater impairment of these areas in the general population of workers using the computer [27, 56, 57]. On the other hand, bivariate relationships showed the important association of MWL with the emergence of MSSs, with correlations in women exceeding values of .40 in all cases, except for MSSs in wrists, and slightly more moderate values in men.

The examination of the models generated from our data also offers a variety of interesting results. In any case, when interpreting these results, we must be cautious in terms of causal inference. Our design, of a cross-sectional nature, does not allow for the establishment of cause-and-effect relationships. In turn, no evidence in the literature clearly supports the existence of a causal or one-way relationship between psychosocial and ergonomic factors, on the one hand, and the appearance of MSSs, on the other [6–9]. Therefore, there is no clear theoretical reference that we can use to interpret the direction of the effects shown by the numerical relationships. This implies that certain effects must be interpreted as potential two-way relationships between factors.

The results supported, in the first instance, some of the relationship hypotheses contained in the initial theoretical framework. Overall, as the structural equation models showed, all of the factors explained about 20% of the variance of the occurrence of MSSs. This percentage, although relevant, was small and points to the complexity of predicting such disorders. It should be noted that the methodological approach we used, consisting of the collection of general indicators from self-reports in a broad sample, allows the development of complex models that simultaneously integrate multiple indicators but it does not achieve the accuracy that could be derived from the study with objective physical and ergonomic assessments [58, 59]. In this sense, the influence of relevant factors that have been measured by means of subjective scales could have been underestimated in our models.

From a global view, the four models presented followed common general patterns, although they vary in the weight of the different indicators of the different areas considered and between men and women. The existence of adequate devices and furniture showed a clear trend of inverse relationships with the appearance of the selected MSSs, although these relationships reached significance only in some of the parameters contained in the models. This result was consistent with the existing literature, which points out that the ergonomic adequacy of ICT devices is related to a lower incidence of MSSs in different body areas, although the causal evidence of this relationship is still inconsistent [8, 12, 16, 18, 34]. As we have pointed out, the fact that the data were collected by self-report and generically could be conditioning the small size of the effects found.

The effect of usage time on the emergence of MSSs was small in the different models, as already anticipated by the bivariate correlations. This fact must be interpreted considering the knowledge that the time of computer usage is only one of the possible factors involved in MSS appearance and that its causal relationship with MSSs is the subject of controversy [6–8]. In turn, it is necessary to analyze this effect, as we will do below, in light of the possible mediating role of psychological factors. The ratio of hourly pauses, a factor whose causal relationship with MSSs is disputed [13, 17], showed no clear effects on the emergence of MSSs in the models. However, as we shall see, it presented more complex relationships when mediation analysis was performed. It should be noted that, as has been included in the hypotheses, computer usage time showed clear and positive relationships with MWL in all the models, and the pause ratio showed an inverse relationship with MWL in the case of women. These results support the need to consider psychological processes not only as potential risk factors in the production of MSSs in computer workers but also as factors that are affected by computer usage itself. The psychological distress produced by computer usage has been analyzed in various studies [60–62]. However, to our knowledge, no empirical work has integrated in a global model both the effect of computer usage on psychological distress and the effect of psychological distress on MSSs, as proposed by Wahlström [20] in his model and as we reflect in our theoretical framework. In turn, it is noteworthy that the existence of adequate devices was associated with lower levels of MWL in the case of women. These relationships are of great interest, as they will support the possible mediating effect of MWL in the emergence of MSSs.

The generated models reflected the clear positive effect of MWL on the emergence of MSSs in all cases. This effect was of a substantially greater magnitude than the rest of the factors, including usage patterns. This result supports the validity of the MWL construct in the study of MSSs and represents in itself further evidence of the relevant role of psychological and organizational factors in the emergence of MSSs in computer workers. This role has already been highlighted in other studies using concepts linked to psychological distress in the work environment such as job-strain [25, 29, 54], emotional disturbance and intense effort at work [29], anxiety [63, 64], over-commitment and perceived stress [25] or psychological distress [24].

In any case, the most relevant results of our work emerged in the analysis of the inter-relationships between the different factors in their influence on the appearance of MSSs and, specifically, the potential mediating effect of MWL. Thus, we have shown that the indirect effect of computer usage time in the appearance of MSSs through its association with the MWL accounted for relevant percentages of the appearance of MSSS in neck (men and women) and upper-back and shoulders (women). These results suggests that prolonged use of the computer would have at least two important effects that should be considered in the study of MSSs: 1) A potential direct effect that would be linked to a physical and ergonomic dimension, as the literature proposes, related to static postures, repetitive tasks, and awkward positions; 2) A potential indirect effect, firstly, through its contribution to workers’ psychological stress and, secondly, to the effect of stress on the physical burden and the perception of muscle tension, as proposed in the model of Wahlström [20].

In the case of the ratio of pauses per hour, we found no indirect effect in men, but we did find one in women for the three MSSs analyzed (neck, shoulders, upper back). In the latter case, these are opposite-direction effects, as a higher rate of pauses per hour is related to lower MWL, but a higher ratio of pauses per hour seems to be related to a higher incidence of MSSs. This shows that the effect between pauses and MSSs, which is close to zero, was explained by the opposite-sign relationships that come together. Thus, the direct relationship between the ratio of pauses per hour and MSSs showed the opposite direction to that hypothesized in our theoretical framework. This result, however, has been found in some cross-sectional studies in the literature [65]. A potential explanation for this would be to assume that pauses could be partly a result of the emergence of MSSs and not just a reducing factor of them. In this sense, women with MSSs may be forced to take a greater number of pauses to maintain their activity. Additionally, such pauses during computer usage time could contribute to reducing MWL levels. In the case of neck and upper back in women, the absolute magnitude of the direct and indirect effect is very similar, with the indirect effect for MSSs in shoulders being substantively lower.

Overall, even taking into account the precautions we have outlined at the beginning, the mediation analyses carried out showed that a relevant part of the total relationship of computer usage patterns with MSSs should be analyzed through their relationship with MWL and conjointly, also considering the relationship of MWL with MSSs. This has important implications, as it indicates that some controversial results in the literature about the effect of certain factors on MSSs may be due to the inadequate consideration of the overall effect of the psychological factors. It also suggests that, in preventive terms, the effect of certain ergonomic interventions may be ineffective if the simultaneous effect of other variables that may be affecting the psychological and organizational dimension is not controlled. As we have seen, only a small part of MWL, which has a relevant association with MSSs, is explained by computer usage itself.

In any case, we close this discussion section by commenting on some of the limitations that must be considered when interpreting the results of the work. Firstly, its cross-sectional design, which prevents interpretations about one-way cause-effect relationships and forces the interpretation in terms of association or potential bidirectionality of the relationships. Secondly, data collection through self-report, which induces subjectivity in the indicators. This limitation affects the reliability of the assessment of ergonomic factors, which has been performed by means of global evaluations made by the workers themselves. Thirdly, the option of collecting a limited number of indicators which, despite allowing for a more parsimonious analysis of the relationships, has a more moderate explanatory power for the disorders. Fourthly, the fact that our work has focused specifically on the use of two specific devices, laptop and desktop computers, which have shown a higher relationship with the incidence of MSSs in the work environment; but we did not cover the full range of ICT devices that has been incorporated into the work setting and whose analysis will require further study. Although the MWL indicator integrates different psychosocial and organizational stressors that could influence the appearance of MSSs, we have not considered the wide range of constructs and variables that may be included within this set of factors [66, 67]. In this sense, our study aims to offer an initial exploratory approach to the global study of complex relationships between the considered indicators, but it must be complemented with further works that integrate objective measures referring to more specific patterns of computer usage in reduced samples and broader psychosocial and organizational measures. Finally, future studies should progressively develop complex models in which additional factors that appear related to MSDs and MSSs in the literature (i.e. lifestyle factors, health status and comorbidity) [9, 13, 67] are included.

## 6. Conclusions

The study of the risk factors for the onset of MSSs in computer workers should be carried out not only with the inclusion of factors related to different levels, including usage patterns, ergonomic factors, organizational/psychological factors, and individual factors. It should also deal with the analysis of the relationships between the different factors and their potential interaction effects, as these are essential to understand how they relate to the emergence of MSSs.

There are relevant differences between men and women in the patterns of onset and the relationship of MSSs with risk factors. Regardless of the higher incidence of MSS in women, which could be conditioned by the confluence of other factors, our results show a clearer positive relationship between usage time and the emergence of MSS in the neck area, shoulders, and upper back in the group of women. They also show a clearer inverse relationship in women between the frequency of pauses per computer usage hours and the appearance of MSSs in the mentioned areas.

Psychological overload levels, operationalized through the MWL measurement, have stronger direct positive relationships with the emergence of MSSs than the remaining factors considered. Direct relationships of computer usage time patterns, ergonomic factors, and individual factors show weaker relationships that, in certain bodily areas, do not reach significance in the appearance of MSSs.

The mediation analysis shows that, despite the low effect of usage patterns on the appearance of the MSSs studied, their total effect on MSSs is greater due to the contribution of the indirect effects of MWL. This implies that a relevant part of the effect of computer usage patterns on the emergence of MSSs can be understood from the relationship of usage patterns with MWL and the subsequent direct relationship of MWL with MSSs. This result has important implications, as it suggests that some of the inconsistent results found in the literature on the effect of computer usage time on MSSs may be due to the inadequate consideration of the direct and indirect effects of the psychological factors. On the other hand, it indicates that, when designing preventive strategies, the effect of purely ergonomic interventions may be limited if the simultaneous effect of other variables that may be affecting the psychological and organizational dimensions are ignored.

For a better understanding of and intervention in the emergence of MSSs, it is necessary for research in this area to draw on multilevel theoretical models that integrate and analyze in detail the relationships between the different levels of the risk factors.

## Supporting information

S1 File Partial regression analysis between MSD, time of computer usage and mental work load. (PDF)

S2 File Structural equation modelling MSD—global output. (PDF)
