# Supplementary material for: Do psychosocial factors mediate the appearance of musculoskeletal symptoms? Evidence of an empirical study about the role of mental workload in computer workers
Source: PLoS One. 2021 Jun 17;16(6):e0252179. doi: 10.1371/journal.pone.0252179 (PMC8211229; doi:10.1371/journal.pone.0252179)
Supplement: S1 File — (PDF) [file pone.0252179.s001.pdf]

S1 File- Partial regression Plots between Musculoskeletal Symptoms (MSSs) and Average daily time of computer use and Mental Work Load (MWL) Factor

Women’s Group

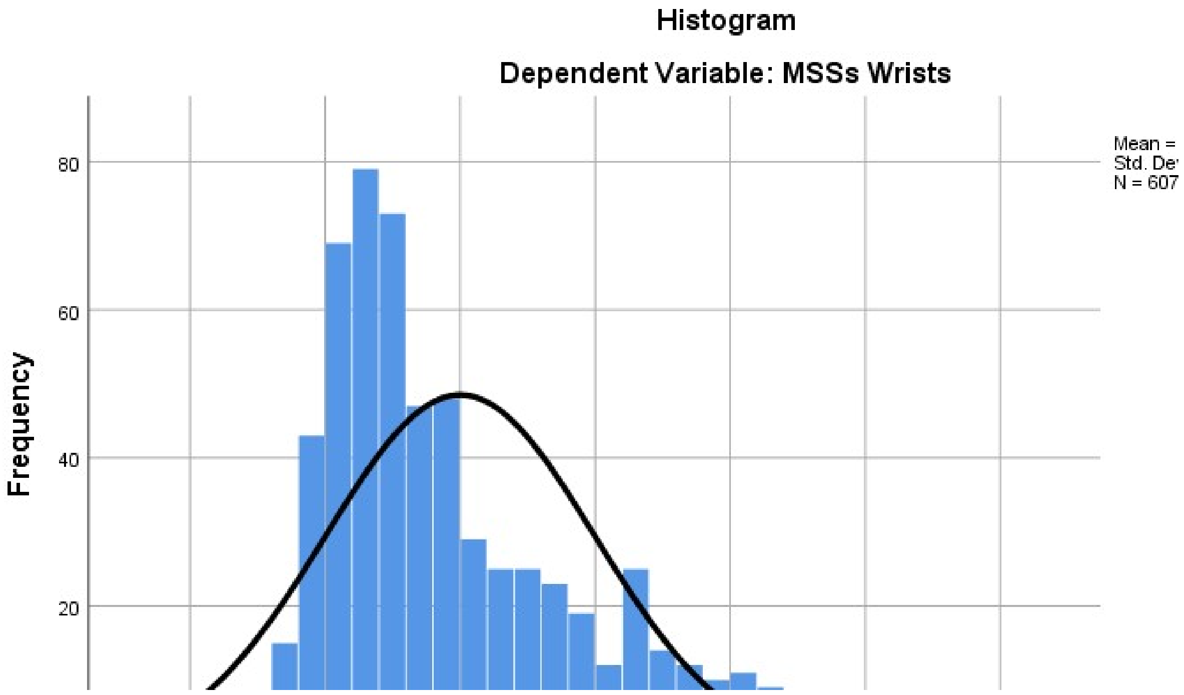

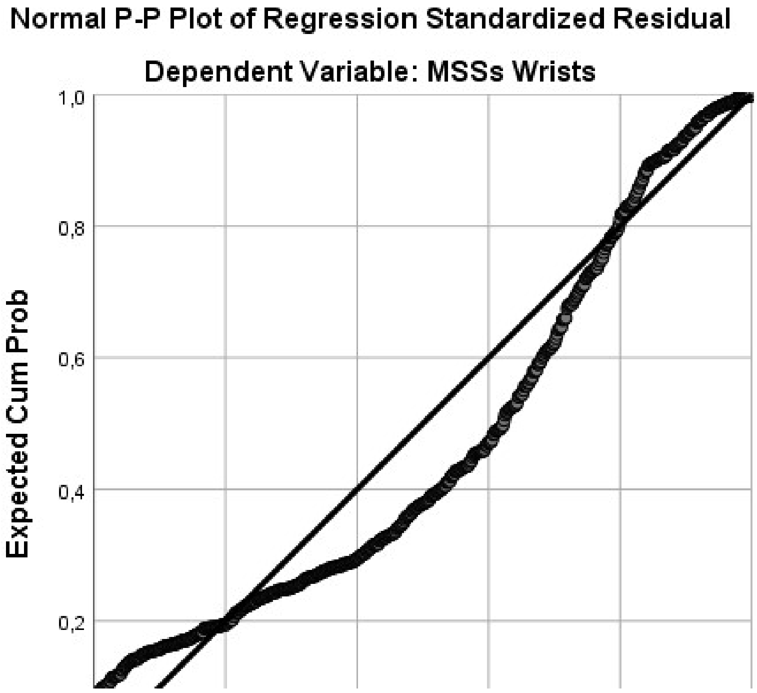

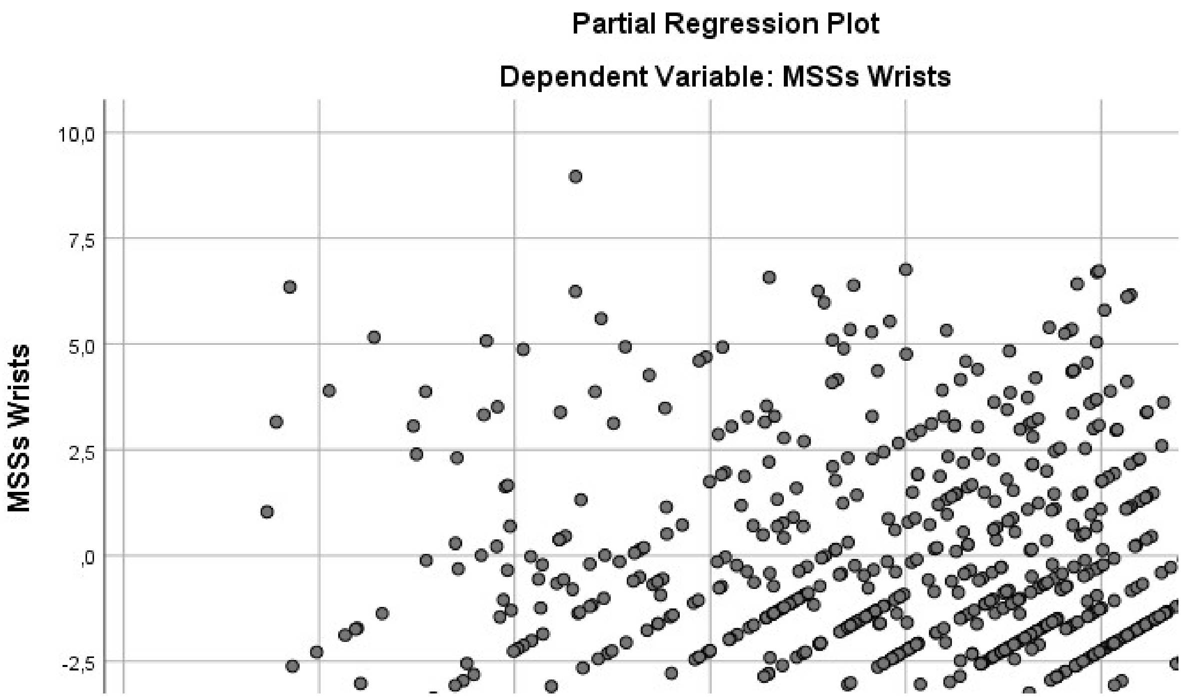
Partial Regression Plot
Dependent Variable: MSSs Wrists
MSSs Wrists
10,0
7,5
5,0
2,5
,0
-2,5

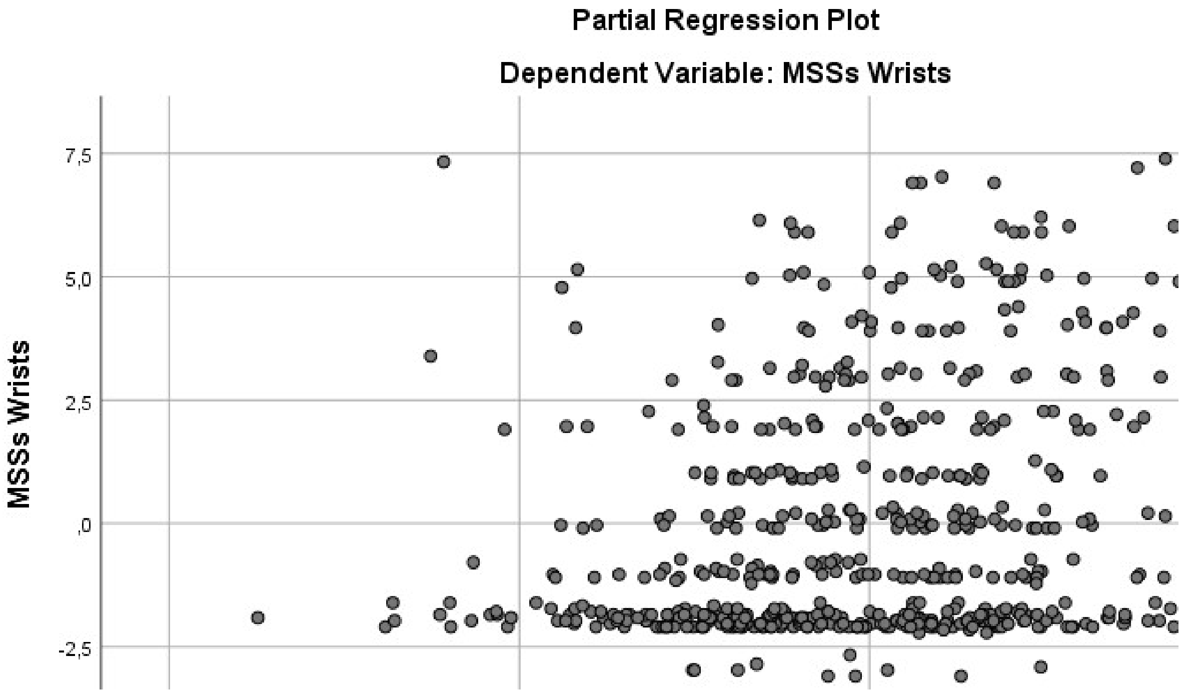
Partial Regression Plot
Dependent Variable: MSSs Wrists
MSSs Wrists
7,5
5,0
2,5
,0
-2,5

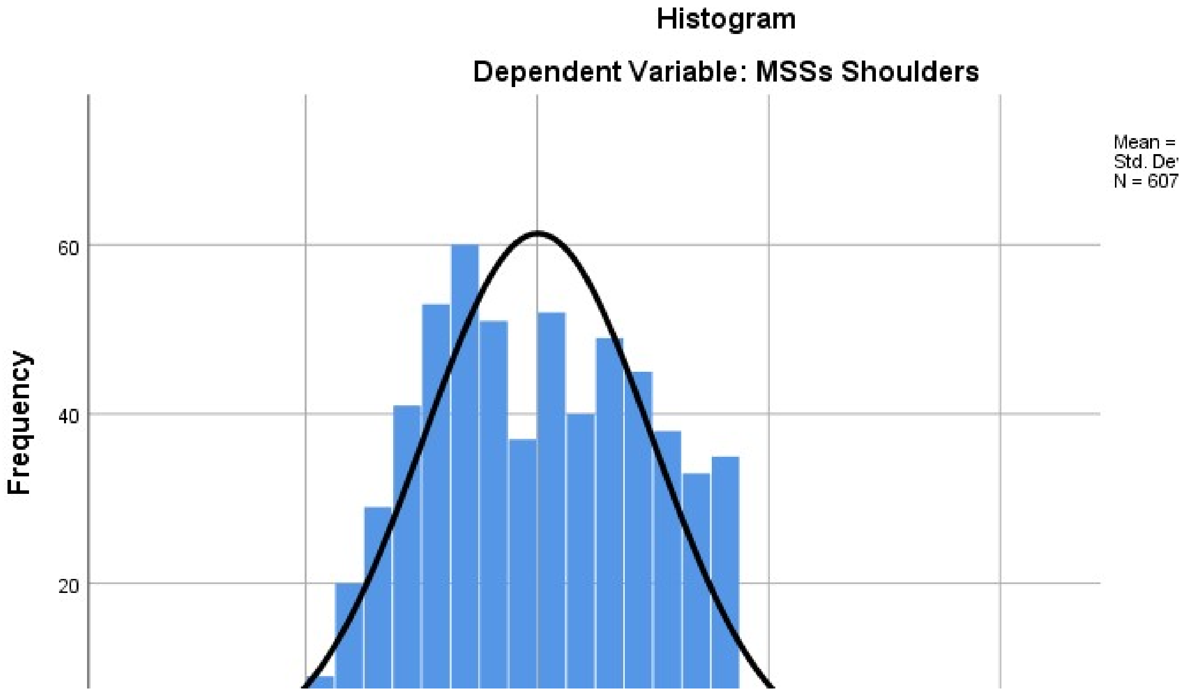
Histogram
Dependent Variable: MSSs Shoulders
Mean =
Std. De
N = 607
60
40
20
Frequency

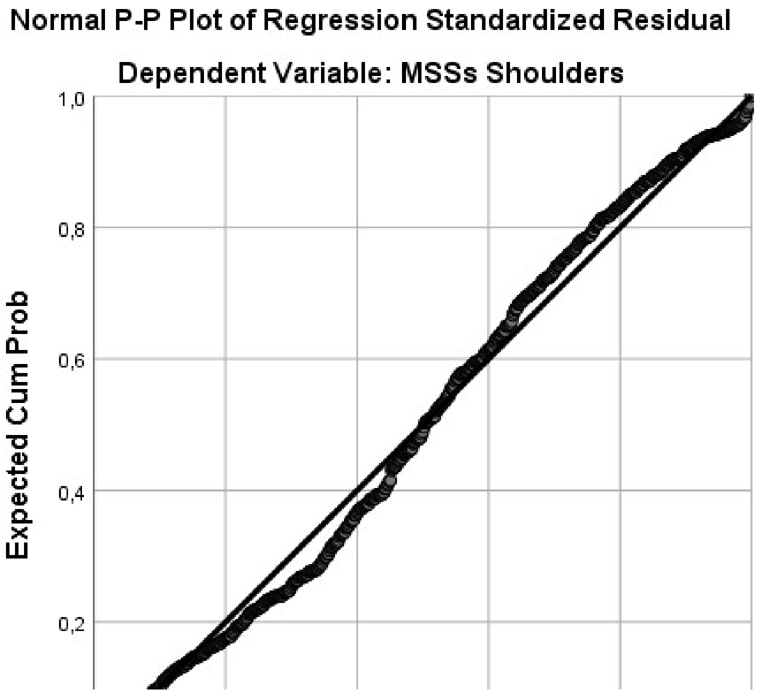
Normal P-P Plot of Regression Standardized Residual
Dependent Variable: MSSs Shoulders
1,0
0,8
0,6
0,4
0,2
Expected Cum Prob

Partial Regression Plot

Dependent Variable: MSSs Shoulders

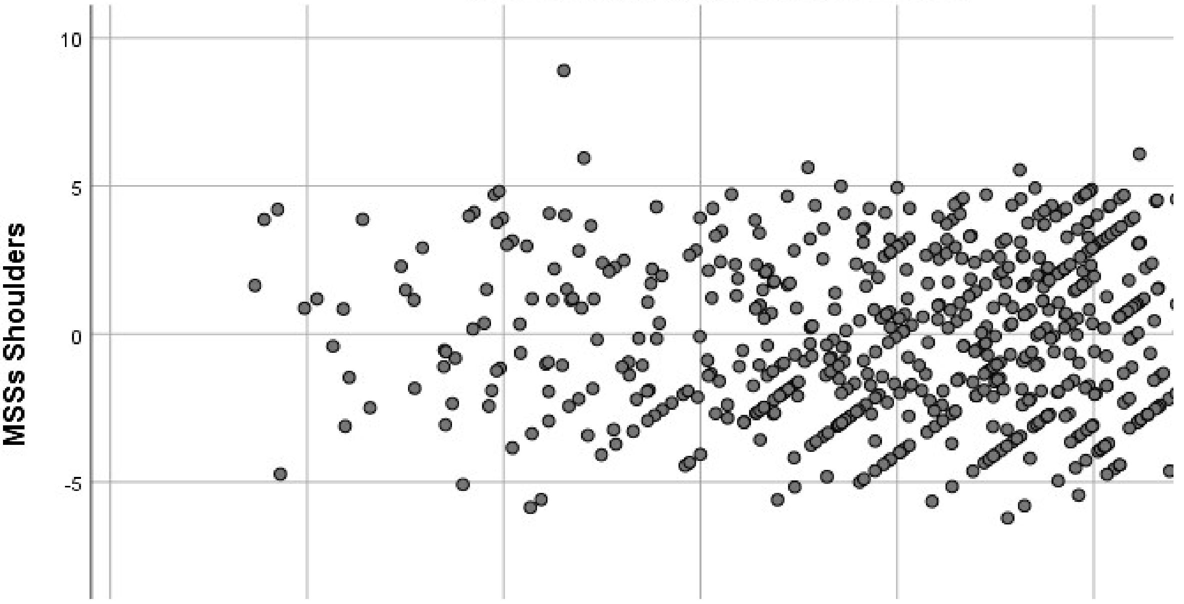

Partial Regression Plot

Dependent Variable: MSSs Shoulders

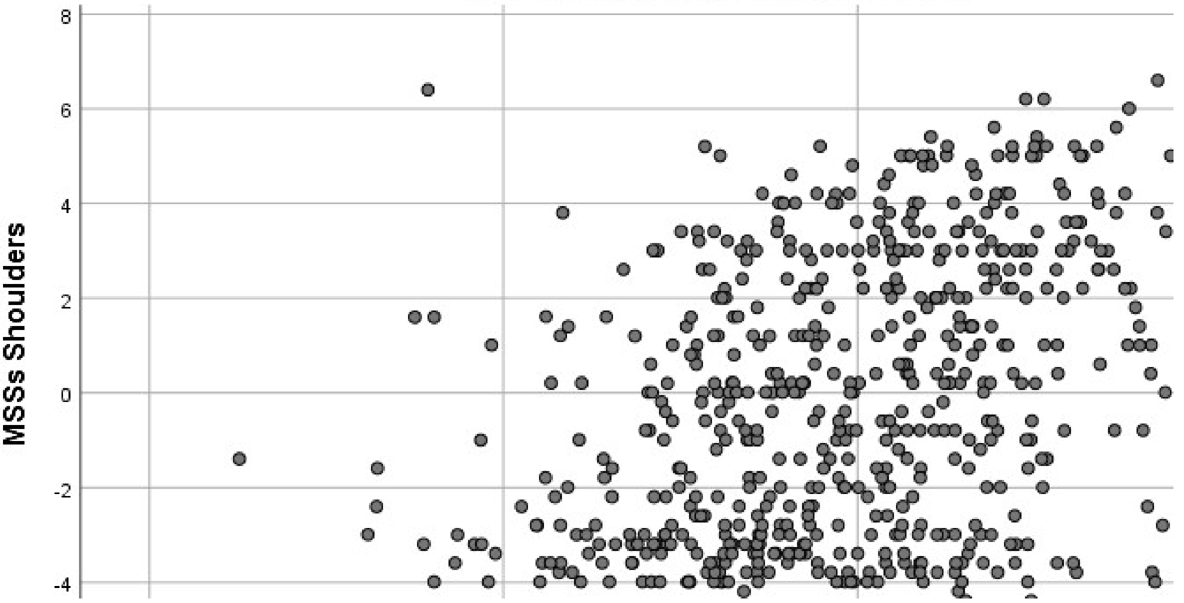

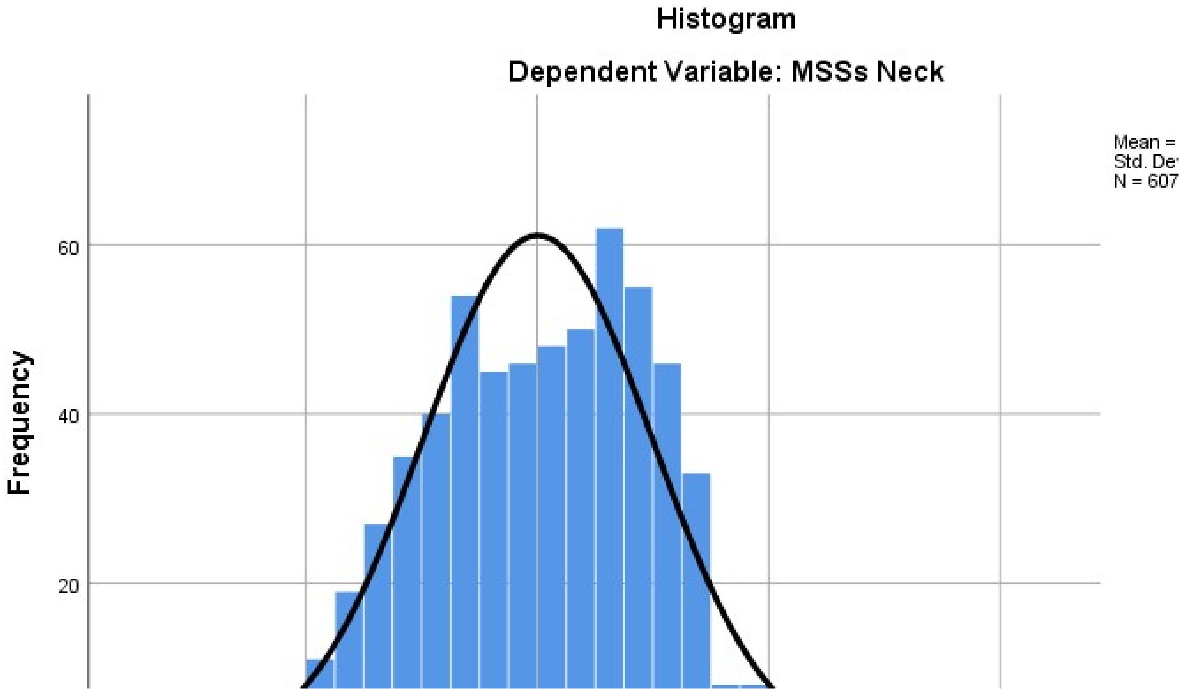
Histogram
Dependent Variable: MSSs Neck
Frequency
60
40
20
Mean =
Std. Dev
N = 607

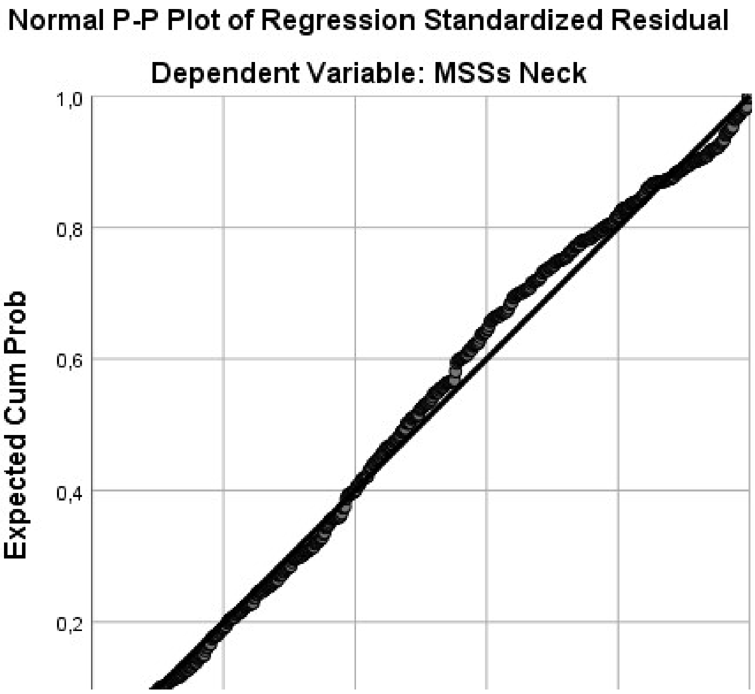
Normal P-P Plot of Regression Standardized Residual
Dependent Variable: MSSs Neck
Expected Cum Prob
1,0
0,8
0,6
0,4
0,2

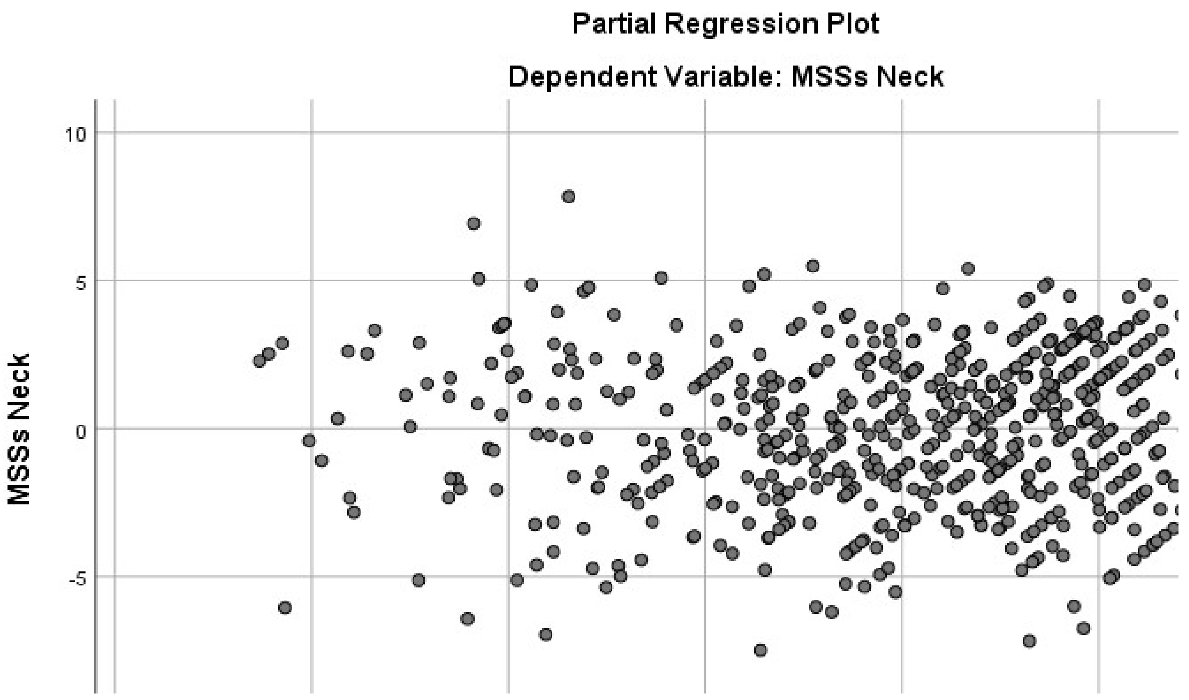
Partial Regression Plot
Dependent Variable: MSSs Neck
10
5
0
-5
MSSs Neck

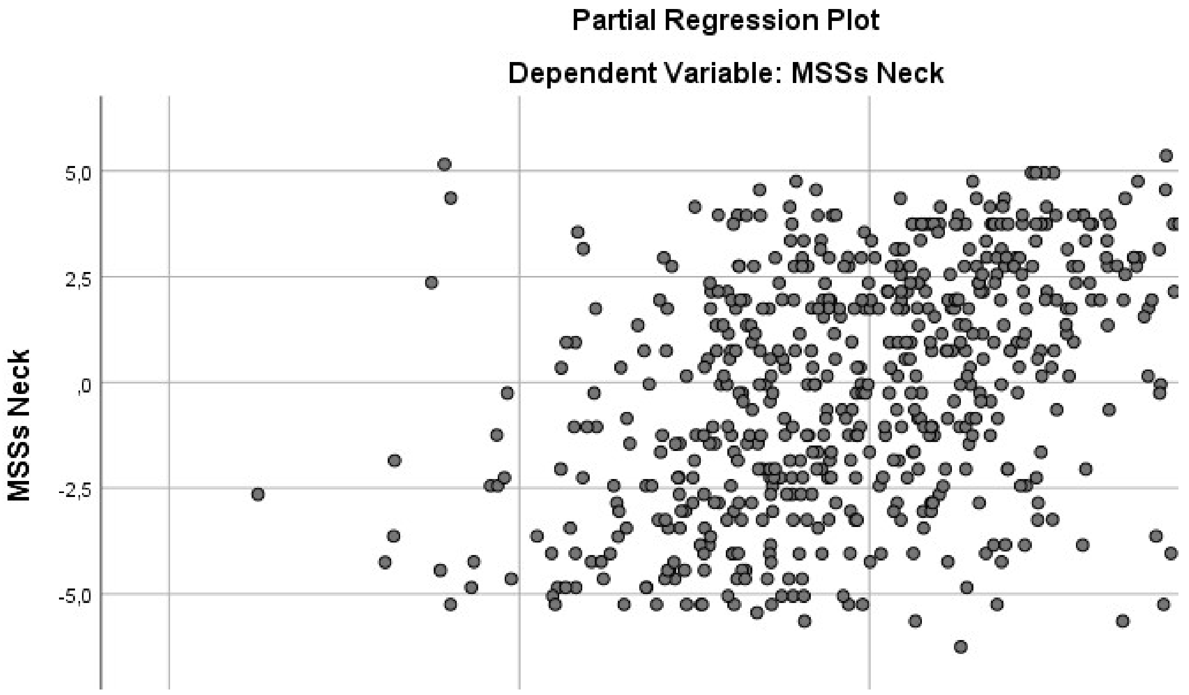
Partial Regression Plot
Dependent Variable: MSSs Neck
5,0
2,5
,0
-2,5
-5,0
MSSs Neck

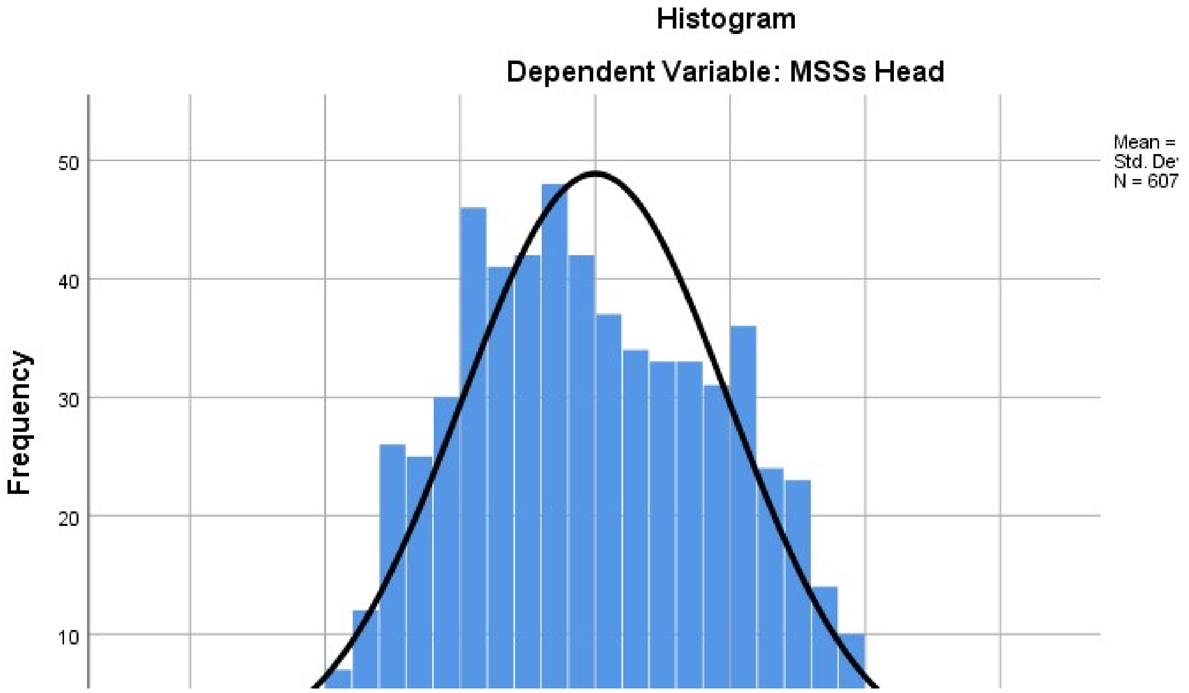
Histogram
Dependent Variable: MSSs Head
Frequency
50
40
30
20
10
Mean =
Std. De
N = 607

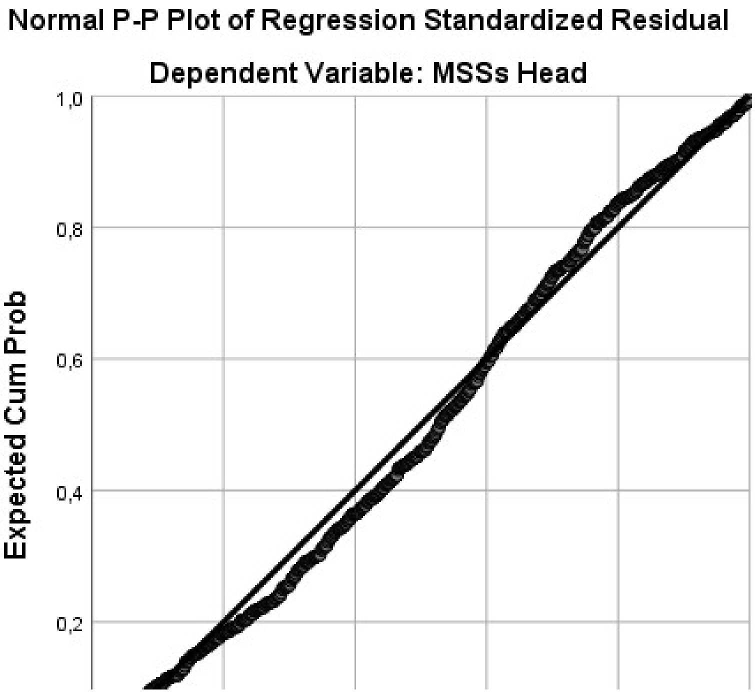
Normal P-P Plot of Regression Standardized Residual
Dependent Variable: MSSs Head
Expected Cum Prob
1,0
0,8
0,6
0,4
0,2

Partial Regression Plot

Dependent Variable: MSSs Head

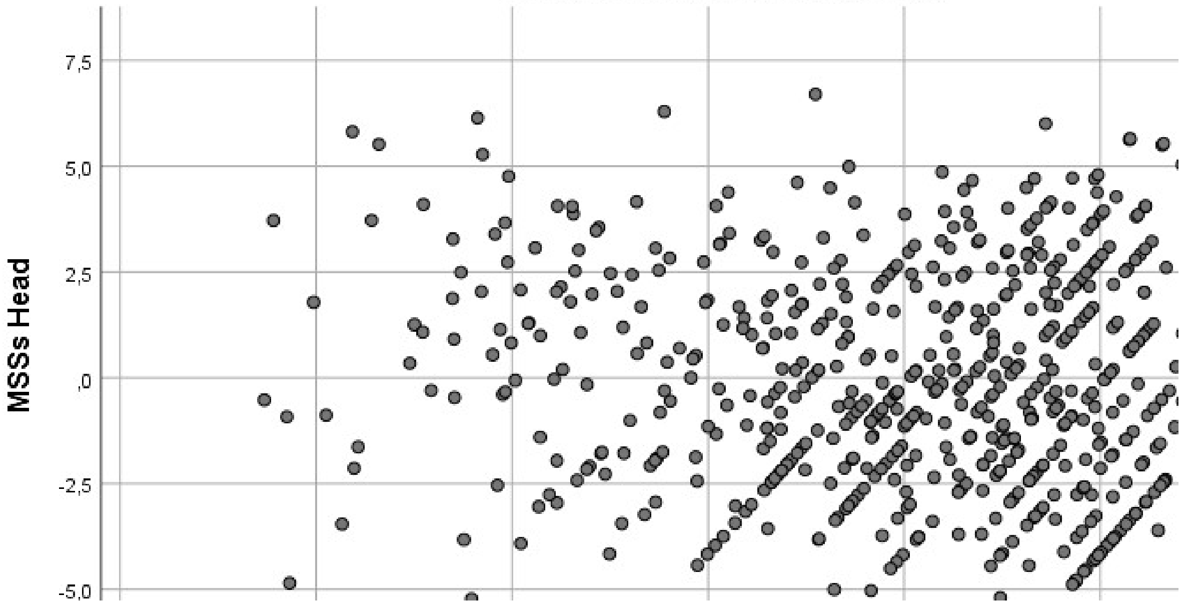

Partial Regression Plot

Dependent Variable: MSSs Head

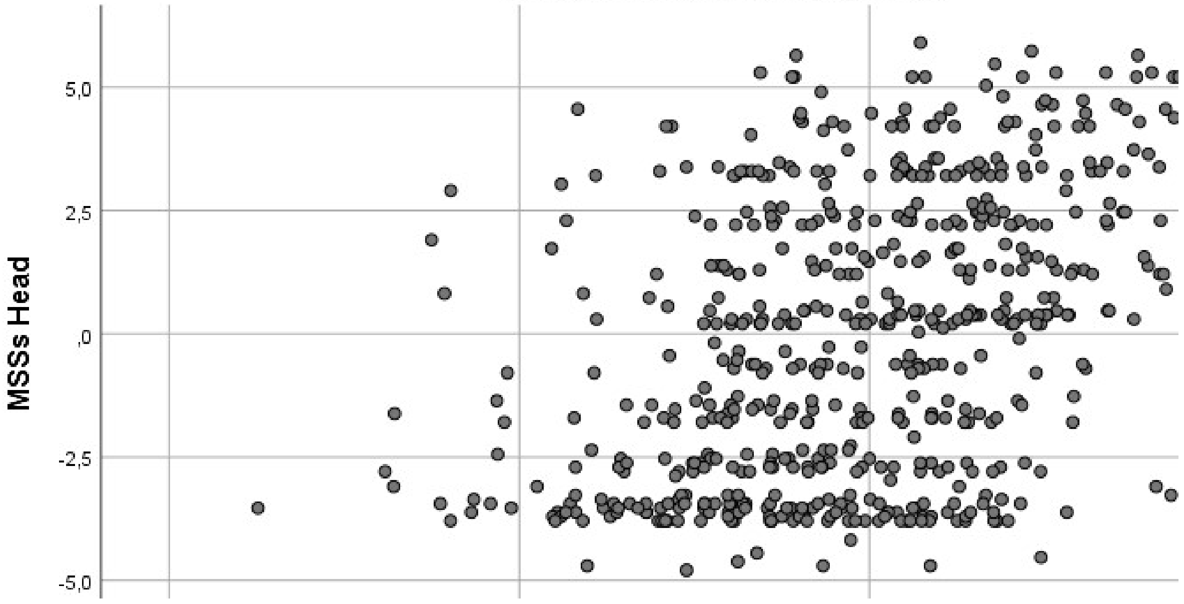

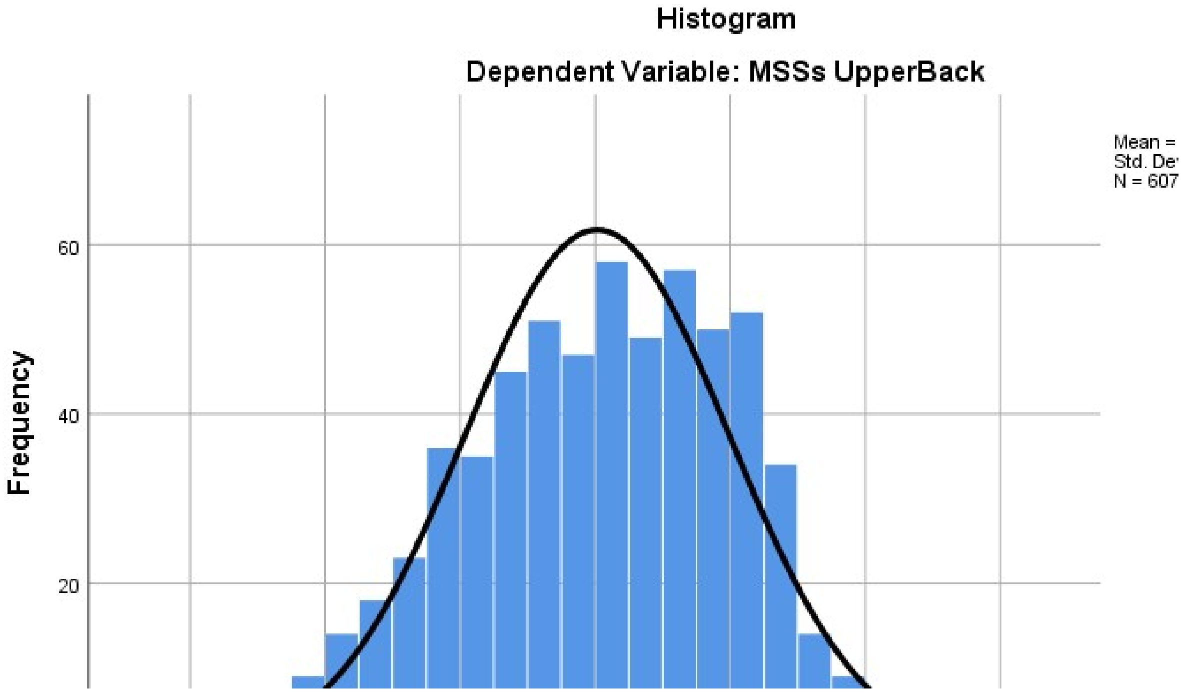
Histogram
Dependent Variable: MSSs UpperBack
Mean =
Std. De
N = 607
60
40
20
Frequency

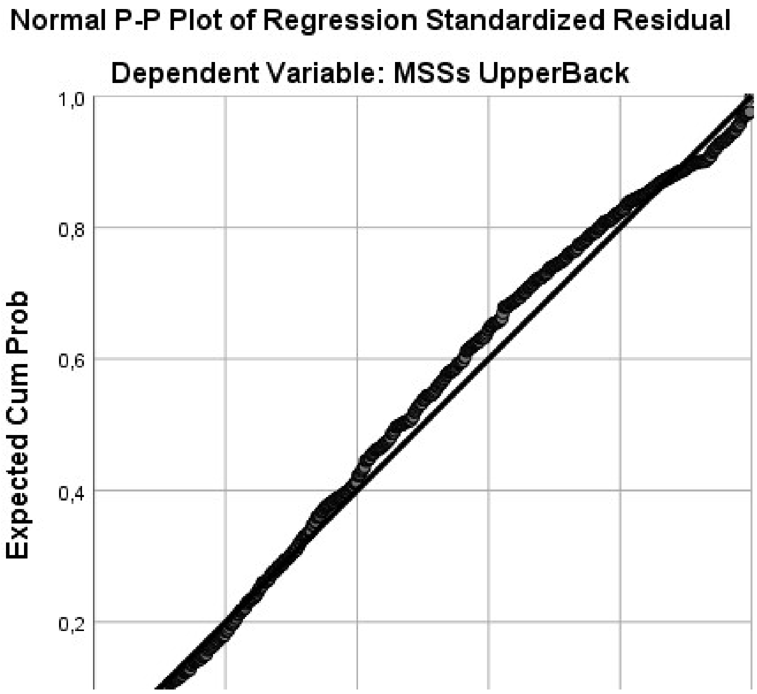
Normal P-P Plot of Regression Standardized Residual
Dependent Variable: MSSs UpperBack
1,0
0,8
0,6
0,4
0,2
Expected Cum Prob

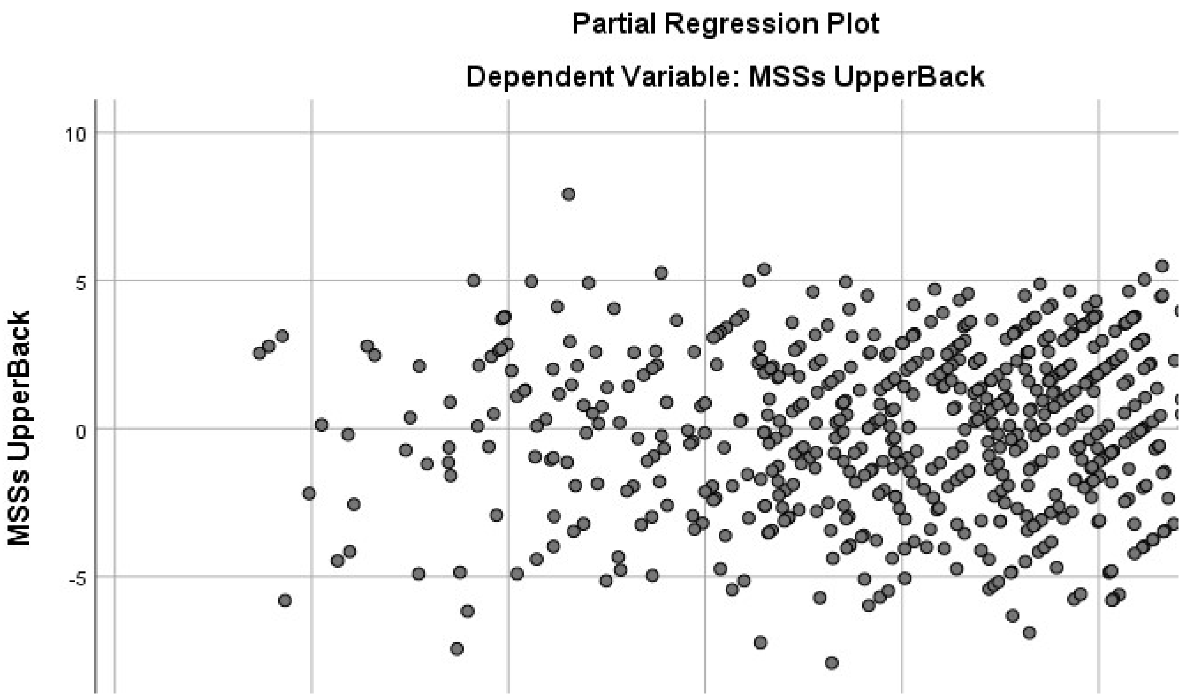
Partial Regression Plot
Dependent Variable: MSSs UpperBack
10
5
0
-5
MSSs UpperBack

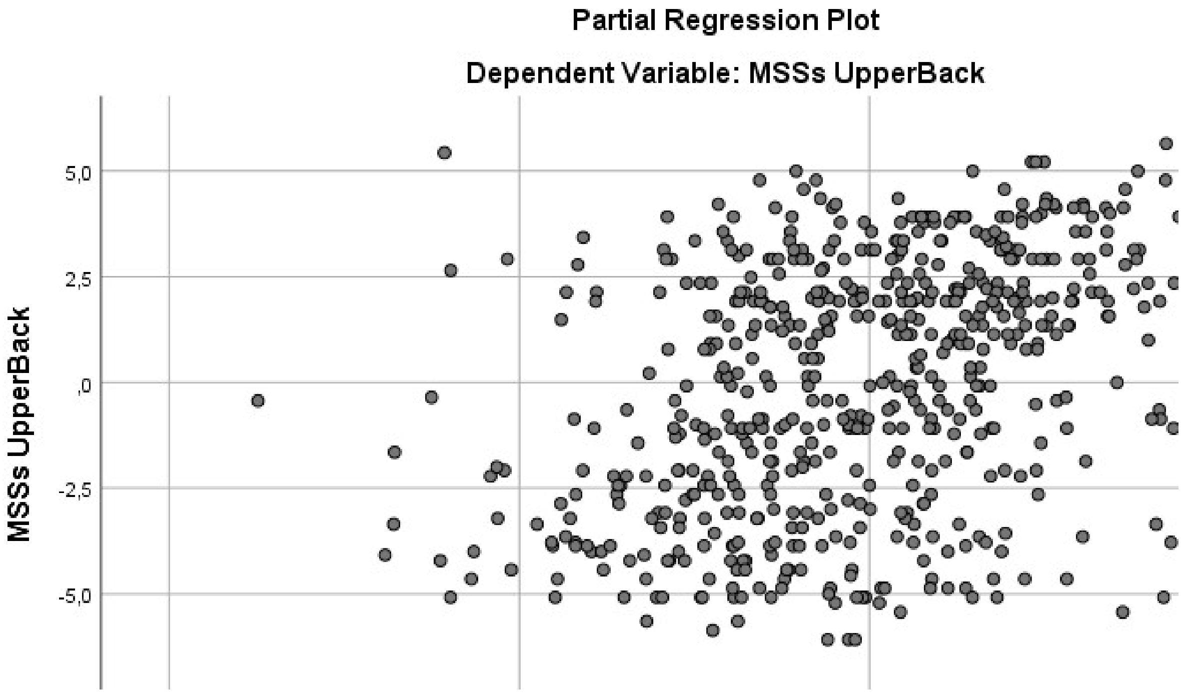
Partial Regression Plot
Dependent Variable: MSSs UpperBack
5,0
2,5
,0
-2,5
-5,0
MSSs UpperBack

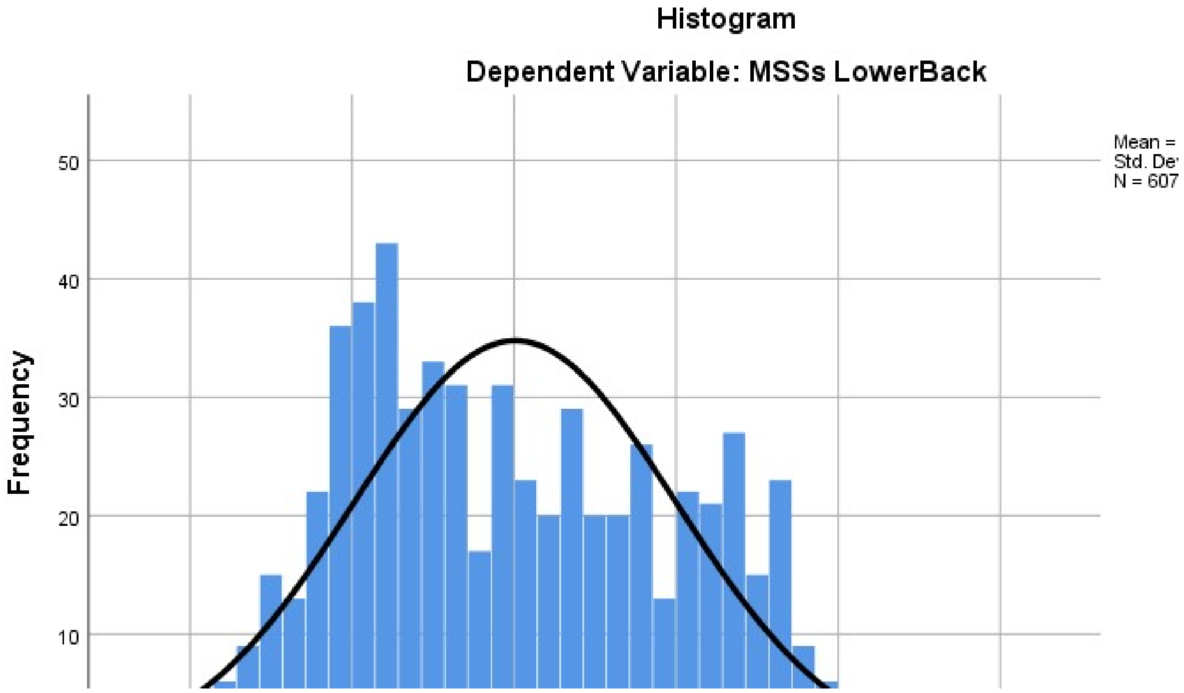
Histogram
Dependent Variable: MSSs LowerBack
Frequency
50
40
30
20
10
Mean =
Std. De
N = 607

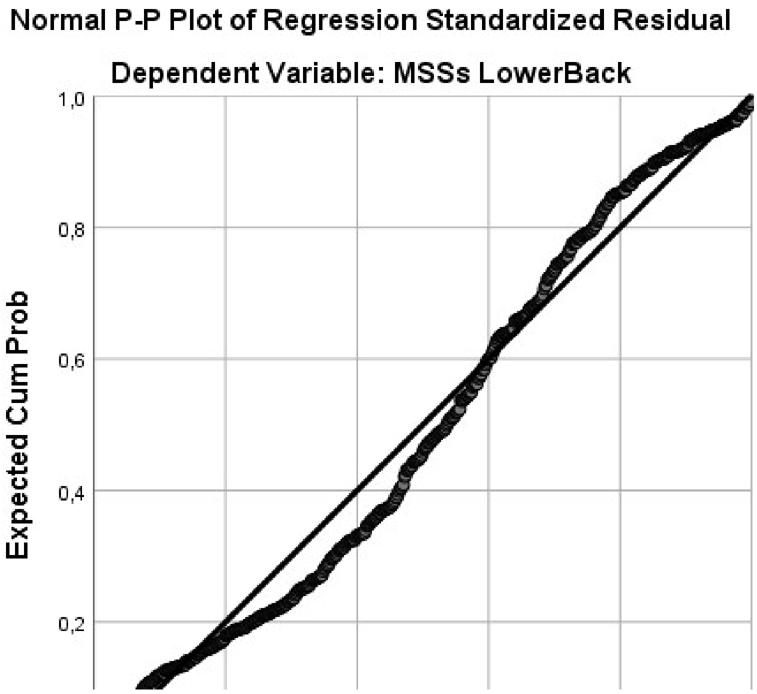
Normal P-P Plot of Regression Standardized Residual
Dependent Variable: MSSs LowerBack
Expected Cum Prob
1,0
0,8
0,6
0,4
0,2

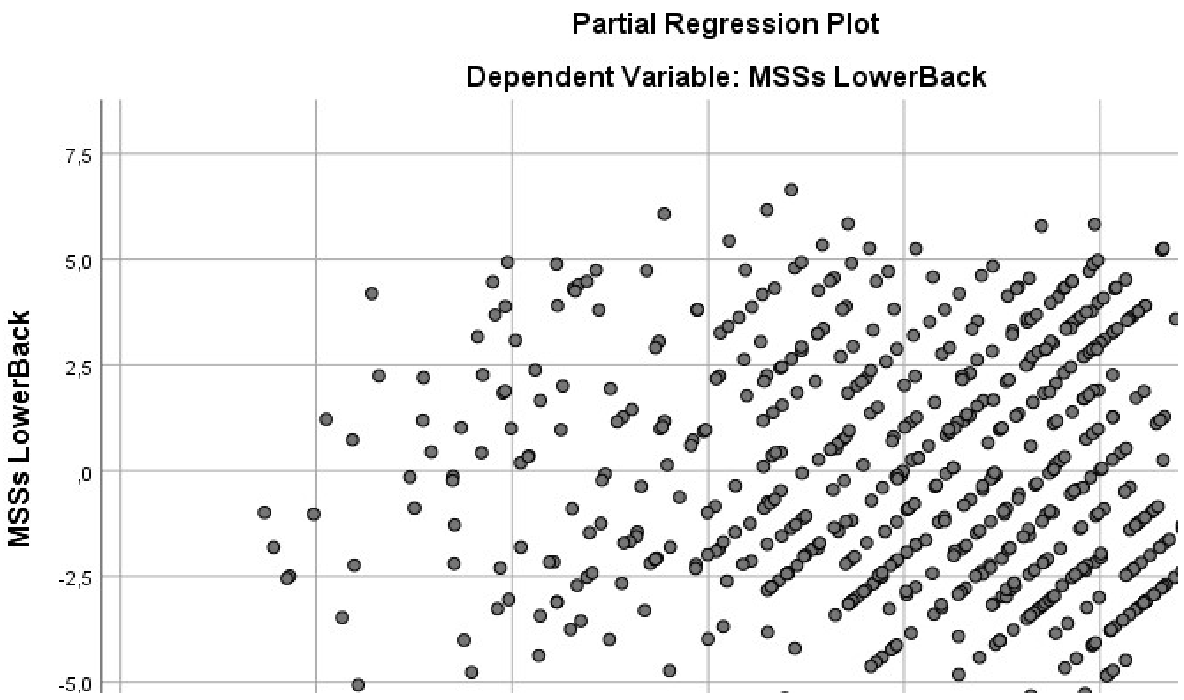
Partial Regression Plot
Dependent Variable: MSSs LowerBack
7,5
5,0
2,5
,0
-2,5
-5,0
MSSs LowerBack

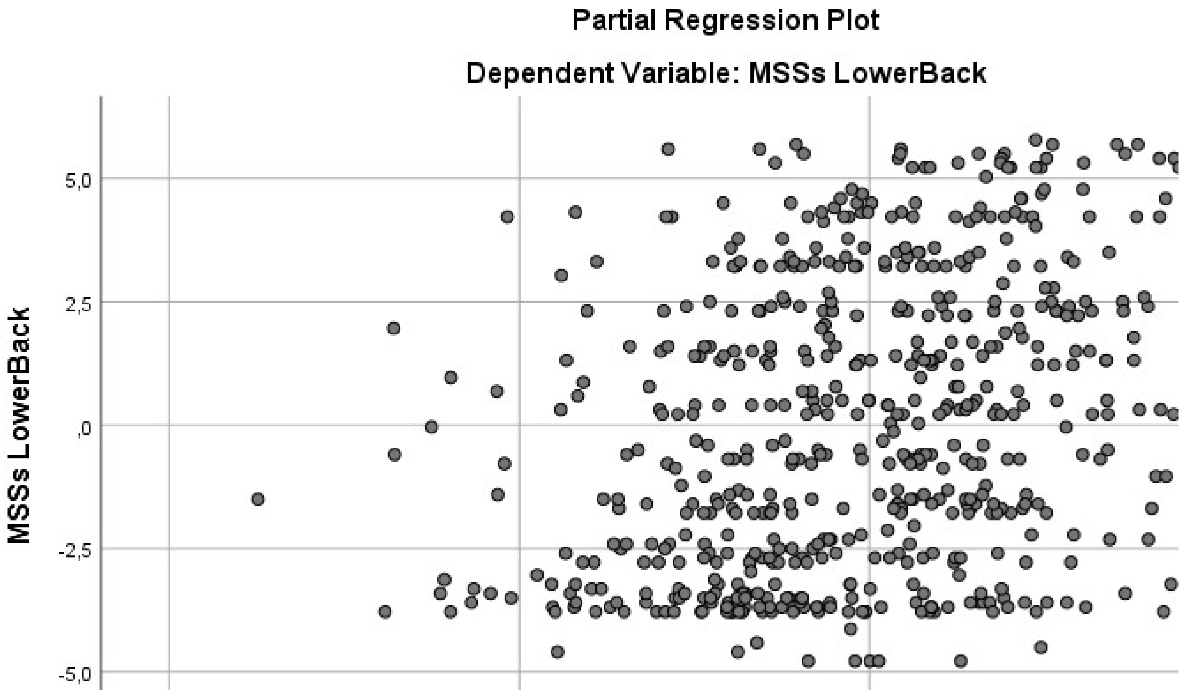
Partial Regression Plot
Dependent Variable: MSSs LowerBack
5,0
2,5
,0
-2,5
-5,0
MSSs LowerBack

Men’s Group

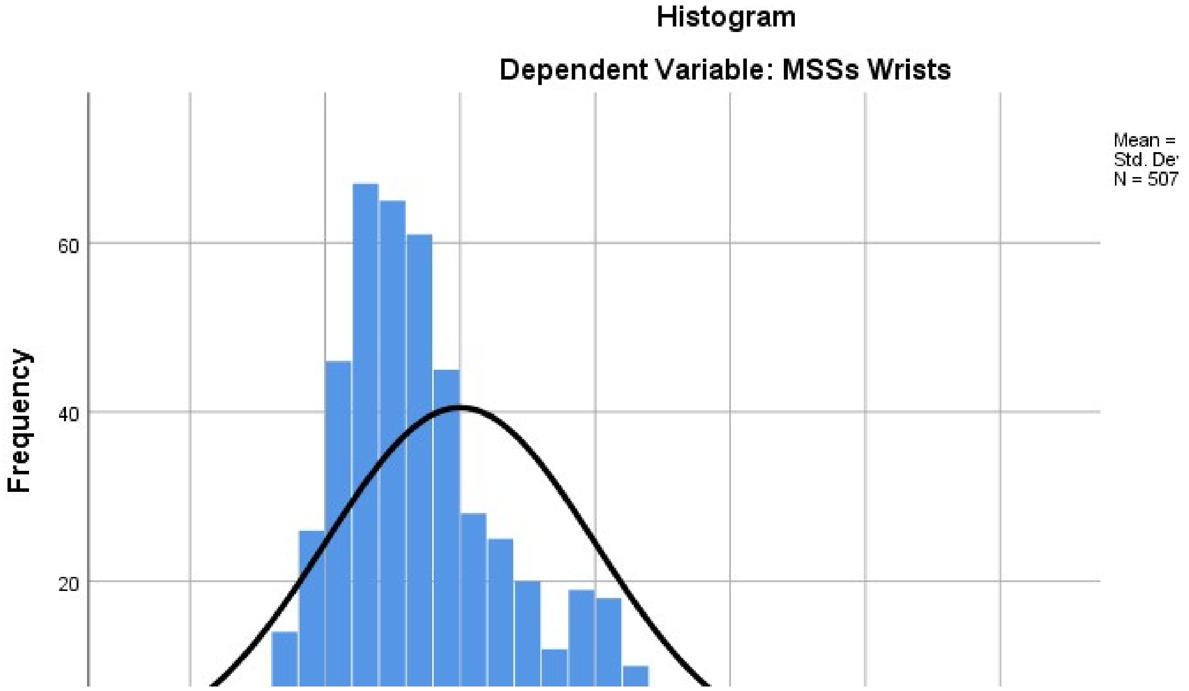

Histogram
Dependent Variable: MSSs Wrists
Mean =
Std. De
N = 507
Frequency
60
40
20

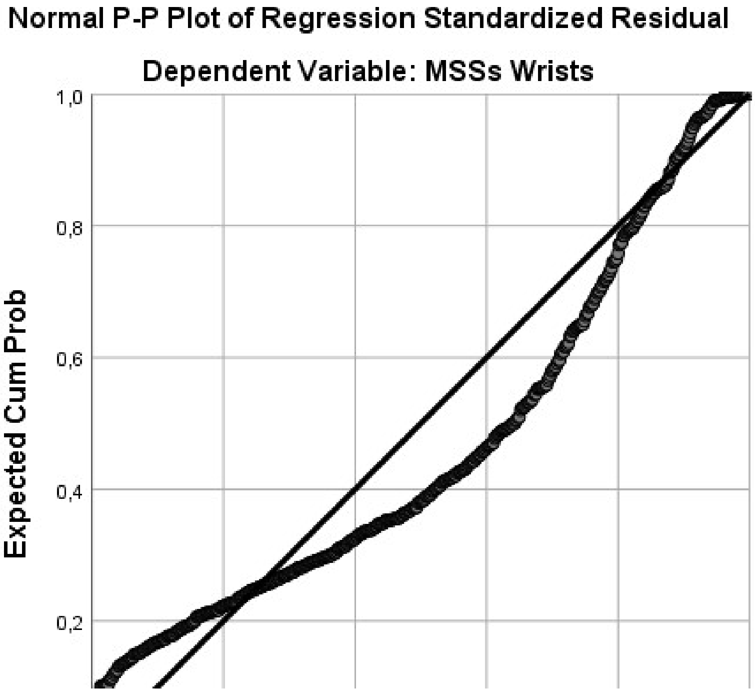

Normal P-P Plot of Regression Standardized Residual
Dependent Variable: MSSs Wrists
Expected Cum Prob
1,0
0,8
0,6
0,4
0,2

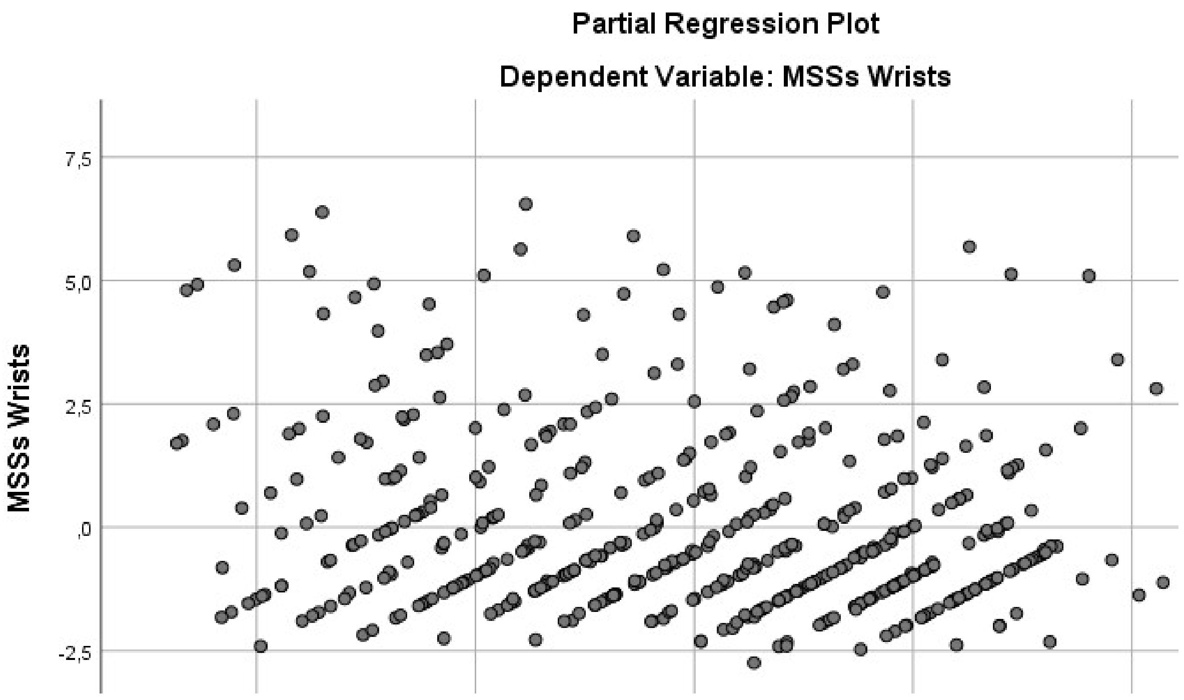
Partial Regression Plot
Dependent Variable: MSSs Wrists
MSSs Wrists
7,5
5,0
2,5
,0
-2,5

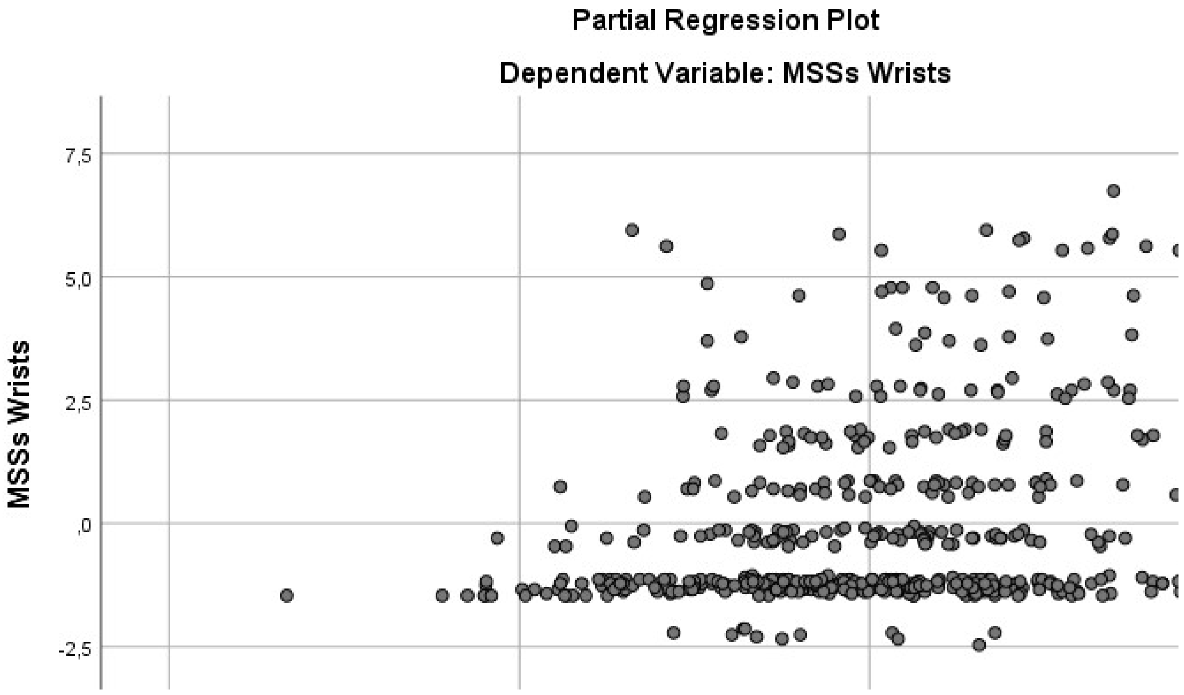
Partial Regression Plot
Dependent Variable: MSSs Wrists
MSSs Wrists
7,5
5,0
2,5
,0
-2,5

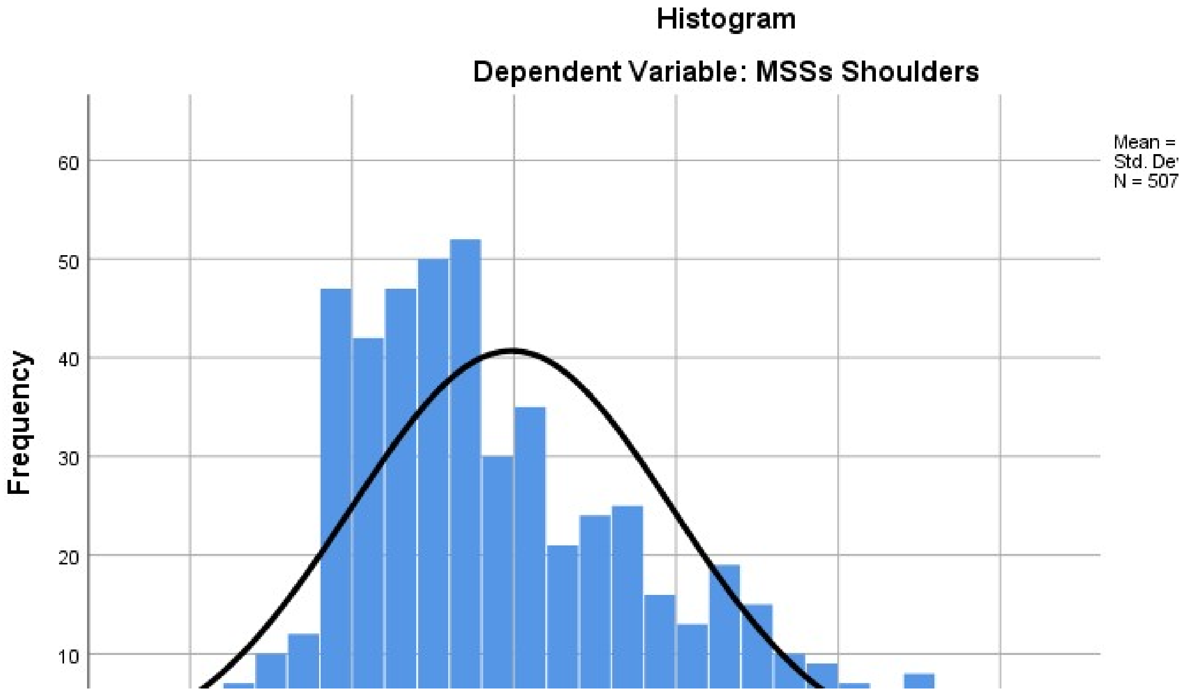

Histogram
Dependent Variable: MSSs Shoulders
Frequency
60
50
40
30
20
10
Mean =
Std. Dev
N = 507

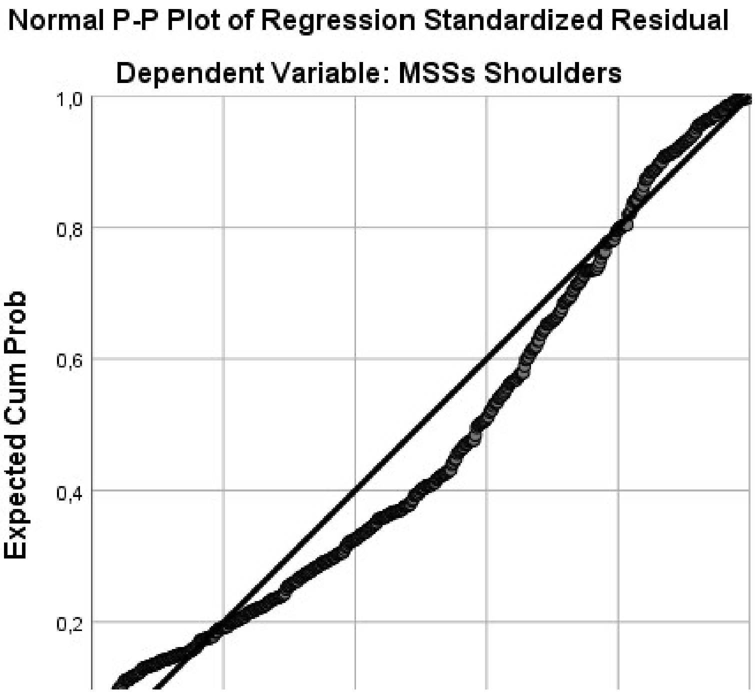

Normal P-P Plot of Regression Standardized Residual
Dependent Variable: MSSs Shoulders
Expected Cum Prob
1,0
0,8
0,6
0,4
0,2

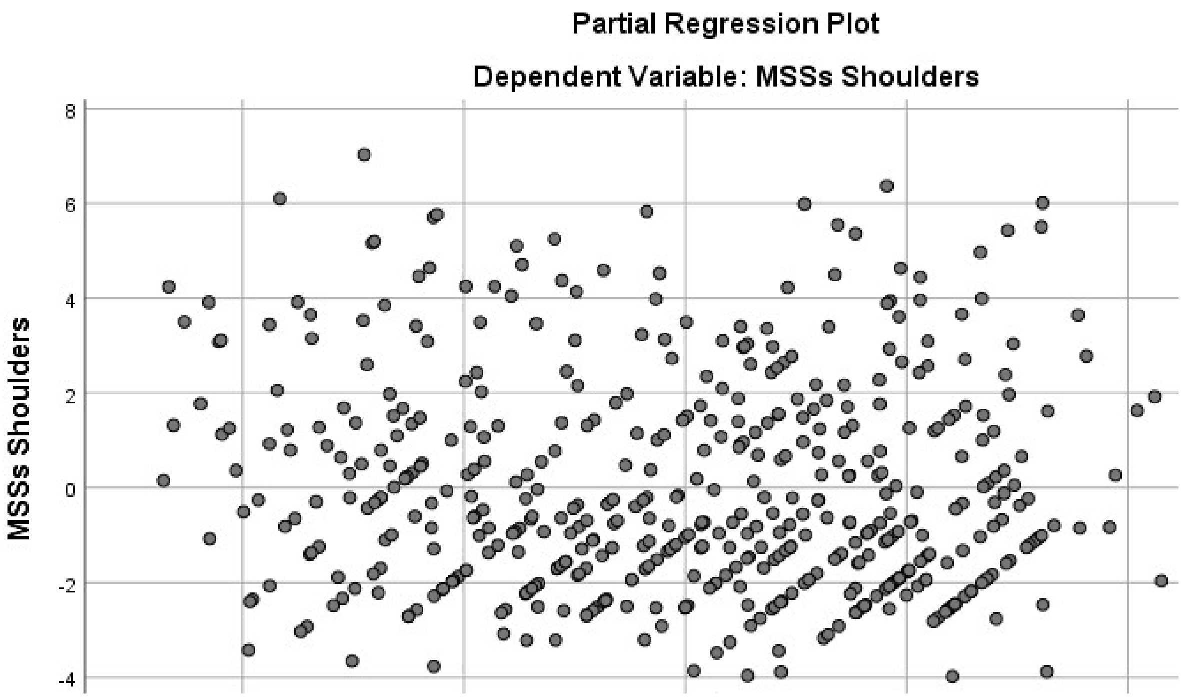
Partial Regression Plot
Dependent Variable: MSSs Shoulders
MSSs Shoulders
8
6
4
2
0
-2
-4

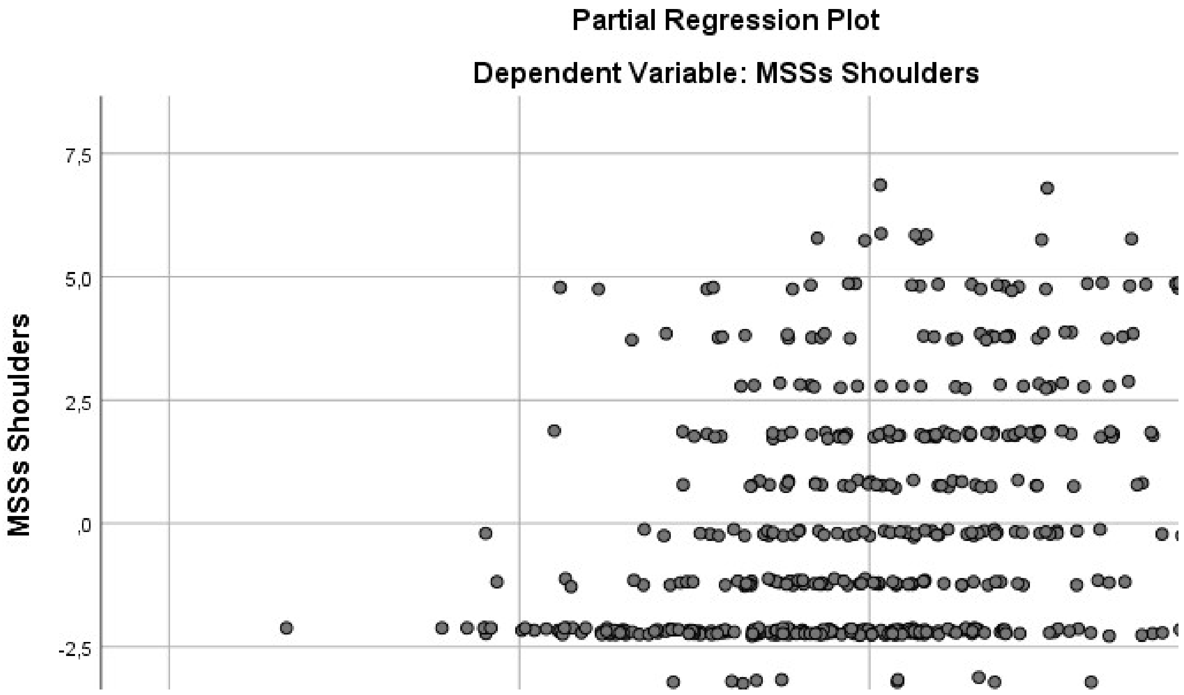
Partial Regression Plot
Dependent Variable: MSSs Shoulders
MSSs Shoulders
7,5
5,0
2,5
,0
-2,5

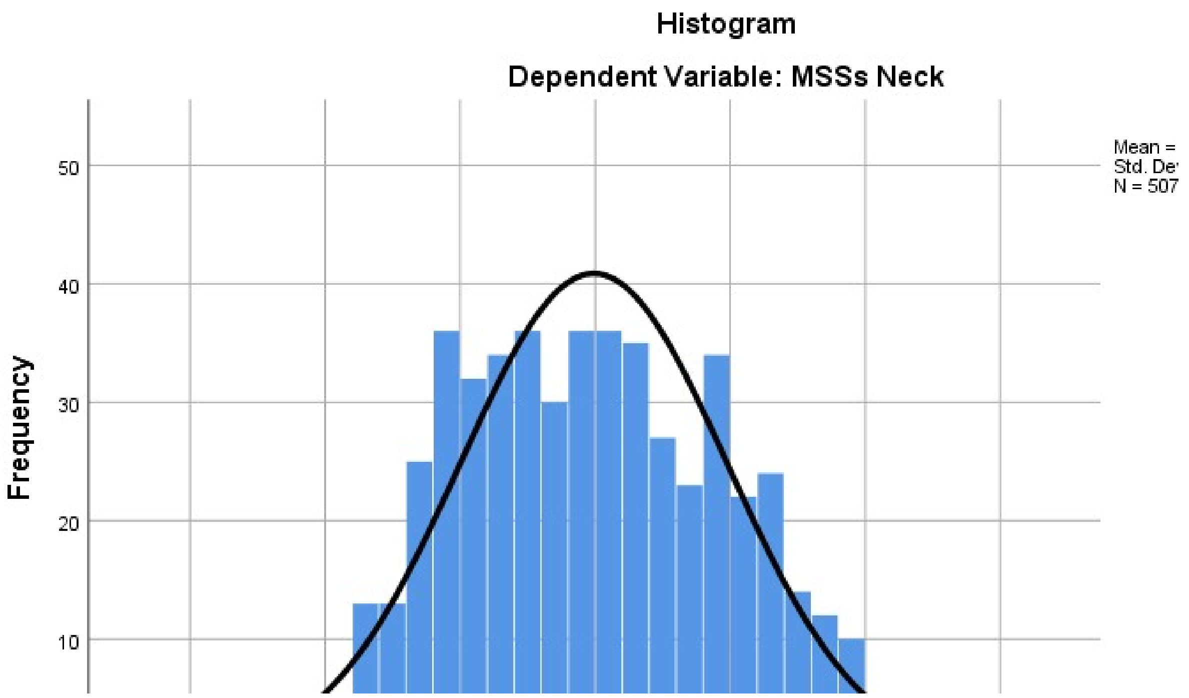
Histogram
Dependent Variable: MSSs Neck
Frequency
50
40
30
20
10
Mean =
Std. De
N = 507

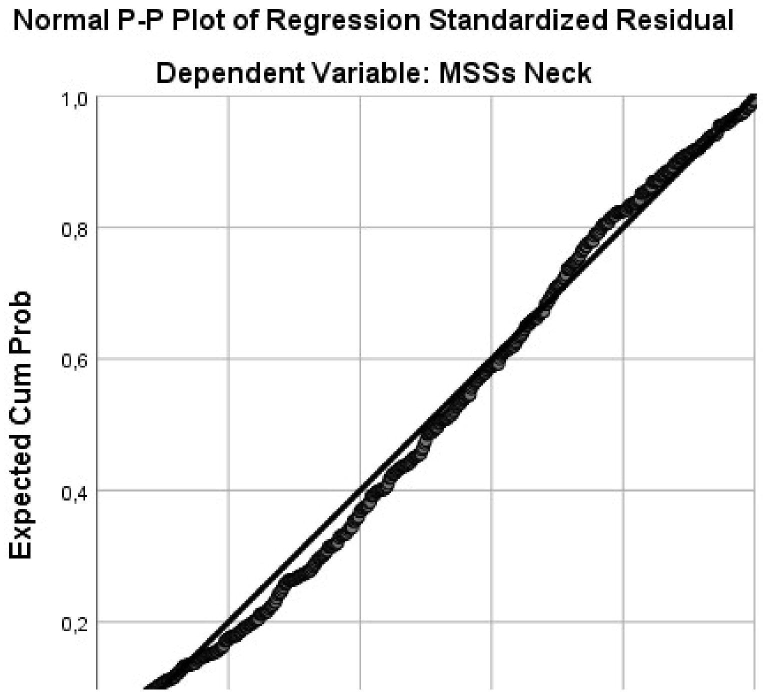
Normal P-P Plot of Regression Standardized Residual
Dependent Variable: MSSs Neck
Expected Cum Prob
1,0
0,8
0,6
0,4
0,2

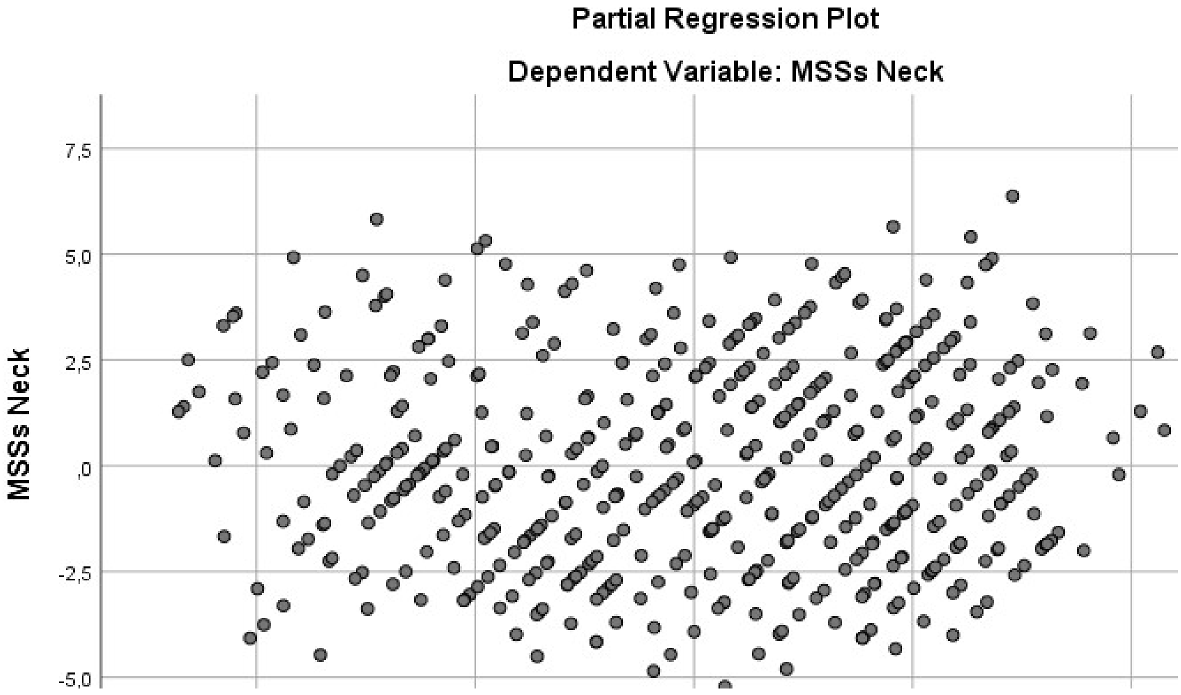
Partial Regression Plot
Dependent Variable: MSSs Neck
7,5
5,0
2,5
,0
-2,5
-5,0
MSSs Neck

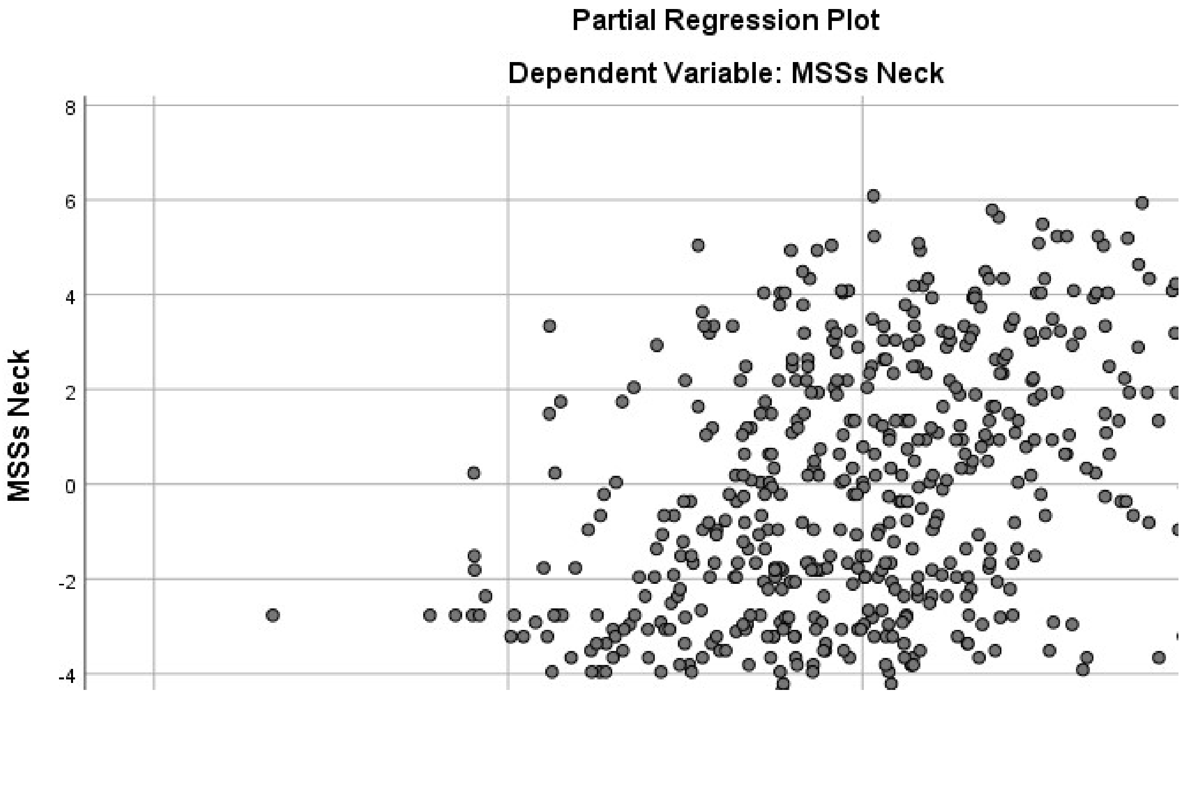
Partial Regression Plot
Dependent Variable: MSSs Neck
8
6
4
2
0
-2
-4
MSSs Neck

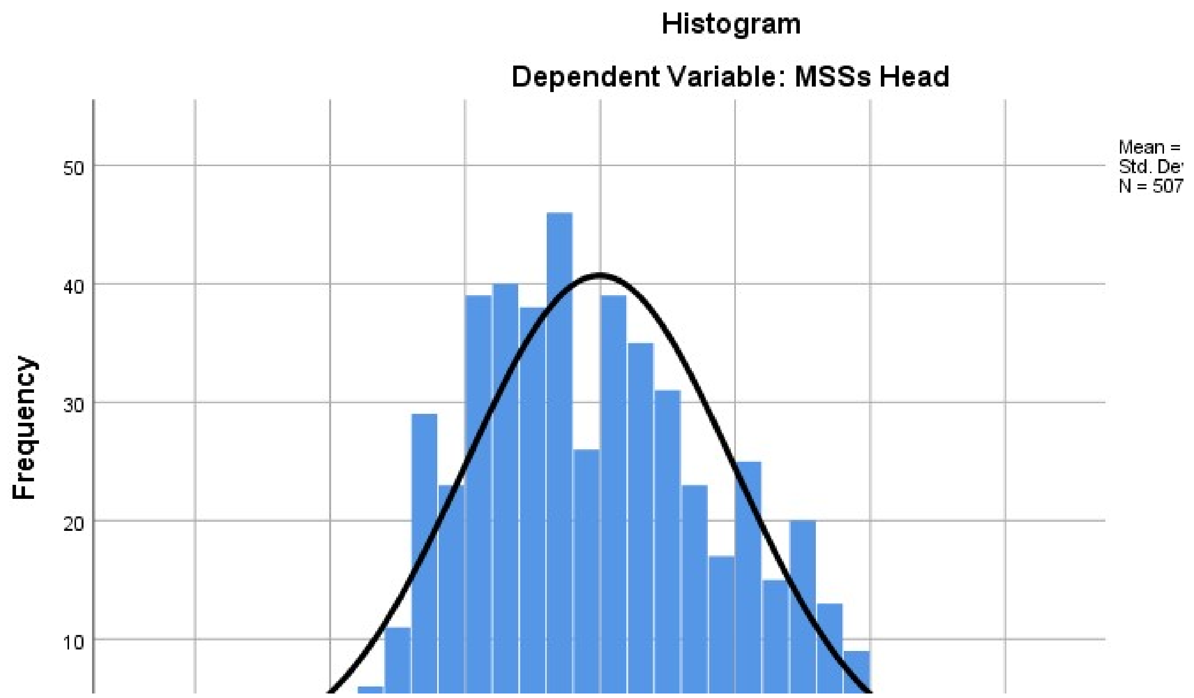
Histogram
Dependent Variable: MSSs Head
Frequency
50
40
30
20
10
Mean =
Std. De
N = 507

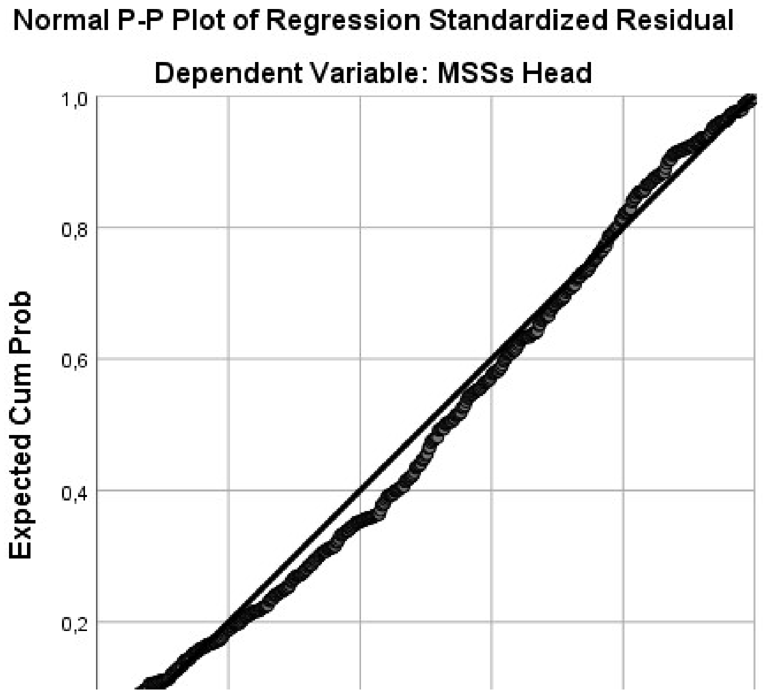
Normal P-P Plot of Regression Standardized Residual
Dependent Variable: MSSs Head
Expected Cum Prob
1,0
0,8
0,6
0,4
0,2

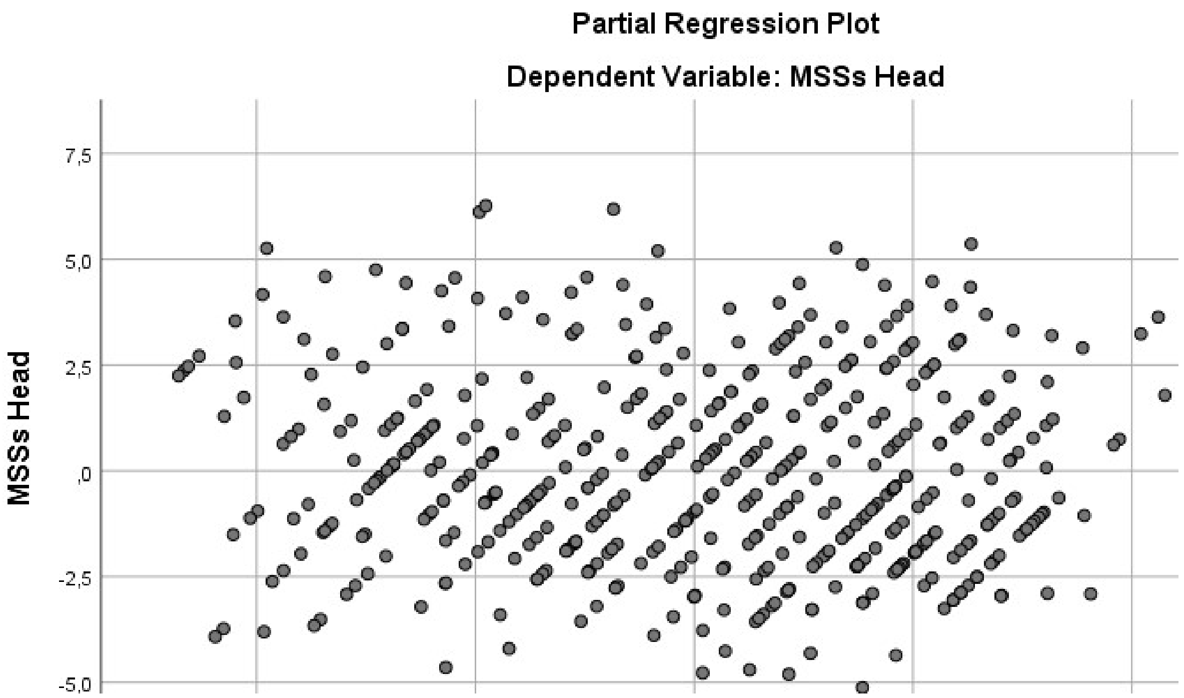
Partial Regression Plot
Dependent Variable: MSSs Head
MSSs Head
7,5
5,0
2,5
,0
-2,5
-5,0

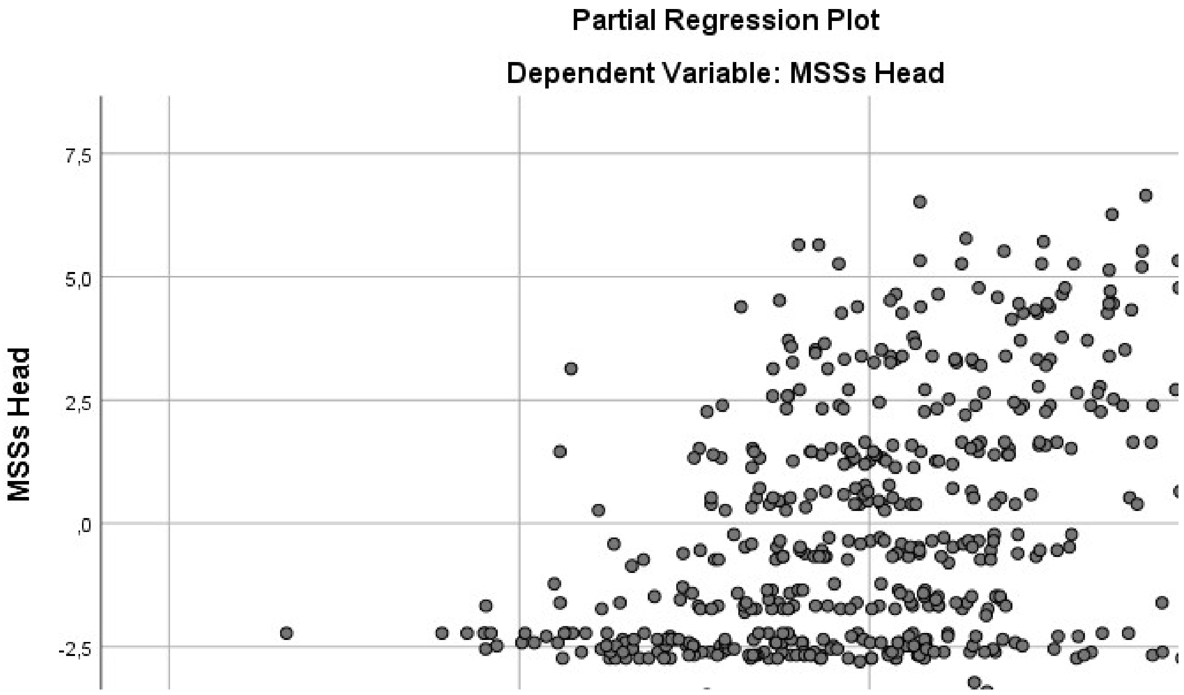
Partial Regression Plot
Dependent Variable: MSSs Head
MSSs Head
7,5
5,0
2,5
,0
-2,5

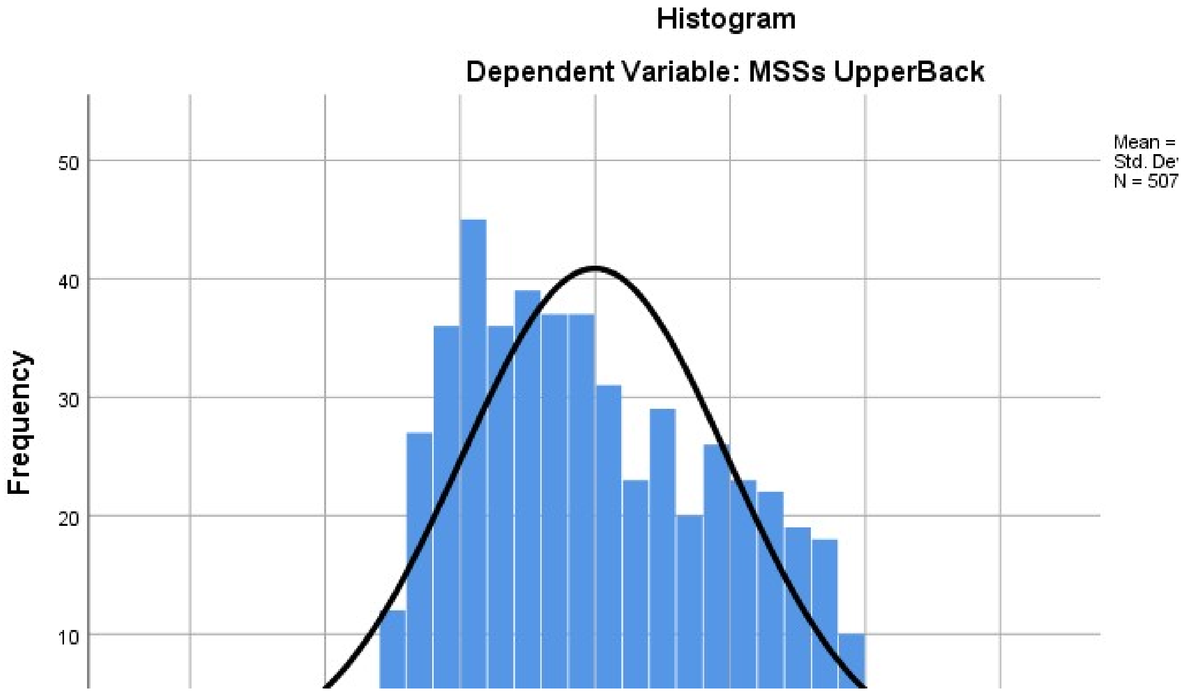
Histogram
Dependent Variable: MSSs UpperBack
Frequency
50
40
30
20
10
Mean =
Std. De
N = 507

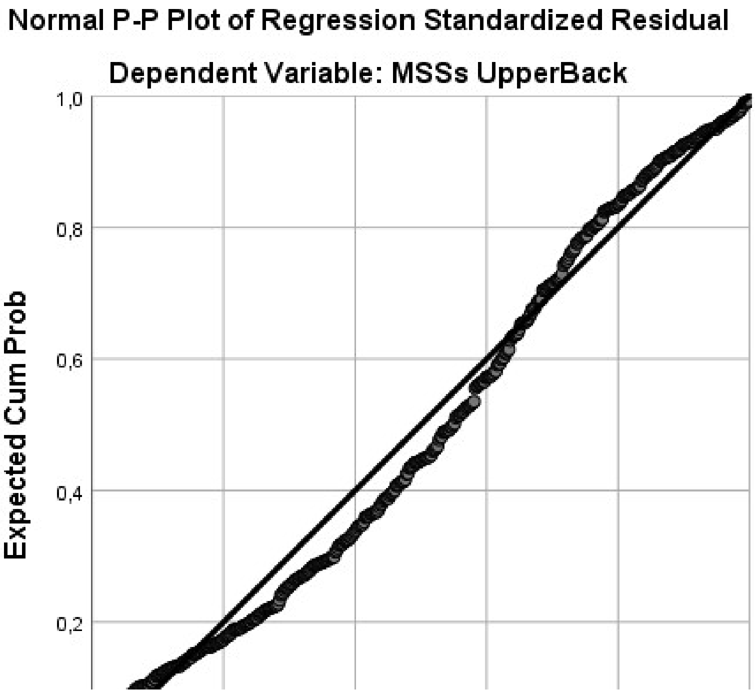
Normal P-P Plot of Regression Standardized Residual
Dependent Variable: MSSs UpperBack
Expected Cum Prob
1,0
0,8
0,6
0,4
0,2

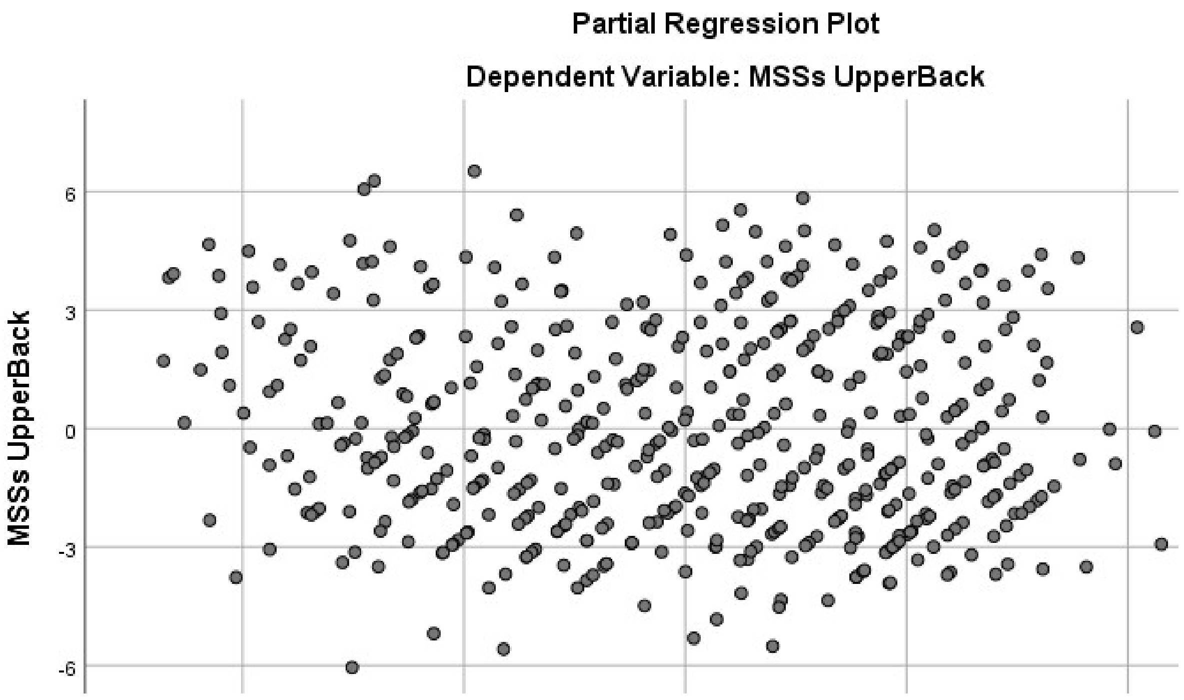
Partial Regression Plot
Dependent Variable: MSSs UpperBack
MSSs UpperBack
6
3
0
-3
-6

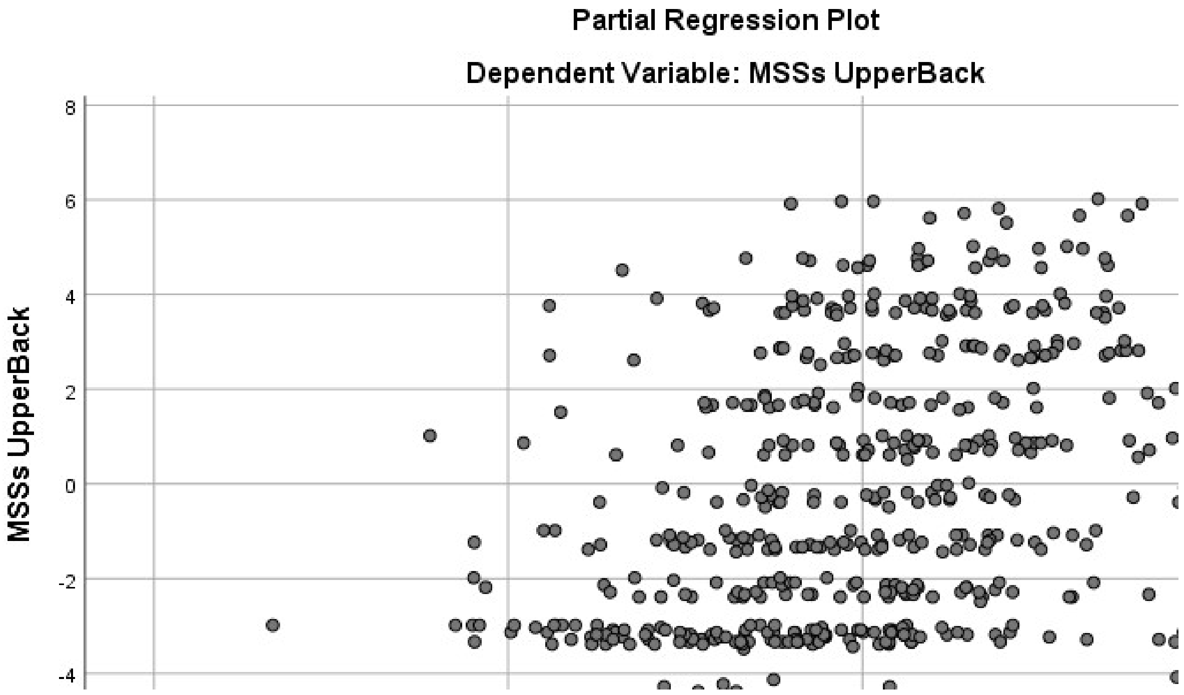
Partial Regression Plot
Dependent Variable: MSSs UpperBack
MSSs UpperBack
8
6
4
2
0
-2
-4

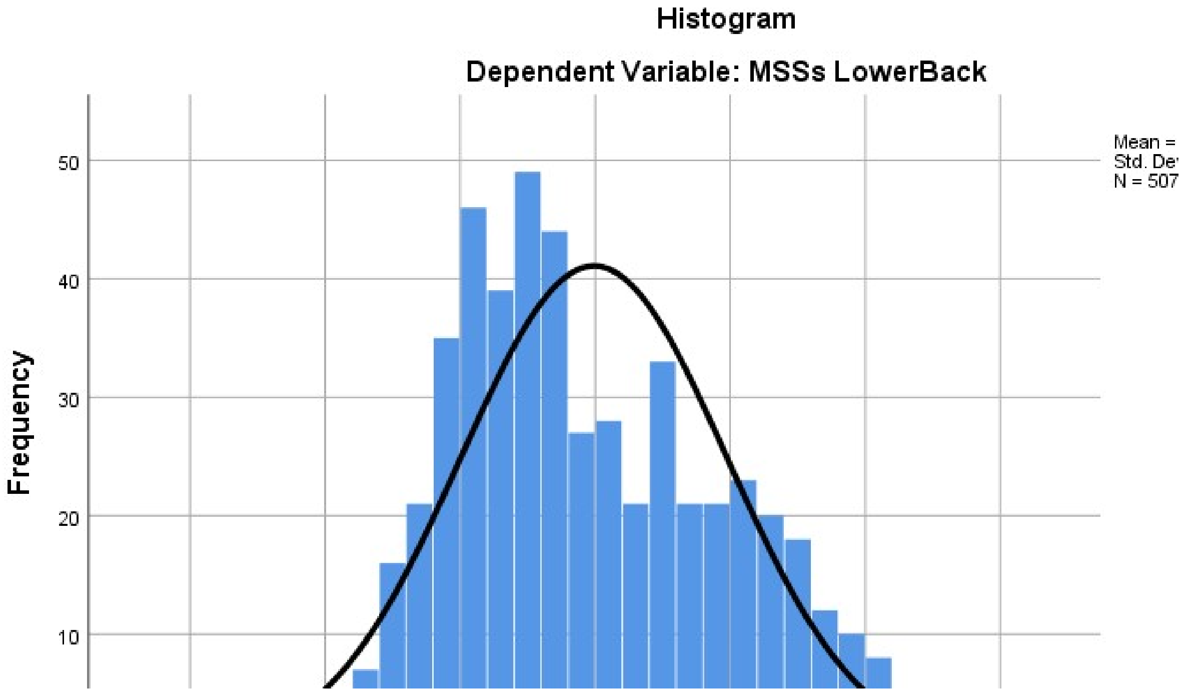
Histogram
Dependent Variable: MSSs LowerBack
Frequency
50
40
30
20
10
Mean =
Std. De
N = 507

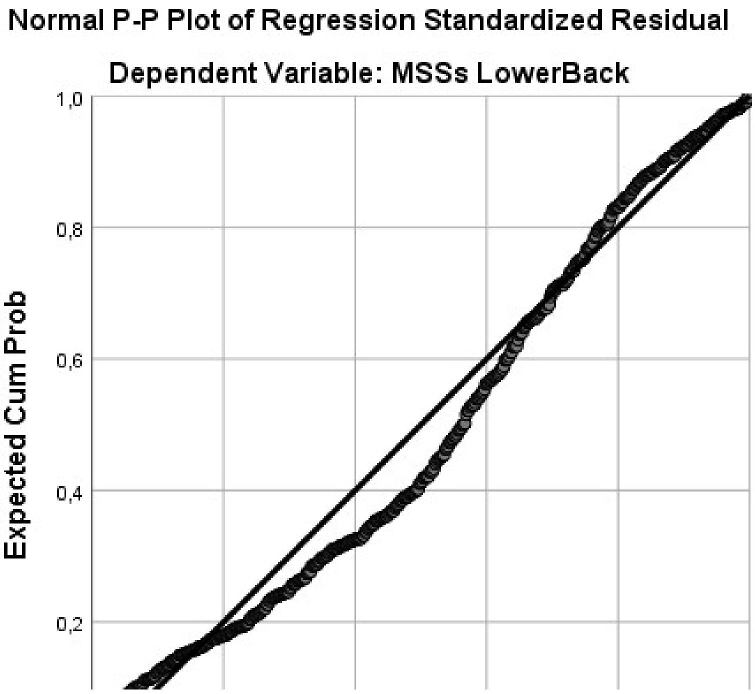
Normal P-P Plot of Regression Standardized Residual
Dependent Variable: MSSs LowerBack
Expected Cum Prob
1,0
0,8
0,6
0,4
0,2

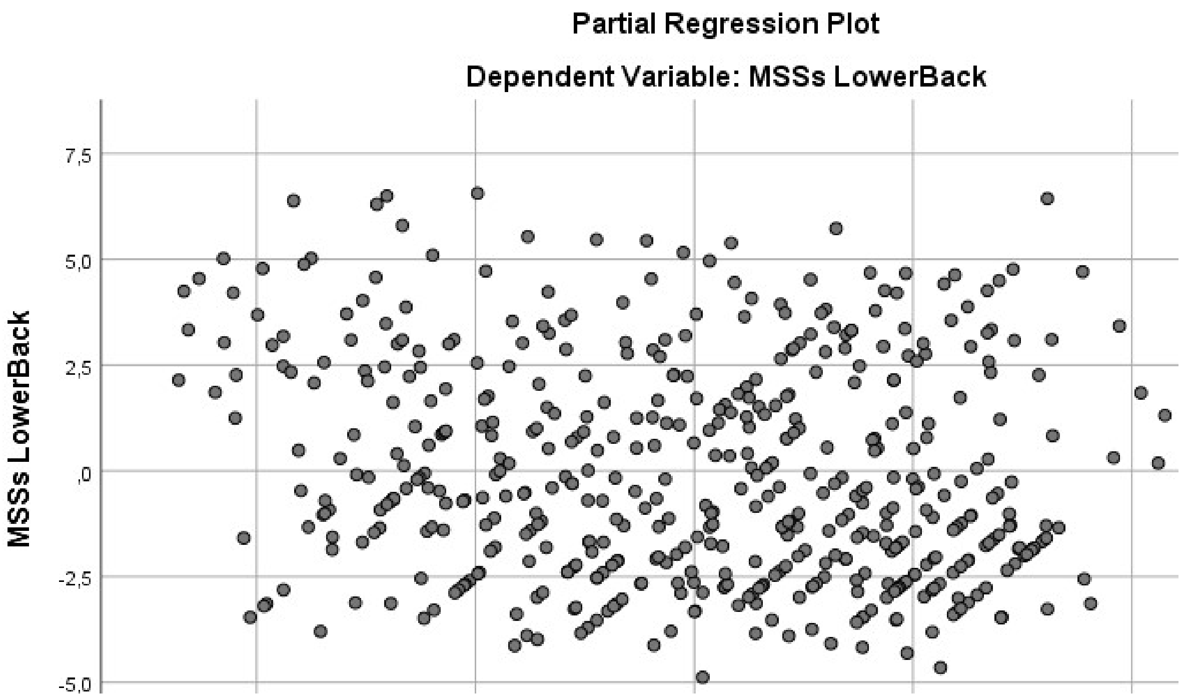
Partial Regression Plot
Dependent Variable: MSSs LowerBack
MSSs LowerBack
7,5
5,0
2,5
,0
-2,5
-5,0

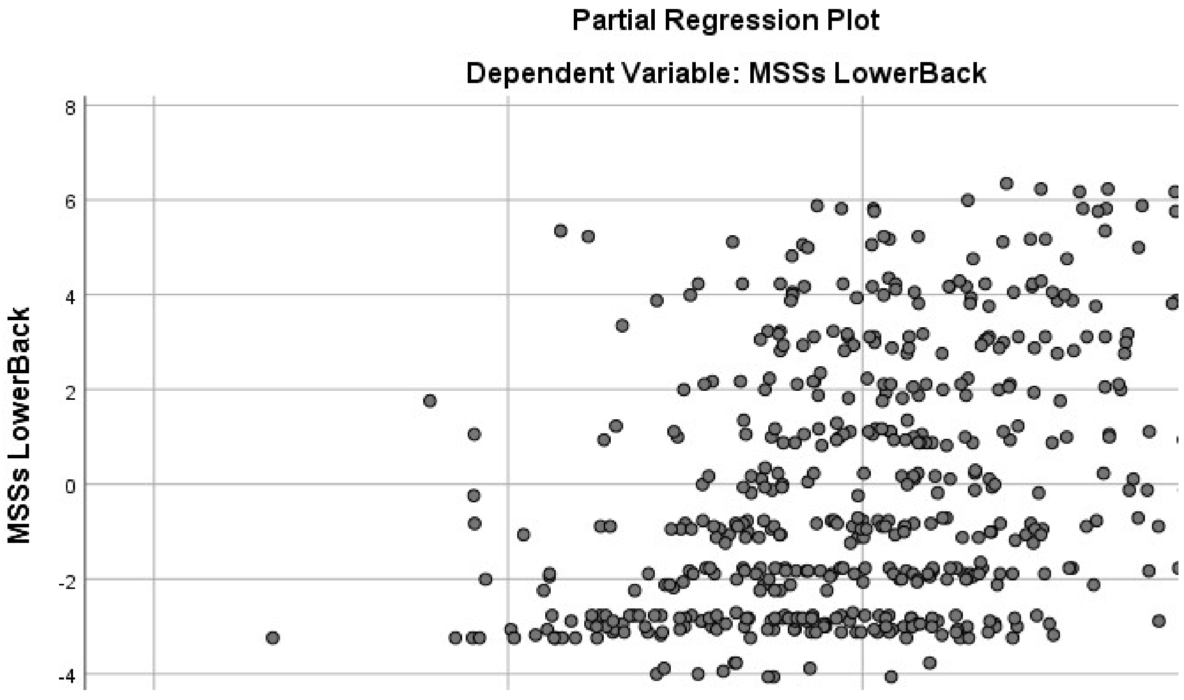
Partial Regression Plot
Dependent Variable: MSSs LowerBack
MSSs LowerBack
8
6
4
2
0
-2
-4
