# Supplementary material for: Do psychosocial factors mediate the appearance of musculoskeletal symptoms? Evidence of an empirical study about the role of mental workload in computer workers
Source: PLoS One. 2021 Jun 17;16(6):e0252179. doi: 10.1371/journal.pone.0252179 (PMC8211229; doi:10.1371/journal.pone.0252179)
Supplement: S2 File — (PDF) [file pone.0252179.s002.pdf]

# STRUCTURAL EQUATION MODELING. GLOBAL RESULTS

# IBM®- AMOS® 24.0 OUTPUT

**(NOTE:** Results for both men and women are exposed in the following output, though only MSD-Neck for men, MSD-Neck, MSD-Shoulders and MSD-Upper back models are exposed in the published article)

# INDEX


## 1. MSD NECK: Page 1 to Page 16

## 2. MSD SHOULDERS: Page 17 to Page 31

## 3. MSD UPPER BACK: Page 32 to 46


## 1. MSD NECK

**Groups**

**Group number 1 (Group number 1= Men )**
**Notes for Group (Group number 1= Men )**

The model is recursive.
Sample size = 527

**Variable Summary (Men)**
**Your model contains the following variables (Men)**

Observed, endogenous variables
    MSD Neck
    MentalWLFac
Observed, exogenous variables
    Age
    Adequate furniture

Adequate ICT devices
Ratio Pauses Hour
Daily time Computer use
Intense Physical activity

Unobserved, exogenous variables

eMSD
eMWL

**Variable counts (Men)**

| | |
|---|---|
| **Number of variables in your model:** | 10 |
| **Number of observed variables:** | 8 |
| **Number of unobserved variables:** | 2 |
| **Number of exogenous variables:** | 8 |
| **Number of endogenous variables:** | 2 |

**Parameter Summary (Men)**

| | Weights | Covariances | Variances | Means | Intercepts | Total |
|---|---|---|---|---|---|---|
| **Fixed** | 2 | 0 | 0 | 2 | 0 | 4 |
| **Labeled** | 0 | 0 | 0 | 0 | 0 | 0 |
| **Unlabeled** | 13 | 15 | 8 | 6 | 2 | 44 |
| **Total** | 15 | 15 | 8 | 8 | 2 | 48 |

**Group number 2 (Group number 2= Women )**
**Notes for Group (Group number 2= Women )**

The model is recursive.
Sample size = 671

**Variable Summary (Women)**
**Your model contains the following variables (Women)**

Observed, endogenous variables

MSD Neck
MentalWLFac

Observed, exogenous variables

Age
Adequate furniture
Adequate ICT devices
Ratio Pauses Hour
Daily time Computer use
Intense Physical activity

Unobserved, exogenous variables

eMSD

eMWL

**Variable counts (Women)**

| | |
|---|---|
| **Number of variables in your model:** | 10 |
| **Number of observed variables:** | 8 |
| **Number of unobserved variables:** | 2 |
| **Number of exogenous variables:** | 8 |
| **Number of endogenous variables:** | 2 |

**Parameter Summary (Women)**

| | Weights | Covariances | Variances | Means | Intercepts | Total |
|---|---|---|---|---|---|---|
| **Fixed** | 2 | 0 | 0 | 2 | 0 | 4 |
| **Labeled** | 0 | 0 | 0 | 0 | 0 | 0 |
| **Unlabeled** | 13 | 15 | 8 | 6 | 2 | 44 |
| **Total** | 15 | 15 | 8 | 8 | 2 | 48 |

**Models**
**Default model (Default model)**
**Notes for Model (Default model)**
**Computation of degrees of freedom (Default model)**

| | |
|---|---|
| **Number of distinct sample moments:** | 88 |
| **Number of distinct parameters to be estimated:** | 88 |
| **Degrees of freedom (88 - 88):** | 0 |

**Result (Default model)**

Minimum was achieved
Chi-square = ,000
Degrees of freedom = 0
Probability level cannot be computed

**Men (Men - Default model)**
**Estimates (Men - Default model)**
**Scalar Estimates (Men - Default model)**
**Maximum Likelihood Estimates**
**Regression Weights: (Men - Default model)**

| | | | Estimate | S.E. | C.R. | P | Label |
|---|---|---|---|---|---|---|---|
| MentalWLFac | <--- | Age | -,008 | ,005 | -1,620 | ,105 | par_2 |
| MentalWLFac | <--- | Adequate furniture | -,109 | ,097 | -1,122 | ,262 | par_4 |

| | | | Estimate | S.E. | C.R. | P | Label |
|---|---|---|---|---|---|---|---|
| MentalWLFac | <--- | Adequate ICT devices | -,162 | ,094 | -1,710 | ,087 | par_5 |
| MentalWLFac | <--- | Daily time Computer use | ,076 | ,018 | 4,320 | *** | par_10 |
| MentalWLFac | <--- | Ratio Pauses Hour | -,049 | ,055 | -,900 | ,368 | par_11 |
| MentalWLFac | <--- | Intense Physical activity | ,018 | ,014 | 1,332 | ,183 | par_27 |
| MSD Neck | <--- | MentalWLFac | 1,203 | ,118 | 10,192 | *** | par_1 |
| MSD Neck | <--- | Age | -,009 | ,012 | -,728 | ,467 | par_3 |
| MSD Neck | <--- | Ratio Pauses Hour | ,099 | ,131 | ,754 | ,451 | par_6 |
| MSD Neck | <--- | Daily time Computer use | ,080 | ,046 | 1,726 | ,084 | par_7 |
| MSD Neck | <--- | Adequate furniture | -,138 | ,256 | -,540 | ,589 | par_8 |
| MSD Neck | <--- | Adequate ICT devices | -,464 | ,249 | -1,865 | ,062 | par_9 |
| MSD Neck | <--- | Intense Physical activity | ,063 | ,035 | 1,787 | ,074 | par_28 |

**Standardized Regression Weights: (Men - Default model)**

| | | | Estimate |
|---|---|---|---|
| MentalWLFac | <--- | Age | -,070 |
| MentalWLFac | <--- | Adequate furniture | -,054 |
| MentalWLFac | <--- | Adequate ICT devices | -,082 |
| MentalWLFac | <--- | Daily time Computer use | ,214 |
| MentalWLFac | <--- | Ratio Pauses Hour | -,048 |
| MentalWLFac | <--- | Intense Physical activity | ,058 |
| MSD Neck | <--- | MentalWLFac | ,417 |
| MSD Neck | <--- | Age | -,029 |
| MSD Neck | <--- | Ratio Pauses Hour | ,034 |
| MSD Neck | <--- | Daily time Computer use | ,077 |
| MSD Neck | <--- | Adequate furniture | -,024 |
| MSD Neck | <--- | Adequate ICT devices | -,082 |
| MSD Neck | <--- | Intense Physical activity | ,070 |

**Means: (Men - Default model)**

| | Estimate | S.E. | C.R. | P | Label |
|---|---|---|---|---|---|
| **Daily time Computer use** | 4,780 | ,116 | 41,033 | *** | par_30 |
| **Ratio Pauses Hour** | ,859 | ,043 | 19,892 | *** | par_32 |
| **Age** | 45,004 | ,383 | 117,515 | *** | par_33 |
| **Adequate ICT devices** | ,626 | ,021 | 29,589 | *** | par_34 |
| **Adequate furniture** | ,656 | ,021 | 31,617 | *** | par_35 |
| **Intense Physical activity** | 4,304 | ,133 | 32,348 | *** | par_36 |

**Intercepts: (Men - Default model)**

| | Estimate | S.E. | C.R. | P | Label |
|---|---|---|---|---|---|
| **MentalWLFac** | ,111 | ,265 | ,420 | ,675 | par_29 |
| **MSD Neck** | 4,530 | ,682 | 6,641 | *** | par_31 |

**Covariances: (Men - Default model)**

| | | | Estimate | S.E. | C.R. | P | Label |
|---|---|---|---|---|---|---|---|
| Adequate furniture | <--> | Adequate ICT devices | ,102 | ,011 | 9,313 | *** | par_12 |
| Adequate ICT devices | <--> | Daily time Computer use | ,053 | ,057 | ,930 | ,352 | par_13 |
| Adequate furniture | <--> | Daily time Computer use | ,230 | ,056 | 4,088 | *** | par_14 |
| Adequate furniture | <--> | Ratio Pauses Hour | -,045 | ,021 | -2,153 | ,031 | par_15 |
| Ratio Pauses Hour | <--> | Daily time Computer use | -1,095 | ,137 | -8,012 | *** | par_16 |
| Age | <--> | Ratio Pauses Hour | ,085 | ,371 | ,230 | ,818 | par_17 |
| Ratio Pauses Hour | <--> | Intense Physical activity | ,057 | ,130 | ,442 | ,658 | par_18 |
| Age | <--> | Daily time Computer use | -,094 | 1,023 | -,092 | ,927 | par_19 |
| Age | <--> | Adequate furniture | -,009 | ,182 | -,048 | ,962 | par_20 |
| Age | <--> | Adequate ICT devices | -,326 | ,186 | -1,754 | ,079 | par_21 |
| Age | <--> | Intense Physical activity | -2,857 | 1,175 | -2,431 | ,015 | par_22 |
| Daily time Computer use | <--> | Intense Physical activity | -,233 | ,356 | -,655 | ,512 | par_23 |
| Adequate furniture | <--> | Intense Physical activity | ,011 | ,063 | ,166 | ,868 | par_24 |
| Adequate ICT devices | <--> | Intense Physical activity | ,093 | ,065 | 1,438 | ,151 | par_25 |
| Adequate ICT devices | <--> | Ratio Pauses Hour | -,001 | ,021 | -,035 | ,972 | par_26 |

**Correlations: (Men - Default model)**

| | | | Estimate |
|---|---|---|---|
| Adequate furniture | <--> | Adequate ICT devices | ,446 |
| Adequate ICT devices | <--> | Daily time Computer use | ,041 |
| Adequate furniture | <--> | Daily time Computer use | ,182 |
| Adequate furniture | <--> | Ratio Pauses Hour | -,101 |
| Ratio Pauses Hour | <--> | Daily time Computer use | -,437 |
| Age | <--> | Ratio Pauses Hour | ,010 |
| Ratio Pauses Hour | <--> | Intense Physical activity | ,020 |
| Age | <--> | Daily time Computer use | -,004 |
| Age | <--> | Adequate furniture | -,002 |
| Age | <--> | Adequate ICT devices | -,077 |
| Age | <--> | Intense Physical activity | -,107 |
| Daily time Computer use | <--> | Intense Physical activity | -,029 |
| Adequate furniture | <--> | Intense Physical activity | ,007 |

| | | | Estimate |
|---|---|---|---|
| Adequate ICT devices | <--> | Intense Physical activity | ,063 |
| Adequate ICT devices | <--> | Ratio Pauses Hour | -,002 |

**Variances: (Men - Default model)**

| | Estimate | S.E. | C.R. | P | Label |
|---|---|---|---|---|---|
| **Age** | 77,161 | 4,757 | 16,219 | *** | par_73 |
| **Adequate furniture** | ,225 | ,014 | 16,176 | *** | par_74 |
| **Adequate ICT devices** | ,234 | ,014 | 16,175 | *** | par_75 |
| **Ratio Pauses Hour** | ,879 | ,060 | 14,703 | *** | par_76 |
| **Daily time Computer use** | 7,139 | ,440 | 16,219 | *** | par_77 |
| **Intense Physical activity** | 9,312 | ,574 | 16,219 | *** | par_78 |
| **eMWL** | ,843 | ,053 | 15,867 | *** | par_79 |
| **eMSD** | 5,962 | ,371 | 16,078 | *** | par_80 |

**Squared Multiple Correlations: (Men - Default model)**

| | Estimate |
|---|---|
| **MentalWLFac** | ,071 |
| **MSD Neck** | ,213 |

**Matrices (Men - Default model)**
**Implied (for all variables) Covariances (Men - Default model)**

| | Intense Physical activity | Daily time Computer use | Ratio Pauses Hour | Adequate ICT devices | Adequate furniture | Age | MentalWLFac | MSD Neck |
|---|---|---|---|---|---|---|---|---|
| **Intense Physical activity** | 9,312 | | | | | | | |
| **Daily time Computer use** | -,233 | 7,139 | | | | | | |
| **Ratio Pauses Hour** | ,057 | -1,095 | ,879 | | | | | |
| **Adequate ICT devices** | ,093 | ,053 | -,001 | ,234 | | | | |
| **Adequate furniture** | ,011 | ,230 | -,045 | ,102 | ,225 | | | |
| **Age** | -2,857 | -,094 | ,085 | -,326 | -,009 | 77,161 | | |
| **MentalWLFac** | ,152 | ,561 | -,121 | -,041 | -,021 | -,594 | ,908 | |
| **MSD Neck** | ,740 | 1,066 | -,137 | -,159 | -,089 | -1,433 | 1,162 | 7,578 |

**Implied (for all variables) Correlations (Men - Default model)**

| | Intense Physical activity | Daily time Computer use | Ratio Pauses Hour | Adequate ICT devices | Adequate furniture | Age | MentalWLFac | MSD Neck |
|---|---|---|---|---|---|---|---|---|
| **Intense Physical activity** | 1,000 | | | | | | | |
| **Daily time Computer use** | -,029 | 1,000 | | | | | | |
| **Ratio Pauses Hour** | ,020 | -,437 | 1,000 | | | | | |
| **Adequate ICT devices** | ,063 | ,041 | -,002 | 1,000 | | | | |

| | Intense Physical activity | Daily time Computer use | Ratio Pauses Hour | Adequate ICT devices | Adequate furniture | Age | MentalWLFac | MSD Neck |
|---|---|---|---|---|---|---|---|---|
| **Adequate furniture** | ,007 | ,182 | -,101 | ,446 | 1,000 | | | |
| **Age** | -,107 | -,004 | ,010 | -,077 | -,002 | 1,000 | | |
| **MentalWLFac** | ,052 | ,220 | -,136 | -,088 | -,047 | -,071 | 1,000 | |
| **MSD Neck** | ,088 | ,145 | -,053 | -,119 | -,068 | -,059 | ,443 | 1,000 |

**Implied (for all variables) Means (Men - Default model)**

| | Intense Physical activity | Daily time Computer use | Ratio Pauses Hour | Adequate ICT devices | Adequate furniture | Age | MentalWLFac | MSD Neck |
|---|---|---|---|---|---|---|---|---|
| | 4,304 | 4,780 | ,859 | ,626 | ,656 | 45,004 | -,003 | 4,480 |

**Implied Covariances (Men - Default model)**

| | Intense Physical activity | Daily time Computer use | Ratio Pauses Hour | Adequate ICT devices | Adequate furniture | Age | MentalWLFac | MSD Neck |
|---|---|---|---|---|---|---|---|---|
| **Intense Physical activity** | 9,312 | | | | | | | |
| **Daily time Computer use** | -,233 | 7,139 | | | | | | |
| **Ratio Pauses Hour** | ,057 | -1,095 | ,879 | | | | | |
| **Adequate ICT devices** | ,093 | ,053 | -,001 | ,234 | | | | |
| **Adequate furniture** | ,011 | ,230 | -,045 | ,102 | ,225 | | | |
| **Age** | -2,857 | -,094 | ,085 | -,326 | -,009 | 77,161 | | |
| **MentalWLFac** | ,152 | ,561 | -,121 | -,041 | -,021 | -,594 | ,908 | |
| **MSD Neck** | ,740 | 1,066 | -,137 | -,159 | -,089 | -1,433 | 1,162 | 7,578 |

**Implied Correlations (Men - Default model)**

| | Intense Physical activity | Daily time Computer use | Ratio Pauses Hour | Adequate ICT devices | Adequate furniture | Age | MentalWLFac | MSD Neck |
|---|---|---|---|---|---|---|---|---|
| **Intense Physical activity** | 1,000 | | | | | | | |
| **Daily time Computer use** | -,029 | 1,000 | | | | | | |
| **Ratio Pauses Hour** | ,020 | -,437 | 1,000 | | | | | |
| **Adequate ICT devices** | ,063 | ,041 | -,002 | 1,000 | | | | |
| **Adequate furniture** | ,007 | ,182 | -,101 | ,446 | 1,000 | | | |
| **Age** | -,107 | -,004 | ,010 | -,077 | -,002 | 1,000 | | |
| **MentalWLFac** | ,052 | ,220 | -,136 | -,088 | -,047 | -,071 | 1,000 | |
| **MSD Neck** | ,088 | ,145 | -,053 | -,119 | -,068 | -,059 | ,443 | 1,000 |

**Implied Means (Men - Default model)**

| | Intense Physical activity | Daily time Computer use | Ratio Pauses Hour | Adequate ICT devices | Adequate furniture | Age | MentalWLFac | MSD Neck |
|---|---|---|---|---|---|---|---|---|
| | 4,304 | 4,780 | ,859 | ,626 | ,656 | 45,004 | -,003 | 4,480 |

**Factor Score Weights (Men - Default model)**

▫

**Total Effects (Men - Default model)**

| | Intense Physical activity | Daily time Computer use | Ratio Pauses Hour | Adequate ICT devices | Adequate furniture | Age | MentalWLFac |
|---|---|---|---|---|---|---|---|
| **MentalWLFac** | ,018 | ,076 | -,049 | -,162 | -,109 | -,008 | ,000 |
| **MSD Neck** | ,085 | ,171 | ,040 | -,659 | -,270 | -,018 | 1,203 |

**Standardized Total Effects (Men - Default model)**

| | Intense Physical activity | Daily time Computer use | Ratio Pauses Hour | Adequate ICT devices | Adequate furniture | Age | MentalWLFac |
|---|---|---|---|---|---|---|---|
| **MentalWLFac** | ,058 | ,214 | -,048 | -,082 | -,054 | -,070 | ,000 |
| **MSD Neck** | ,094 | ,166 | ,014 | -,116 | -,046 | -,058 | ,417 |

**Direct Effects (Men - Default model)**

| | Intense Physical activity | Daily time Computer use | Ratio Pauses Hour | Adequate ICT devices | Adequate furniture | Age | MentalWLFac |
|---|---|---|---|---|---|---|---|
| **MentalWLFac** | ,018 | ,076 | -,049 | -,162 | -,109 | -,008 | ,000 |
| **MSD Neck** | ,063 | ,080 | ,099 | -,464 | -,138 | -,009 | 1,203 |

**Standardized Direct Effects (Men - Default model)**

| | Intense Physical activity | Daily time Computer use | Ratio Pauses Hour | Adequate ICT devices | Adequate furniture | Age | MentalWLFac |
|---|---|---|---|---|---|---|---|
| **MentalWLFac** | ,058 | ,214 | -,048 | -,082 | -,054 | -,070 | ,000 |
| **MSD Neck** | ,070 | ,077 | ,034 | -,082 | -,024 | -,029 | ,417 |

**Indirect Effects (Men - Default model)**

| | Intense Physical activity | Daily time Computer use | Ratio Pauses Hour | Adequate ICT devices | Adequate furniture | Age | MentalWLFac |
|---|---|---|---|---|---|---|---|
| **MentalWLFac** | ,000 | ,000 | ,000 | ,000 | ,000 | ,000 | ,000 |
| **MSD Neck** | ,022 | ,092 | -,059 | -,194 | -,131 | -,009 | ,000 |

**Standardized Indirect Effects (Men - Default model)**

| | Intense Physical activity | Daily time Computer use | Ratio Pauses Hour | Adequate ICT devices | Adequate furniture | Age | MentalWLFac |
|---|---|---|---|---|---|---|---|
| **MentalWLFac** | ,000 | ,000 | ,000 | ,000 | ,000 | ,000 | ,000 |
| **MSD Neck** | ,024 | ,089 | -,020 | -,034 | -,023 | -,029 | ,000 |

**Women (Women - Default model)**
**Estimates (Women - Default model)**
**Scalar Estimates (Women - Default model)**
**Maximum Likelihood Estimates**

**Regression Weights: (Women - Default model)**

| | | | Estimate | S.E. | C.R. | P | Label |
|---|---|---|---|---|---|---|---|
| MentalWLFac | <--- | Age | ,002 | ,005 | ,467 | ,641 | par_38 |
| MentalWLFac | <--- | Adequate furniture | -,170 | ,095 | -1,783 | ,075 | par_40 |
| MentalWLFac | <--- | Adequate ICT devices | -,256 | ,091 | -2,813 | ,005 | par_41 |
| MentalWLFac | <--- | Daily time Computer use | ,075 | ,021 | 3,677 | *** | par_46 |
| MentalWLFac | <--- | Ratio Pauses Hour | -,302 | ,077 | -3,927 | *** | par_47 |
| MentalWLFac | <--- | Intense Physical activity | ,013 | ,016 | ,826 | ,409 | par_63 |
| MSD Neck | <--- | MentalWLFac | 1,150 | ,105 | 11,005 | *** | par_37 |
| MSD Neck | <--- | Age | -,022 | ,012 | -1,855 | ,064 | par_39 |
| MSD Neck | <--- | Ratio Pauses Hour | ,238 | ,196 | 1,215 | ,224 | par_42 |
| MSD Neck | <--- | Daily time Computer use | ,109 | ,052 | 2,099 | ,036 | par_43 |
| MSD Neck | <--- | Adequate furniture | -,476 | ,240 | -1,986 | ,047 | par_44 |
| MSD Neck | <--- | Adequate ICT devices | -,590 | ,229 | -2,576 | ,010 | par_45 |
| MSD Neck | <--- | Intense Physical activity | -,013 | ,039 | -,325 | ,745 | par_64 |

**Standardized Regression Weights: (Women - Default model)**

| | | | Estimate |
|---|---|---|---|
| MentalWLFac | <--- | Age | ,018 |
| MentalWLFac | <--- | Adequate furniture | -,076 |
| MentalWLFac | <--- | Adequate ICT devices | -,121 |
| MentalWLFac | <--- | Daily time Computer use | ,158 |
| MentalWLFac | <--- | Ratio Pauses Hour | -,169 |
| MentalWLFac | <--- | Intense Physical activity | ,031 |
| MSD Neck | <--- | MentalWLFac | ,413 |
| MSD Neck | <--- | Age | -,064 |
| MSD Neck | <--- | Ratio Pauses Hour | ,048 |
| MSD Neck | <--- | Daily time Computer use | ,082 |
| MSD Neck | <--- | Adequate furniture | -,076 |
| MSD Neck | <--- | Adequate ICT devices | -,100 |
| MSD Neck | <--- | Intense Physical activity | -,011 |

**Means: (Women - Default model)**

| | Estimate | S.E. | C.R. | P | Label |
|---|---|---|---|---|---|
| **Daily time Computer use** | 5,791 | ,084 | 68,910 | *** | par_66 |
| **Ratio Pauses Hour** | ,514 | ,023 | 22,583 | *** | par_68 |
| **Age** | 42,925 | ,324 | 132,541 | *** | par_69 |
| **Adequate ICT devices** | ,600 | ,019 | 31,673 | *** | par_70 |
| **Adequate furniture** | ,686 | ,018 | 38,272 | *** | par_71 |

| | Estimate | S.E. | C.R. | P | Label |
|---|---|---|---|---|---|
| **Intense Physical activity** | 2,674 | ,098 | 27,229 | *** | par_72 |

**Intercepts: (Women - Default model)**

| | Estimate | S.E. | C.R. | P | Label |
|---|---|---|---|---|---|
| **MentalWLFac** | -,143 | ,268 | -,534 | ,593 | par_65 |
| **MSD Neck** | 6,722 | ,670 | 10,032 | *** | par_67 |

**Covariances: (Women - Default model)**

| | | | Estimate | S.E. | C.R. | P | Label |
|---|---|---|---|---|---|---|---|
| Adequate furniture | <--> | Adequate ICT devices | ,100 | ,010 | 10,417 | *** | par_48 |
| Adequate ICT devices | <--> | Daily time Computer use | ,006 | ,041 | ,138 | ,890 | par_49 |
| Adequate furniture | <--> | Daily time Computer use | ,077 | ,039 | 1,965 | ,049 | par_50 |
| Adequate furniture | <--> | Ratio Pauses Hour | -,001 | ,011 | -,071 | ,943 | par_51 |
| Ratio Pauses Hour | <--> | Daily time Computer use | -,553 | ,057 | -9,736 | *** | par_52 |
| Age | <--> | Ratio Pauses Hour | ,201 | ,191 | 1,053 | ,292 | par_53 |
| Ratio Pauses Hour | <--> | Intense Physical activity | ,033 | ,058 | ,577 | ,564 | par_54 |
| Age | <--> | Daily time Computer use | -1,372 | ,706 | -1,942 | ,052 | par_55 |
| Age | <--> | Adequate furniture | -,057 | ,151 | -,381 | ,703 | par_56 |
| Age | <--> | Adequate ICT devices | -,289 | ,160 | -1,812 | ,070 | par_57 |
| Age | <--> | Intense Physical activity | -,665 | ,823 | -,808 | ,419 | par_58 |
| Daily time Computer use | <--> | Intense Physical activity | -,160 | ,214 | -,751 | ,453 | par_59 |
| Adequate furniture | <--> | Intense Physical activity | ,036 | ,046 | ,783 | ,434 | par_60 |
| Adequate ICT devices | <--> | Intense Physical activity | ,091 | ,048 | 1,882 | ,060 | par_61 |
| Adequate ICT devices | <--> | Ratio Pauses Hour | ,023 | ,011 | 2,035 | ,042 | par_62 |

**Correlations: (Women - Default model)**

| | | | Estimate |
|---|---|---|---|
| Adequate furniture | <--> | Adequate ICT devices | ,440 |
| Adequate ICT devices | <--> | Daily time Computer use | ,005 |
| Adequate furniture | <--> | Daily time Computer use | ,076 |
| Adequate furniture | <--> | Ratio Pauses Hour | -,003 |
| Ratio Pauses Hour | <--> | Daily time Computer use | -,436 |
| Age | <--> | Ratio Pauses Hour | ,041 |
| Ratio Pauses Hour | <--> | Intense Physical activity | ,023 |
| Age | <--> | Daily time Computer use | -,075 |
| Age | <--> | Adequate furniture | -,015 |
| Age | <--> | Adequate ICT devices | -,070 |
| Age | <--> | Intense Physical activity | -,031 |

| | | | Estimate |
|---|---|---|---|
| Daily time Computer use | <--> | Intense Physical activity | -,029 |
| Adequate furniture | <--> | Intense Physical activity | ,030 |
| Adequate ICT devices | <--> | Intense Physical activity | ,073 |
| Adequate ICT devices | <--> | Ratio Pauses Hour | ,080 |

**Variances: (Women - Default model)**

| | Estimate | S.E. | C.R. | P | Label |
|---|---|---|---|---|---|
| **Age** | 70,263 | 3,839 | 18,301 | *** | par_81 |
| **Adequate furniture** | ,215 | ,012 | 18,288 | *** | par_82 |
| **Adequate ICT devices** | ,240 | ,013 | 18,289 | *** | par_83 |
| **Ratio Pauses Hour** | ,340 | ,019 | 17,621 | *** | par_84 |
| **Daily time Computer use** | 4,731 | ,259 | 18,301 | *** | par_85 |
| **Intense Physical activity** | 6,458 | ,353 | 18,301 | *** | par_86 |
| **eMWL** | ,966 | ,056 | 17,340 | *** | par_87 |
| **eMSD** | 6,483 | ,361 | 17,948 | *** | par_88 |

**Squared Multiple Correlations: (Women - Default model)**

| | Estimate |
|---|---|
| **MentalWLFac** | ,106 |
| **MSD Neck** | ,227 |

**Matrices (Women - Default model)**
**Implied (for all variables) Covariances (Women - Default model)**

| | Intense Physical activity | Daily time Computer use | Ratio Pauses Hour | Adequate ICT devices | Adequate furniture | Age | MentalWLFac | MSD Neck |
|---|---|---|---|---|---|---|---|---|
| **Intense Physical activity** | 6,458 | | | | | | | |
| **Daily time Computer use** | -,160 | 4,731 | | | | | | |
| **Ratio Pauses Hour** | ,033 | -,553 | ,340 | | | | | |
| **Adequate ICT devices** | ,091 | ,006 | ,023 | ,240 | | | | |
| **Adequate furniture** | ,036 | ,077 | -,001 | ,100 | ,215 | | | |
| **Age** | -,665 | -1,372 | ,201 | -,289 | -,057 | 70,263 | | |
| **MentalWLFac** | ,030 | ,504 | -,149 | -,084 | -,056 | ,067 | 1,081 | |
| **MSD Neck** | -,113 | ,955 | -,169 | -,275 | -,217 | -1,371 | 1,337 | 8,382 |

**Implied (for all variables) Correlations (Women - Default model)**

| | Intense Physical activity | Daily time Computer use | Ratio Pauses Hour | Adequate ICT devices | Adequate furniture | Age | MentalWLFac | MSD Neck |
|---|---|---|---|---|---|---|---|---|
| **Intense Physical activity** | 1,000 | | | | | | | |
| **Daily time Computer use** | -,029 | 1,000 | | | | | | |

| | Intense Physical activity | Daily time Computer use | Ratio Pauses Hour | Adequate ICT devices | Adequate furniture | Age | MentalWLFac | MSD Neck |
|---|---|---|---|---|---|---|---|---|
| **Ratio Pauses Hour** | ,023 | -,436 | 1,000 | | | | | |
| **Adequate ICT devices** | ,073 | ,005 | ,080 | 1,000 | | | | |
| **Adequate furniture** | ,030 | ,076 | -,003 | ,440 | 1,000 | | | |
| **Age** | -,031 | -,075 | ,041 | -,070 | -,015 | 1,000 | | |
| **MentalWLFac** | ,011 | ,223 | -,246 | -,166 | -,116 | ,008 | 1,000 | |
| **MSD Neck** | -,015 | ,152 | -,100 | -,194 | -,161 | -,056 | ,444 | 1,000 |

**Implied (for all variables) Means (Women - Default model)**

| | Intense Physical activity | Daily time Computer use | Ratio Pauses Hour | Adequate ICT devices | Adequate furniture | Age | MentalWLFac | MSD Neck |
|---|---|---|---|---|---|---|---|---|
| | 2,674 | 5,791 | ,514 | ,600 | ,686 | 42,925 | -,002 | 5,808 |

**Implied Covariances (Women - Default model)**

| | Intense Physical activity | Daily time Computer use | Ratio Pauses Hour | Adequate ICT devices | Adequate furniture | Age | MentalWLFac | MSD Neck |
|---|---|---|---|---|---|---|---|---|
| **Intense Physical activity** | 6,458 | | | | | | | |
| **Daily time Computer use** | -,160 | 4,731 | | | | | | |
| **Ratio Pauses Hour** | ,033 | -,553 | ,340 | | | | | |
| **Adequate ICT devices** | ,091 | ,006 | ,023 | ,240 | | | | |
| **Adequate furniture** | ,036 | ,077 | -,001 | ,100 | ,215 | | | |
| **Age** | -,665 | -1,372 | ,201 | -,289 | -,057 | 70,263 | | |
| **MentalWLFac** | ,030 | ,504 | -,149 | -,084 | -,056 | ,067 | 1,081 | |
| **MSD Neck** | -,113 | ,955 | -,169 | -,275 | -,217 | -1,371 | 1,337 | 8,382 |

**Implied Correlations (Women - Default model)**

| | Intense Physical activity | Daily time Computer use | Ratio Pauses Hour | Adequate ICT devices | Adequate furniture | Age | MentalWLFac | MSD Neck |
|---|---|---|---|---|---|---|---|---|
| **Intense Physical activity** | 1,000 | | | | | | | |
| **Daily time Computer use** | -,029 | 1,000 | | | | | | |
| **Ratio Pauses Hour** | ,023 | -,436 | 1,000 | | | | | |
| **Adequate ICT devices** | ,073 | ,005 | ,080 | 1,000 | | | | |
| **Adequate furniture** | ,030 | ,076 | -,003 | ,440 | 1,000 | | | |
| **Age** | -,031 | -,075 | ,041 | -,070 | -,015 | 1,000 | | |
| **MentalWLFac** | ,011 | ,223 | -,246 | -,166 | -,116 | ,008 | 1,000 | |
| **MSD Neck** | -,015 | ,152 | -,100 | -,194 | -,161 | -,056 | ,444 | 1,000 |

**Implied Means (Women - Default model)**

| | Intense Physical activity | Daily time Computer use | Ratio Pauses Hour | Adequate ICT devices | Adequate furniture | Age | MentalWLFac | MSD Neck |
|---|---|---|---|---|---|---|---|---|
| | 2,674 | 5,791 | ,514 | ,600 | ,686 | 42,925 | -,002 | 5,808 |

**Factor Score Weights (Women - Default model)**

¤

**Total Effects (Women - Default model)**

| | Intense Physical activity | Daily time Computer use | Ratio Pauses Hour | Adequate ICT devices | Adequate furniture | Age | MentalWLFac |
|---|---|---|---|---|---|---|---|
| **MentalWLFac** | ,013 | ,075 | -,302 | -,256 | -,170 | ,002 | ,000 |
| **MSD Neck** | ,002 | ,196 | -,109 | -,885 | -,672 | -,020 | 1,150 |

**Standardized Total Effects (Women - Default model)**

| | Intense Physical activity | Daily time Computer use | Ratio Pauses Hour | Adequate ICT devices | Adequate furniture | Age | MentalWLFac |
|---|---|---|---|---|---|---|---|
| **MentalWLFac** | ,031 | ,158 | -,169 | -,121 | -,076 | ,018 | ,000 |
| **MSD Neck** | ,002 | ,147 | -,022 | -,150 | -,108 | -,057 | ,413 |

**Direct Effects (Women - Default model)**

| | Intense Physical activity | Daily time Computer use | Ratio Pauses Hour | Adequate ICT devices | Adequate furniture | Age | MentalWLFac |
|---|---|---|---|---|---|---|---|
| **MentalWLFac** | ,013 | ,075 | -,302 | -,256 | -,170 | ,002 | ,000 |
| **MSD Neck** | -,013 | ,109 | ,238 | -,590 | -,476 | -,022 | 1,150 |

**Standardized Direct Effects (Women - Default model)**

| | Intense Physical activity | Daily time Computer use | Ratio Pauses Hour | Adequate ICT devices | Adequate furniture | Age | MentalWLFac |
|---|---|---|---|---|---|---|---|
| **MentalWLFac** | ,031 | ,158 | -,169 | -,121 | -,076 | ,018 | ,000 |
| **MSD Neck** | -,011 | ,082 | ,048 | -,100 | -,076 | -,064 | ,413 |

**Indirect Effects (Women - Default model)**

| | Intense Physical activity | Daily time Computer use | Ratio Pauses Hour | Adequate ICT devices | Adequate furniture | Age | MentalWLFac |
|---|---|---|---|---|---|---|---|
| **MentalWLFac** | ,000 | ,000 | ,000 | ,000 | ,000 | ,000 | ,000 |
| **MSD Neck** | ,015 | ,087 | -,347 | -,295 | -,196 | ,003 | ,000 |

**Standardized Indirect Effects (Women - Default model)**

| | Intense Physical activity | Daily time Computer use | Ratio Pauses Hour | Adequate ICT devices | Adequate furniture | Age | MentalWLFac |
|---|---|---|---|---|---|---|---|
| **MentalWLFac** | ,000 | ,000 | ,000 | ,000 | ,000 | ,000 | ,000 |
| **MSD Neck** | ,013 | ,065 | -,070 | -,050 | -,031 | ,007 | ,000 |

**Minimization History (Default model)**

| Iteration | | Negative eigenvalues | Condition # | Smallest eigenvalue | Diameter | F | NTries | Ratio |
|---|---|---|---|---|---|---|---|---|
| **0** | e | 10 | | -,837 | 9999,000 | 43206,672 | 0 | 9999,000 |
| **1** | e | 9 | | -,234 | ,657 | 13153,802 | 12 | ,811 |
| **2** | e | 7 | | -,297 | 1,275 | 3377,663 | 8 | ,846 |
| **3** | e* | 2 | | -,211 | ,813 | 1485,127 | 5 | 1,007 |
| **4** | e | 0 | 71418,689 | | ,332 | 972,868 | 5 | ,948 |
| **5** | e | 0 | 46233,620 | | ,527 | 841,037 | 6 | ,000 |
| **6** | e | 0 | 41362,095 | | 1,000 | 385,784 | 2 | ,000 |
| **7** | e | 0 | 38361,400 | | 1,161 | 55,967 | 1 | 1,184 |
| **8** | e | 0 | 36545,991 | | ,522 | 4,116 | 1 | 1,145 |
| **9** | e | 0 | 36747,161 | | ,207 | ,080 | 1 | 1,079 |
| **10** | e | 0 | 36514,347 | | ,037 | ,000 | 1 | 1,020 |
| **11** | e | 0 | 36547,814 | | ,001 | ,000 | 1 | 1,001 |
| **12** | e | 0 | 36547,814 | | ,000 | ,000 | 1 | 1,000 |

**Miscellaneous**
**Model Fit Summary**
**CMIN**

| Model | NPAR | CMIN | DF | P | CMIN/DF |
|---|---|---|---|---|---|
| **Default model** | 88 | ,000 | 0 | | |
| **Saturated model** | 88 | ,000 | 0 | | |
| **Independence model** | 16 | 897,377 | 72 | ,000 | 12,464 |

**Baseline Comparisons**

| Model | NFI Delta1 | RFI rho1 | IFI Delta2 | TLI rho2 | CFI |
|---|---|---|---|---|---|
| **Default model** | 1,000 | | 1,000 | | 1,000 |
| **Saturated model** | 1,000 | | 1,000 | | 1,000 |
| **Independence model** | ,000 | ,000 | ,000 | ,000 | ,000 |

**Parsimony-Adjusted Measures**

| Model | PRATIO | PNFI | PCFI |
|---|---|---|---|
| **Default model** | ,000 | ,000 | ,000 |
| **Saturated model** | ,000 | ,000 | ,000 |
| **Independence model** | 1,000 | ,000 | ,000 |

**NCP**

| Model | NCP | LO 90 | HI 90 |
|---|---|---|---|
| Default model | ,000 | ,000 | ,000 |
| Saturated model | ,000 | ,000 | ,000 |
| Independence model | 825,377 | 732,504 | 925,682 |

**FMIN**

| Model | FMIN | F0 | LO 90 | HI 90 |
|---|---|---|---|---|
| Default model | ,000 | ,000 | ,000 | ,000 |
| Saturated model | ,000 | ,000 | ,000 | ,000 |
| Independence model | ,750 | ,690 | ,612 | ,774 |

**RMSEA**

| Model | RMSEA | LO 90 | HI 90 | PCLOSE |
|---|---|---|---|---|
| Independence model | ,098 | ,092 | ,104 | ,000 |

**AIC**

| Model | AIC | BCC | BIC | CAIC |
|---|---|---|---|---|
| Default model | 176,000 | 178,735 | | |
| Saturated model | 176,000 | 178,735 | | |
| Independence model | 929,377 | 929,874 | | |

**ECVI**

| Model | ECVI | LO 90 | HI 90 | MECVI |
|---|---|---|---|---|
| Default model | ,147 | ,147 | ,147 | ,149 |
| Saturated model | ,147 | ,147 | ,147 | ,149 |
| Independence model | ,777 | ,699 | ,861 | ,777 |

**HOELTER**

| Model | HOELTER .05 | HOELTER .01 |
|---|---|---|
| Default model | | |
| Independence model | 125 | 139 |

**Execution time summary**

**Minimization:** ,035

| | |
|---|---|
| **Miscellaneous:** | ,534 |
| **Bootstrap:** | ,000 |
| **Total:** | ,569 |

## 2. MSD SHOULDERS

**Groups**

**Group number 1 (Group number 1= Men )**
**Notes for Group (Group number 1= Men )**

The model is recursive.
Sample size = 527

**Variable Summary (Men)**

**Your model contains the following variables (Men)**

Observed, endogenous variables
MSD Shoulders
MentalWLFac
Observed, exogenous variables
Age
Adequate furniture
Adequate ICT devices
Ratio Pauses Hour
Daily time Computer use
Intense Physical activity
Unobserved, exogenous variables
eMSD
eMWL

**Variable counts (Men)**

| | |
|---|---|
| **Number of variables in your model:** | 10 |
| **Number of observed variables:** | 8 |
| **Number of unobserved variables:** | 2 |
| **Number of exogenous variables:** | 8 |
| **Number of endogenous variables:** | 2 |

**Parameter Summary (Men)**

| | Weights | Covariances | Variances | Means | Intercepts | Total |
|---|---|---|---|---|---|---|
| **Fixed** | 2 | 0 | 0 | 2 | 0 | 4 |
| **Labeled** | 0 | 0 | 0 | 0 | 0 | 0 |
| **Unlabeled** | 13 | 15 | 8 | 6 | 2 | 44 |
| **Total** | 15 | 15 | 8 | 8 | 2 | 48 |

**Group number 2 (Group number 2)**

**Notes for Group (Group number 2)**

The model is recursive.
Sample size = 671

**Variable Summary (Women)**

**Your model contains the following variables (Women)**

Observed, endogenous variables
MSD Shoulders
MentalWLFac
Observed, exogenous variables
Age
Adequate furniture
Adequate ICT devices
Ratio Pauses Hour
Daily time Computer use
Intense Physical activity
Unobserved, exogenous variables
eMSD
eMWL

**Variable counts (Women)**

| | |
|---|---|
| **Number of variables in your model:** | 10 |
| **Number of observed variables:** | 8 |
| **Number of unobserved variables:** | 2 |
| **Number of exogenous variables:** | 8 |
| **Number of endogenous variables:** | 2 |

**Parameter Summary (Women)**

| | Weights | Covariances | Variances | Means | Intercepts | Total |
|---|---|---|---|---|---|---|
| **Fixed** | 2 | 0 | 0 | 2 | 0 | 4 |
| **Labeled** | 0 | 0 | 0 | 0 | 0 | 0 |
| **Unlabeled** | 13 | 15 | 8 | 6 | 2 | 44 |
| **Total** | 15 | 15 | 8 | 8 | 2 | 48 |

**Models**

**Default model (Default model)**

**Notes for Model (Default model)**

**Computation of degrees of freedom (Default model)**

| | |
|---|---|
| **Number of distinct sample moments:** | 88 |
| **Number of distinct parameters to be estimated:** | 88 |
| **Degrees of freedom (88 - 88):** | 0 |

**Result (Default model)**

Minimum was achieved
Chi-square = ,000
Degrees of freedom = 0
Probability level cannot be computed

**Men (Men - Default model)**

**Estimates (Men - Default model)**

**Scalar Estimates (Men - Default model)**

**Maximum Likelihood Estimates**

**Regression Weights: (Men - Default model)**

| | | | Estimate | S.E. | C.R. | P | Label |
|---|---|---|---|---|---|---|---|
| MentalWLFac | <--- | Age | -,007 | ,005 | -1,522 | ,128 | par_2 |
| MentalWLFac | <--- | Adequate furniture | -,106 | ,097 | -1,090 | ,276 | par_4 |
| MentalWLFac | <--- | Adequate ICT devices | -,162 | ,094 | -1,711 | ,087 | par_5 |
| MentalWLFac | <--- | Daily time Computer use | ,079 | ,018 | 4,459 | *** | par_10 |
| MentalWLFac | <--- | Ratio Pauses Hour | -,046 | ,054 | -,841 | ,400 | par_11 |
| MentalWLFac | <--- | Intense Physical activity | ,019 | ,014 | 1,382 | ,167 | par_27 |
| MSD Shoulders | <--- | MentalWLFac | ,970 | ,108 | 8,957 | *** | par_1 |
| MSD Shoulders | <--- | Age | ,009 | ,011 | ,810 | ,418 | par_3 |
| MSD Shoulders | <--- | Ratio Pauses Hour | ,324 | ,120 | 2,709 | ,007 | par_6 |
| MSD Shoulders | <--- | Daily time Computer use | -,007 | ,042 | -,173 | ,862 | par_7 |
| MSD Shoulders | <--- | Adequate furniture | -,007 | ,235 | -,030 | ,976 | par_8 |
| MSD Shoulders | <--- | Adequate ICT devices | -,469 | ,229 | -2,052 | ,040 | par_9 |
| MSD Shoulders | <--- | Intense Physical activity | ,037 | ,032 | 1,146 | ,252 | par_28 |

**Standardized Regression Weights: (Men - Default model)**

| | | | Estimate |
|---|---|---|---|
| MentalWLFac | <--- | Age | -,066 |
| MentalWLFac | <--- | Adequate furniture | -,053 |
| MentalWLFac | <--- | Adequate ICT devices | -,082 |
| MentalWLFac | <--- | Daily time Computer use | ,221 |
| MentalWLFac | <--- | Ratio Pauses Hour | -,045 |
| MentalWLFac | <--- | Intense Physical activity | ,060 |
| MSD Shoulders | <--- | MentalWLFac | ,378 |
| MSD Shoulders | <--- | Age | ,033 |
| MSD Shoulders | <--- | Ratio Pauses Hour | ,124 |
| MSD Shoulders | <--- | Daily time Computer use | -,008 |
| MSD Shoulders | <--- | Adequate furniture | -,001 |
| MSD Shoulders | <--- | Adequate ICT devices | -,093 |
| MSD Shoulders | <--- | Intense Physical activity | ,046 |

**Means: (Men - Default model)**

| | Estimate | S.E. | C.R. | P | Label |
|---|---|---|---|---|---|
| **Daily time Computer use** | 4,780 | ,116 | 41,033 | *** | par_30 |
| **Ratio Pauses Hour** | ,860 | ,043 | 19,917 | *** | par_32 |
| **Age** | 45,004 | ,383 | 117,515 | *** | par_33 |
| **Adequate ICT devices** | ,626 | ,021 | 29,584 | *** | par_34 |
| **Adequate furniture** | ,656 | ,021 | 31,615 | *** | par_35 |
| **Intense Physical activity** | 4,304 | ,133 | 32,348 | *** | par_36 |

**Intercepts: (Men - Default model)**

| | Estimate | S.E. | C.R. | P | Label |
|---|---|---|---|---|---|
| **MentalWLFac** | ,072 | ,265 | ,273 | ,785 | par_29 |
| **MSD Shoulders** | 2,685 | ,625 | 4,299 | *** | par_31 |

**Covariances: (Men - Default model)**

| | | | Estimate | S.E. | C.R. | P | Label |
|---|---|---|---|---|---|---|---|
| Adequate furniture | <--> | Adequate ICT devices | ,102 | ,011 | 9,313 | *** | par_12 |
| Adequate ICT devices | <--> | Daily time Computer use | ,053 | ,057 | ,931 | ,352 | par_13 |
| Adequate furniture | <--> | Daily time Computer use | ,230 | ,056 | 4,089 | *** | par_14 |
| Adequate furniture | <--> | Ratio Pauses Hour | -,045 | ,021 | -2,148 | ,032 | par_15 |
| Ratio Pauses Hour | <--> | Daily time Computer use | -1,098 | ,136 | -8,045 | *** | par_16 |
| Age | <--> | Ratio Pauses Hour | ,086 | ,371 | ,233 | ,816 | par_17 |
| Ratio Pauses Hour | <--> | Intense Physical activity | ,060 | ,130 | ,466 | ,641 | par_18 |
| Age | <--> | Daily time Computer use | -,094 | 1,023 | -,092 | ,927 | par_19 |

| | | | Estimate | S.E. | C.R. | P | Label |
|---|---|---|---|---|---|---|---|
| Age | <--> | Adequate furniture | -,009 | ,182 | -,049 | ,961 | par_20 |
| Age | <--> | Adequate ICT devices | -,327 | ,186 | -1,757 | ,079 | par_21 |
| Age | <--> | Intense Physical activity | -2,857 | 1,175 | -2,431 | ,015 | par_22 |
| Daily time Computer use | <--> | Intense Physical activity | -,233 | ,356 | -,655 | ,512 | par_23 |
| Adequate furniture | <--> | Intense Physical activity | ,010 | ,063 | ,163 | ,870 | par_24 |
| Adequate ICT devices | <--> | Intense Physical activity | ,092 | ,065 | 1,428 | ,153 | par_25 |
| Adequate ICT devices | <--> | Ratio Pauses Hour | -,001 | ,021 | -,055 | ,956 | par_26 |

**Correlations: (Men - Default model)**

| | | | Estimate |
|---|---|---|---|
| Adequate furniture | <--> | Adequate ICT devices | ,446 |
| Adequate ICT devices | <--> | Daily time Computer use | ,041 |
| Adequate furniture | <--> | Daily time Computer use | ,182 |
| Adequate furniture | <--> | Ratio Pauses Hour | -,100 |
| Ratio Pauses Hour | <--> | Daily time Computer use | -,438 |
| Age | <--> | Ratio Pauses Hour | ,010 |
| Ratio Pauses Hour | <--> | Intense Physical activity | ,021 |
| Age | <--> | Daily time Computer use | -,004 |
| Age | <--> | Adequate furniture | -,002 |
| Age | <--> | Adequate ICT devices | -,077 |
| Age | <--> | Intense Physical activity | -,107 |
| Daily time Computer use | <--> | Intense Physical activity | -,029 |
| Adequate furniture | <--> | Intense Physical activity | ,007 |
| Adequate ICT devices | <--> | Intense Physical activity | ,063 |
| Adequate ICT devices | <--> | Ratio Pauses Hour | -,002 |

**Variances: (Men - Default model)**

| | Estimate | S.E. | C.R. | P | Label |
|---|---|---|---|---|---|
| **Age** | 77,161 | 4,757 | 16,219 | *** | par_73 |
| **Adequate furniture** | ,225 | ,014 | 16,176 | *** | par_74 |
| **Adequate ICT devices** | ,234 | ,014 | 16,174 | *** | par_75 |
| **Ratio Pauses Hour** | ,880 | ,060 | 14,709 | *** | par_76 |
| **Daily time Computer use** | 7,139 | ,440 | 16,219 | *** | par_77 |
| **Intense Physical activity** | 9,312 | ,574 | 16,219 | *** | par_78 |
| **eMWL** | ,842 | ,053 | 15,892 | *** | par_79 |
| **eMSD** | 4,999 | ,310 | 16,109 | *** | par_80 |

**Squared Multiple Correlations: (Men - Default model)**

| | Estimate |
|---|---|
| **MentalWLFac** | ,073 |
| **MSD Shoulders** | ,164 |

**Matrices (Men - Default model)**

**Implied (for all variables) Covariances (Men - Default model)**

| | Intense Physical activity | Daily time Computer use | Ratio Pauses Hour | Adequate ICT devices | Adequate furniture | Age | MentalWLFac | MSD Shoulders |
|---|---|---|---|---|---|---|---|---|
| **Intense Physical activity** | 9,312 | | | | | | | |
| **Daily time Computer use** | -,233 | 7,139 | | | | | | |
| **Ratio Pauses Hour** | ,060 | -1,098 | ,880 | | | | | |
| **Adequate ICT devices** | ,092 | ,053 | -,001 | ,234 | | | | |
| **Adequate furniture** | ,010 | ,230 | -,045 | ,102 | ,225 | | | |
| **Age** | -2,857 | -,094 | ,086 | -,327 | -,009 | 77,161 | | |
| **MentalWLFac** | ,157 | ,576 | -,121 | -,040 | -,020 | -,560 | ,908 | |
| **MSD Shoulders** | ,450 | ,114 | ,180 | -,150 | -,085 | ,236 | ,857 | 5,977 |

**Implied (for all variables) Correlations (Men - Default model)**

| | Intense Physical activity | Daily time Computer use | Ratio Pauses Hour | Adequate ICT devices | Adequate furniture | Age | MentalWLFac | MSD Shoulders |
|---|---|---|---|---|---|---|---|---|
| **Intense Physical activity** | 1,000 | | | | | | | |
| **Daily time Computer use** | -,029 | 1,000 | | | | | | |
| **Ratio Pauses Hour** | ,021 | -,438 | 1,000 | | | | | |
| **Adequate ICT devices** | ,063 | ,041 | -,002 | 1,000 | | | | |
| **Adequate furniture** | ,007 | ,182 | -,100 | ,446 | 1,000 | | | |
| **Age** | -,107 | -,004 | ,010 | -,077 | -,002 | 1,000 | | |
| **MentalWLFac** | ,054 | ,226 | -,136 | -,088 | -,044 | -,067 | 1,000 | |
| **MSD Shoulders** | ,060 | ,017 | ,078 | -,127 | -,073 | ,011 | ,368 | 1,000 |

**Implied (for all variables) Means (Men - Default model)**

| | Intense Physical activity | Daily time Computer use | Ratio Pauses Hour | Adequate ICT devices | Adequate furniture | Age | MentalWLFac | MSD Shoulders |
|---|---|---|---|---|---|---|---|---|
| | 4,304 | 4,780 | ,860 | ,626 | ,656 | 45,004 | -,001 | 3,199 |

**Implied Covariances (Men - Default model)**

| | Intense Physical activity | Daily time Computer use | Ratio Pauses Hour | Adequate ICT devices | Adequate furniture | Age | MentalWLFac | MSD Shoulders |
|---|---|---|---|---|---|---|---|---|
| **Intense Physical activity** | 9,312 | | | | | | | |
| **Daily time Computer use** | -,233 | 7,139 | | | | | | |
| **Ratio Pauses Hour** | ,060 | -1,098 | ,880 | | | | | |

| | Intense Physical activity | Daily time Computer use | Ratio Pauses Hour | Adequate ICT devices | Adequate furniture | Age | MentalWLFac | MSD Shoulders |
|---|---|---|---|---|---|---|---|---|
| **Adequate ICT devices** | ,092 | ,053 | -,001 | ,234 | | | | |
| **Adequate furniture** | ,010 | ,230 | -,045 | ,102 | ,225 | | | |
| **Age** | -2,857 | -,094 | ,086 | -,327 | -,009 | 77,161 | | |
| **MentalWLFac** | ,157 | ,576 | -,121 | -,040 | -,020 | -,560 | ,908 | |
| **MSD Shoulders** | ,450 | ,114 | ,180 | -,150 | -,085 | ,236 | ,857 | 5,977 |

**Implied Correlations (Men - Default model)**

| | Intense Physical activity | Daily time Computer use | Ratio Pauses Hour | Adequate ICT devices | Adequate furniture | Age | MentalWLFac | MSD Shoulders |
|---|---|---|---|---|---|---|---|---|
| **Intense Physical activity** | 1,000 | | | | | | | |
| **Daily time Computer use** | -,029 | 1,000 | | | | | | |
| **Ratio Pauses Hour** | ,021 | -,438 | 1,000 | | | | | |
| **Adequate ICT devices** | ,063 | ,041 | -,002 | 1,000 | | | | |
| **Adequate furniture** | ,007 | ,182 | -,100 | ,446 | 1,000 | | | |
| **Age** | -,107 | -,004 | ,010 | -,077 | -,002 | 1,000 | | |
| **MentalWLFac** | ,054 | ,226 | -,136 | -,088 | -,044 | -,067 | 1,000 | |
| **MSD Shoulders** | ,060 | ,017 | ,078 | -,127 | -,073 | ,011 | ,368 | 1,000 |

**Implied Means (Men - Default model)**

| | Intense Physical activity | Daily time Computer use | Ratio Pauses Hour | Adequate ICT devices | Adequate furniture | Age | MentalWLFac | MSD Shoulders |
|---|---|---|---|---|---|---|---|---|
| | 4,304 | 4,780 | ,860 | ,626 | ,656 | 45,004 | -,001 | 3,199 |

**Factor Score Weights (Men - Default model)**

**Total Effects (Men - Default model)**

| | Intense Physical activity | Daily time Computer use | Ratio Pauses Hour | Adequate ICT devices | Adequate furniture | Age | MentalWLFac |
|---|---|---|---|---|---|---|---|
| **MentalWLFac** | ,019 | ,079 | -,046 | -,162 | -,106 | -,007 | ,000 |
| **MSD Shoulders** | ,055 | ,069 | ,280 | -,626 | -,110 | ,002 | ,970 |

**Standardized Total Effects (Men - Default model)**

| | Intense Physical activity | Daily time Computer use | Ratio Pauses Hour | Adequate ICT devices | Adequate furniture | Age | MentalWLFac |
|---|---|---|---|---|---|---|---|
| **MentalWLFac** | ,060 | ,221 | -,045 | -,082 | -,053 | -,066 | ,000 |
| **MSD Shoulders** | ,069 | ,075 | ,107 | -,124 | -,021 | ,008 | ,378 |

**Direct Effects (Men - Default model)**

| | Intense Physical activity | Daily time Computer use | Ratio Pauses Hour | Adequate ICT devices | Adequate furniture | Age | MentalWLFac |
|---|---|---|---|---|---|---|---|
| **MentalWLFac** | ,019 | ,079 | -,046 | -,162 | -,106 | -,007 | ,000 |
| **MSD Shoulders** | ,037 | -,007 | ,324 | -,469 | -,007 | ,009 | ,970 |

**Standardized Direct Effects (Men - Default model)**

| | Intense Physical activity | Daily time Computer use | Ratio Pauses Hour | Adequate ICT devices | Adequate furniture | Age | MentalWLFac |
|---|---|---|---|---|---|---|---|
| **MentalWLFac** | ,060 | ,221 | -,045 | -,082 | -,053 | -,066 | ,000 |
| **MSD Shoulders** | ,046 | -,008 | ,124 | -,093 | -,001 | ,033 | ,378 |

**Indirect Effects (Men - Default model)**

| | Intense Physical activity | Daily time Computer use | Ratio Pauses Hour | Adequate ICT devices | Adequate furniture | Age | MentalWLFac |
|---|---|---|---|---|---|---|---|
| **MentalWLFac** | ,000 | ,000 | ,000 | ,000 | ,000 | ,000 | ,000 |
| **MSD Shoulders** | ,018 | ,076 | -,044 | -,157 | -,103 | -,007 | ,000 |

**Standardized Indirect Effects (Men - Default model)**

| | Intense Physical activity | Daily time Computer use | Ratio Pauses Hour | Adequate ICT devices | Adequate furniture | Age | MentalWLFac |
|---|---|---|---|---|---|---|---|
| **MentalWLFac** | ,000 | ,000 | ,000 | ,000 | ,000 | ,000 | ,000 |
| **MSD Shoulders** | ,023 | ,083 | -,017 | -,031 | -,020 | -,025 | ,000 |

**Women (Women - Default model)**

**Estimates (Women - Default model)**

**Scalar Estimates (Women - Default model)**

**Maximum Likelihood Estimates**

**Regression Weights: (Women - Default model)**

| | | | Estimate | S.E. | C.R. | P | Label |
|---|---|---|---|---|---|---|---|
| MentalWLFac | <--- | Age | ,002 | ,005 | ,481 | ,631 | par_38 |
| MentalWLFac | <--- | Adequate furniture | -,167 | ,096 | -1,747 | ,081 | par_40 |
| MentalWLFac | <--- | Adequate ICT devices | -,257 | ,091 | -2,822 | ,005 | par_41 |
| MentalWLFac | <--- | Daily time Computer use | ,077 | ,021 | 3,762 | *** | par_46 |
| MentalWLFac | <--- | Ratio Pauses Hour | -,302 | ,077 | -3,931 | *** | par_47 |
| MentalWLFac | <--- | Intense Physical activity | ,013 | ,016 | ,851 | ,395 | par_63 |
| MSD Shoulders | <--- | MentalWLFac | 1,096 | ,113 | 9,732 | *** | par_37 |
| MSD Shoulders | <--- | Age | -,015 | ,013 | -1,214 | ,225 | par_39 |

| | | | Estimate | S.E. | C.R. | P | Label |
|---|---|---|---|---|---|---|---|
| MSD Shoulders | <--- | Ratio Pauses Hour | -,066 | ,209 | -,314 | ,753 | par_42 |
| MSD Shoulders | <--- | Daily time Computer use | ,066 | ,055 | 1,186 | ,236 | par_43 |
| MSD Shoulders | <--- | Adequate furniture | ,191 | ,257 | ,743 | ,458 | par_44 |
| MSD Shoulders | <--- | Adequate ICT devices | -,650 | ,246 | -2,648 | ,008 | par_45 |
| MSD Shoulders | <--- | Intense Physical activity | ,003 | ,042 | ,078 | ,938 | par_64 |

**Standardized Regression Weights: (Women - Default model)**

| | | | Estimate |
|---|---|---|---|
| MentalWLFac | <--- | Age | ,018 |
| MentalWLFac | <--- | Adequate furniture | -,074 |
| MentalWLFac | <--- | Adequate ICT devices | -,121 |
| MentalWLFac | <--- | Daily time Computer use | ,162 |
| MentalWLFac | <--- | Ratio Pauses Hour | -,169 |
| MentalWLFac | <--- | Intense Physical activity | ,032 |
| MSD Shoulders | <--- | MentalWLFac | ,378 |
| MSD Shoulders | <--- | Age | -,043 |
| MSD Shoulders | <--- | Ratio Pauses Hour | -,013 |
| MSD Shoulders | <--- | Daily time Computer use | ,047 |
| MSD Shoulders | <--- | Adequate furniture | ,029 |
| MSD Shoulders | <--- | Adequate ICT devices | -,106 |
| MSD Shoulders | <--- | Intense Physical activity | ,003 |

**Means: (Women - Default model)**

| | Estimate | S.E. | C.R. | P | Label |
|---|---|---|---|---|---|
| **Daily time Computer use** | 5,791 | ,084 | 68,910 | *** | par_66 |
| **Ratio Pauses Hour** | ,514 | ,023 | 22,586 | *** | par_68 |
| **Age** | 42,925 | ,324 | 132,541 | *** | par_69 |
| **Adequate ICT devices** | ,600 | ,019 | 31,676 | *** | par_70 |
| **Adequate furniture** | ,686 | ,018 | 38,275 | *** | par_71 |
| **Intense Physical activity** | 2,674 | ,098 | 27,229 | *** | par_72 |

**Intercepts: (Women - Default model)**

| | Estimate | S.E. | C.R. | P | Label |
|---|---|---|---|---|---|
| **MentalWLFac** | -,160 | ,268 | -,595 | ,552 | par_65 |
| **MSD Shoulders** | 5,124 | ,717 | 7,146 | *** | par_67 |

**Covariances: (Women - Default model)**

| | | | Estimate | S.E. | C.R. | P | Label |
|---|---|---|---|---|---|---|---|
| Adequate furniture | <--> | Adequate ICT devices | ,100 | ,010 | 10,417 | *** | par_48 |
| Adequate ICT devices | <--> | Daily time Computer use | ,005 | ,041 | ,132 | ,895 | par_49 |
| Adequate furniture | <--> | Daily time Computer use | ,077 | ,039 | 1,960 | ,050 | par_50 |
| Adequate furniture | <--> | Ratio Pauses Hour | ,000 | ,011 | -,046 | ,963 | par_51 |
| Ratio Pauses Hour | <--> | Daily time Computer use | -,551 | ,057 | -9,703 | *** | par_52 |
| Age | <--> | Ratio Pauses Hour | ,202 | ,190 | 1,060 | ,289 | par_53 |
| Ratio Pauses Hour | <--> | Intense Physical activity | ,034 | ,058 | ,594 | ,552 | par_54 |
| Age | <--> | Daily time Computer use | -1,372 | ,706 | -1,942 | ,052 | par_55 |
| Age | <--> | Adequate furniture | -,056 | ,151 | -,374 | ,708 | par_56 |
| Age | <--> | Adequate ICT devices | -,288 | ,160 | -1,803 | ,071 | par_57 |
| Age | <--> | Intense Physical activity | -,665 | ,823 | -,808 | ,419 | par_58 |
| Daily time Computer use | <--> | Intense Physical activity | -,160 | ,214 | -,751 | ,453 | par_59 |
| Adequate furniture | <--> | Intense Physical activity | ,036 | ,046 | ,784 | ,433 | par_60 |
| Adequate ICT devices | <--> | Intense Physical activity | ,091 | ,048 | 1,884 | ,060 | par_61 |
| Adequate ICT devices | <--> | Ratio Pauses Hour | ,023 | ,011 | 2,040 | ,041 | par_62 |

**Correlations: (Women - Default model)**

| | | | Estimate |
|---|---|---|---|
| Adequate furniture | <--> | Adequate ICT devices | ,440 |
| Adequate ICT devices | <--> | Daily time Computer use | ,005 |
| Adequate furniture | <--> | Daily time Computer use | ,076 |
| Adequate furniture | <--> | Ratio Pauses Hour | -,002 |
| Ratio Pauses Hour | <--> | Daily time Computer use | -,434 |
| Age | <--> | Ratio Pauses Hour | ,041 |
| Ratio Pauses Hour | <--> | Intense Physical activity | ,023 |
| Age | <--> | Daily time Computer use | -,075 |
| Age | <--> | Adequate furniture | -,015 |
| Age | <--> | Adequate ICT devices | -,070 |
| Age | <--> | Intense Physical activity | -,031 |
| Daily time Computer use | <--> | Intense Physical activity | -,029 |
| Adequate furniture | <--> | Intense Physical activity | ,030 |
| Adequate ICT devices | <--> | Intense Physical activity | ,073 |
| Adequate ICT devices | <--> | Ratio Pauses Hour | ,080 |

**Variances: (Women - Default model)**

| | Estimate | S.E. | C.R. | P | Label |
|---|---|---|---|---|---|
| **Age** | 70,263 | 3,839 | 18,301 | *** | par_81 |
| **Adequate furniture** | ,215 | ,012 | 18,288 | *** | par_82 |
| **Adequate ICT devices** | ,240 | ,013 | 18,289 | *** | par_83 |

| | Estimate | S.E. | C.R. | P | Label |
|---|---|---|---|---|---|
| **Ratio Pauses Hour** | ,340 | ,019 | 17,651 | *** | par_84 |
| **Daily time Computer use** | 4,731 | ,259 | 18,301 | *** | par_85 |
| **Intense Physical activity** | 6,458 | ,353 | 18,301 | *** | par_86 |
| **eMWL** | ,966 | ,056 | 17,332 | *** | par_87 |
| **eMSD** | 7,471 | ,415 | 17,997 | *** | par_88 |

**Squared Multiple Correlations: (Women - Default model)**

| | Estimate |
|---|---|
| **MentalWLFac** | ,108 |
| **MSD Shoulders** | ,178 |

**Matrices (Women - Default model)**

**Implied (for all variables) Covariances (Women - Default model)**

| | Intense Physical activity | Daily time Computer use | Ratio Pauses Hour | Adequate ICT devices | Adequate furniture | Age | MentalWLFac | MSD Shoulders |
|---|---|---|---|---|---|---|---|---|
| **Intense Physical activity** | 6,458 | | | | | | | |
| **Daily time Computer use** | -,160 | 4,731 | | | | | | |
| **Ratio Pauses Hour** | ,034 | -,551 | ,340 | | | | | |
| **Adequate ICT devices** | ,091 | ,005 | ,023 | ,240 | | | | |
| **Adequate furniture** | ,036 | ,077 | ,000 | ,100 | ,215 | | | |
| **Age** | -,665 | -1,372 | ,202 | -,288 | -,056 | 70,263 | | |
| **MentalWLFac** | ,032 | ,512 | -,150 | -,084 | -,055 | ,069 | 1,082 | |
| **MSD Shoulders** | ,001 | ,941 | -,241 | -,226 | -,078 | -,943 | 1,273 | 9,091 |

**Implied (for all variables) Correlations (Women - Default model)**

| | Intense Physical activity | Daily time Computer use | Ratio Pauses Hour | Adequate ICT devices | Adequate furniture | Age | MentalWLFac | MSD Shoulders |
|---|---|---|---|---|---|---|---|---|
| **Intense Physical activity** | 1,000 | | | | | | | |
| **Daily time Computer use** | -,029 | 1,000 | | | | | | |
| **Ratio Pauses Hour** | ,023 | -,434 | 1,000 | | | | | |
| **Adequate ICT devices** | ,073 | ,005 | ,080 | 1,000 | | | | |
| **Adequate furniture** | ,030 | ,076 | -,002 | ,440 | 1,000 | | | |
| **Age** | -,031 | -,075 | ,041 | -,070 | -,015 | 1,000 | | |
| **MentalWLFac** | ,012 | ,226 | -,248 | -,166 | -,114 | ,008 | 1,000 | |
| **MSD Shoulders** | ,000 | ,143 | -,137 | -,153 | -,056 | -,037 | ,406 | 1,000 |

**Implied (for all variables) Means (Women - Default model)**

| | Intense Physical activity | Daily time Computer use | Ratio Pauses Hour | Adequate ICT devices | Adequate furniture | Age | MentalWLFac | MSD Shoulders |
|---|---|---|---|---|---|---|---|---|
| | 2,674 | 5,791 | ,514 | ,600 | ,686 | 42,925 | -,003 | 4,551 |

**Implied Covariances (Women - Default model)**

| | Intense Physical activity | Daily time Computer use | Ratio Pauses Hour | Adequate ICT devices | Adequate furniture | Age | MentalWLFac | MSD Shoulders |
|---|---|---|---|---|---|---|---|---|
| **Intense Physical activity** | 6,458 | | | | | | | |
| **Daily time Computer use** | -,160 | 4,731 | | | | | | |
| **Ratio Pauses Hour** | ,034 | -,551 | ,340 | | | | | |
| **Adequate ICT devices** | ,091 | ,005 | ,023 | ,240 | | | | |
| **Adequate furniture** | ,036 | ,077 | ,000 | ,100 | ,215 | | | |
| **Age** | -,665 | -1,372 | ,202 | -,288 | -,056 | 70,263 | | |
| **MentalWLFac** | ,032 | ,512 | -,150 | -,084 | -,055 | ,069 | 1,082 | |
| **MSD Shoulders** | ,001 | ,941 | -,241 | -,226 | -,078 | -,943 | 1,273 | 9,091 |

**Implied Correlations (Women - Default model)**

| | Intense Physical activity | Daily time Computer use | Ratio Pauses Hour | Adequate ICT devices | Adequate furniture | Age | MentalWLFac | MSD Shoulders |
|---|---|---|---|---|---|---|---|---|
| **Intense Physical activity** | 1,000 | | | | | | | |
| **Daily time Computer use** | -,029 | 1,000 | | | | | | |
| **Ratio Pauses Hour** | ,023 | -,434 | 1,000 | | | | | |
| **Adequate ICT devices** | ,073 | ,005 | ,080 | 1,000 | | | | |
| **Adequate furniture** | ,030 | ,076 | -,002 | ,440 | 1,000 | | | |
| **Age** | -,031 | -,075 | ,041 | -,070 | -,015 | 1,000 | | |
| **MentalWLFac** | ,012 | ,226 | -,248 | -,166 | -,114 | ,008 | 1,000 | |
| **MSD Shoulders** | ,000 | ,143 | -,137 | -,153 | -,056 | -,037 | ,406 | 1,000 |

**Implied Means (Women - Default model)**

| | Intense Physical activity | Daily time Computer use | Ratio Pauses Hour | Adequate ICT devices | Adequate furniture | Age | MentalWLFac | MSD Shoulders |
|---|---|---|---|---|---|---|---|---|
| | 2,674 | 5,791 | ,514 | ,600 | ,686 | 42,925 | -,003 | 4,551 |

**Factor Score Weights (Women - Default model)**

¤

**Total Effects (Women - Default model)**

| | Intense Physical activity | Daily time Computer use | Ratio Pauses Hour | Adequate ICT devices | Adequate furniture | Age | MentalWLFac |
|---|---|---|---|---|---|---|---|
| **MentalWLFac** | ,013 | ,077 | -,302 | -,257 | -,167 | ,002 | ,000 |
| **MSD Shoulders** | ,018 | ,150 | -,397 | -,933 | ,008 | -,013 | 1,096 |

**Standardized Total Effects (Women - Default model)**

| | Intense Physical activity | Daily time Computer use | Ratio Pauses Hour | Adequate ICT devices | Adequate furniture | Age | MentalWLFac |
|---|---|---|---|---|---|---|---|
| **MentalWLFac** | ,032 | ,162 | -,169 | -,121 | -,074 | ,018 | ,000 |
| **MSD Shoulders** | ,015 | ,109 | -,077 | -,152 | ,001 | -,036 | ,378 |

**Direct Effects (Women - Default model)**

| | Intense Physical activity | Daily time Computer use | Ratio Pauses Hour | Adequate ICT devices | Adequate furniture | Age | MentalWLFac |
|---|---|---|---|---|---|---|---|
| **MentalWLFac** | ,013 | ,077 | -,302 | -,257 | -,167 | ,002 | ,000 |
| **MSD Shoulders** | ,003 | ,066 | -,066 | -,650 | ,191 | -,015 | 1,096 |

**Standardized Direct Effects (Women - Default model)**

| | Intense Physical activity | Daily time Computer use | Ratio Pauses Hour | Adequate ICT devices | Adequate furniture | Age | MentalWLFac |
|---|---|---|---|---|---|---|---|
| **MentalWLFac** | ,032 | ,162 | -,169 | -,121 | -,074 | ,018 | ,000 |
| **MSD Shoulders** | ,003 | ,047 | -,013 | -,106 | ,029 | -,043 | ,378 |

**Indirect Effects (Women - Default model)**

| | Intense Physical activity | Daily time Computer use | Ratio Pauses Hour | Adequate ICT devices | Adequate furniture | Age | MentalWLFac |
|---|---|---|---|---|---|---|---|
| **MentalWLFac** | ,000 | ,000 | ,000 | ,000 | ,000 | ,000 | ,000 |
| **MSD Shoulders** | ,014 | ,085 | -,331 | -,282 | -,183 | ,003 | ,000 |

**Standardized Indirect Effects (Women - Default model)**

| | Intense Physical activity | Daily time Computer use | Ratio Pauses Hour | Adequate ICT devices | Adequate furniture | Age | MentalWLFac |
|---|---|---|---|---|---|---|---|
| **MentalWLFac** | ,000 | ,000 | ,000 | ,000 | ,000 | ,000 | ,000 |
| **MSD Shoulders** | ,012 | ,061 | -,064 | -,046 | -,028 | ,007 | ,000 |

**Minimization History (Default model)**

| Iteration | | Negative eigenvalues | Condition # | Smallest eigenvalue | Diameter | F | NTries | Ratio |
|---|---|---|---|---|---|---|---|---|
| **0** | e | 10 | | -,847 | 9999,000 | 44206,753 | 0 | 9999,000 |
| **1** | e | 9 | | -,234 | ,662 | 13092,901 | 12 | ,815 |
| **2** | e | 7 | | -,297 | 1,273 | 3335,212 | 8 | ,846 |
| **3** | e* | 2 | | -,210 | ,809 | 1461,242 | 5 | 1,007 |
| **4** | e | 0 | 71593,889 | | ,331 | 953,819 | 5 | ,948 |
| **5** | e | 0 | 52063,742 | | ,525 | 822,609 | 6 | ,000 |
| **6** | e | 0 | 50299,482 | | ,949 | 381,779 | 2 | ,000 |

| Iteration | | Negative eigenvalues | Condition # | Smallest eigenvalue | Diameter | F | NTries | Ratio |
|---|---|---|---|---|---|---|---|---|
| **7** | e | 0 | 51836,771 | | 1,116 | 56,023 | 1 | 1,187 |
| **8** | e | 0 | 48484,057 | | ,521 | 4,117 | 1 | 1,145 |
| **9** | e | 0 | 48764,001 | | ,207 | ,080 | 1 | 1,079 |
| **10** | e | 0 | 49969,286 | | ,037 | ,000 | 1 | 1,020 |
| **11** | e | 0 | 49479,425 | | ,001 | ,000 | 1 | 1,001 |
| **12** | e | 0 | 49479,414 | | ,000 | ,000 | 1 | 1,000 |

**Miscellaneous**

**Model Fit Summary**

**CMIN**

| Model | NPAR | CMIN | DF | P | CMIN/DF |
|---|---|---|---|---|---|
| **Default model** | 88 | ,000 | 0 | | |
| **Saturated model** | 88 | ,000 | 0 | | |
| **Independence model** | 16 | 828,408 | 72 | ,000 | 11,506 |

**Baseline Comparisons**

| Model | NFI Delta1 | RFI rho1 | IFI Delta2 | TLI rho2 | CFI |
|---|---|---|---|---|---|
| **Default model** | 1,000 | | 1,000 | | 1,000 |
| **Saturated model** | 1,000 | | 1,000 | | 1,000 |
| **Independence model** | ,000 | ,000 | ,000 | ,000 | ,000 |

**Parsimony-Adjusted Measures**

| Model | PRATIO | PNFI | PCFI |
|---|---|---|---|
| **Default model** | ,000 | ,000 | ,000 |
| **Saturated model** | ,000 | ,000 | ,000 |
| **Independence model** | 1,000 | ,000 | ,000 |

**NCP**

| Model | NCP | LO 90 | HI 90 |
|---|---|---|---|
| **Default model** | ,000 | ,000 | ,000 |
| **Saturated model** | ,000 | ,000 | ,000 |
| **Independence model** | 756,408 | 667,485 | 852,770 |

**FMIN**

| Model | FMIN | F0 | LO 90 | HI 90 |
|---|---|---|---|---|
| Default model | ,000 | ,000 | ,000 | ,000 |
| Saturated model | ,000 | ,000 | ,000 | ,000 |
| Independence model | ,693 | ,632 | ,558 | ,713 |

**RMSEA**

| Model | RMSEA | LO 90 | HI 90 | PCLOSE |
|---|---|---|---|---|
| Independence model | ,094 | ,088 | ,100 | ,000 |

**AIC**

| Model | AIC | BCC | BIC | CAIC |
|---|---|---|---|---|
| Default model | 176,000 | 178,735 | | |
| Saturated model | 176,000 | 178,735 | | |
| Independence model | 860,408 | 860,905 | | |

**ECVI**

| Model | ECVI | LO 90 | HI 90 | MECVI |
|---|---|---|---|---|
| Default model | ,147 | ,147 | ,147 | ,149 |
| Saturated model | ,147 | ,147 | ,147 | ,149 |
| Independence model | ,719 | ,645 | ,800 | ,720 |

**HOELTER**

| Model | HOELTER .05 | HOELTER .01 |
|---|---|---|
| Default model | | |
| Independence model | 135 | 150 |

**Execution time summary**

| | |
|---|---|
| **Minimization:** | ,032 |
| **Miscellaneous:** | ,495 |
| **Bootstrap:** | ,000 |
| **Total:** | ,527 |

# 3.MSD- UPPER BACK

**Group number 1 (Group number 1 = Men )**

**Notes for Group (Group number 1 = Men )**

The model is recursive.
Sample size = 527

**Variable Summary (Men)**

**Your model contains the following variables (Men)**

Observed, endogenous variables
MSD-Upper back
MentalWLFac
Observed, exogenous variables
Age
Adequate furniture
Adequate ICT devices
Ratio Pauses Hour
Daily time Computer use
Intense Physical activity
Unobserved, exogenous variables
eMSD
eMWL

**Variable counts (Men)**

| | |
|---|---|
| **Number of variables in your model:** | 10 |
| **Number of observed variables:** | 8 |
| **Number of unobserved variables:** | 2 |
| **Number of exogenous variables:** | 8 |
| **Number of endogenous variables:** | 2 |

**Parameter Summary (Men)**

| | Weights | Covariances | Variances | Means | Intercepts | Total |
|---|---|---|---|---|---|---|
| **Fixed** | 2 | 0 | 0 | 2 | 0 | 4 |
| **Labeled** | 0 | 0 | 0 | 0 | 0 | 0 |
| **Unlabeled** | 13 | 15 | 8 | 6 | 2 | 44 |
| **Total** | 15 | 15 | 8 | 8 | 2 | 48 |

**Group number 2 (Group number 2= Women)**

**Notes for Group (Group number 2=Women)**

The model is recursive.
Sample size = 671

**Variable Summary (Women)**

**Your model contains the following variables (Women)**

Observed, endogenous variables
MSD-Upper back
MentalWLFac
Observed, exogenous variables
Age
Adequate furniture
Adequate ICT devices
Ratio Pauses Hour
Daily time Computer use
Intense Physical activity
Unobserved, exogenous variables
eMSD
eMWL

**Variable counts (Women)**

| | |
|---|---|
| **Number of variables in your model:** | 10 |
| **Number of observed variables:** | 8 |
| **Number of unobserved variables:** | 2 |
| **Number of exogenous variables:** | 8 |
| **Number of endogenous variables:** | 2 |

**Parameter Summary (Women)**

| | Weights | Covariances | Variances | Means | Intercepts | Total |
|---|---|---|---|---|---|---|
| **Fixed** | 2 | 0 | 0 | 2 | 0 | 4 |
| **Labeled** | 0 | 0 | 0 | 0 | 0 | 0 |
| **Unlabeled** | 13 | 15 | 8 | 6 | 2 | 44 |
| **Total** | 15 | 15 | 8 | 8 | 2 | 48 |

**Models**

**Default model (Default model)**

**Notes for Model (Default model)**

**Computation of degrees of freedom (Default model)**

| | |
|---|---|
| **Number of distinct sample moments:** | 88 |
| **Number of distinct parameters to be estimated:** | 88 |
| **Degrees of freedom (88 – 88):** | 0 |

**Result (Default model)**

Minimum was achieved
Chi-square = ,000
Degrees of freedom = 0
Probability level cannot be computed

**Men (Men – Default model)**

**Estimates (Men – Default model)**

**Scalar Estimates (Men – Default model)**

**Maximum Likelihood Estimates**

**Regression Weights: (Men – Default model)**

| | | | Estimate | S.E. | C.R. | P | Label |
|---|---|---|---|---|---|---|---|
| MentalWLFac | ←- | Age | -,008 | ,005 | -1,607 | ,108 | par_2 |
| MentalWLFac | ←- | Adequate furniture | -,110 | ,097 | -1,129 | ,259 | par_4 |
| MentalWLFac | ←- | Adequate ICT devices | -,158 | ,094 | -1,677 | ,094 | par_5 |
| MentalWLFac | ←- | Daily time Computer use | ,078 | ,018 | 4,394 | *** | par_10 |
| MentalWLFac | ←- | Ratio Pauses Hour | -,046 | ,055 | -,846 | ,397 | par_11 |
| MentalWLFac | ←- | Intense Physical activity | ,017 | ,014 | 1,271 | ,204 | par_27 |
| MSD-Upper back | ←- | MentalWLFac | 1,189 | ,125 | 9,523 | *** | par_1 |
| MSD-Upper back | ←- | Age | ,000 | ,013 | ,038 | ,970 | par_3 |
| MSD-Upper back | ←- | Ratio Pauses Hour | ,094 | ,139 | ,679 | ,497 | par_6 |
| MSD-Upper back | ←- | Daily time Computer use | -,022 | ,049 | -,445 | ,656 | par_7 |
| MSD-Upper back | ←- | Adequate furniture | -,172 | ,271 | -,636 | ,525 | par_8 |
| MSD-Upper back | ←- | Adequate ICT devices | -,294 | ,263 | -1,118 | ,263 | par_9 |
| MSD-Upper back | ←- | Intense Physical activity | ,012 | ,037 | ,333 | ,739 | par_28 |

**Standardized Regression Weights: (Men – Default model)**

| | | | Estimate |
|---|---|---|---|
| MentalWLFac | <- | Age | -,069 |
| MentalWLFac | <- | Adequate furniture | -,055 |
| MentalWLFac | <- | Adequate ICT devices | -,080 |
| MentalWLFac | <- | Daily time Computer use | ,218 |
| MentalWLFac | <- | Ratio Pauses Hour | -,045 |
| MentalWLFac | <- | Intense Physical activity | ,055 |
| MSD-Upper back | <- | MentalWLFac | ,401 |
| MSD-Upper back | <- | Age | ,002 |
| MSD-Upper back | <- | Ratio Pauses Hour | ,031 |
| MSD-Upper back | <- | Daily time Computer use | -,021 |
| MSD-Upper back | <- | Adequate furniture | -,029 |
| MSD-Upper back | <- | Adequate ICT devices | -,050 |
| MSD-Upper back | <- | Intense Physical activity | ,013 |

**Means: (Men – Default model)**

| | Estimate | S.E. | C.R. | P | Label |
|---|---|---|---|---|---|
| **Daily time Computer use** | 4,780 | ,116 | 41,033 | *** | par_30 |
| **Ratio Pauses Hour** | ,859 | ,043 | 19,891 | *** | par_32 |
| **Age** | 45,004 | ,383 | 117,515 | *** | par_33 |
| **Adequate ICT devices** | ,626 | ,021 | 29,590 | *** | par_34 |
| **Adequate furniture** | ,656 | ,021 | 31,618 | *** | par_35 |
| **Intense Physical activity** | 4,304 | ,133 | 32,348 | *** | par_36 |

**Intercepts: (Men – Default model)**

| | Estimate | S.E. | C.R. | P | Label |
|---|---|---|---|---|---|
| **MentalWLFac** | ,099 | ,265 | ,375 | ,708 | par_29 |
| **MSD-Upper back** | 4,475 | ,720 | 6,211 | *** | par_31 |

**Covariances: (Men – Default model)**

| | | | Estimate | S.E. | C.R. | P | Label |
|---|---|---|---|---|---|---|---|
| Adequate furniture | <-> | Adequate ICT devices | ,102 | ,011 | 9,313 | *** | par_12 |
| Adequate ICT devices | <-> | Daily time Computer use | ,053 | ,057 | ,930 | ,352 | par_13 |
| Adequate furniture | <-> | Daily time Computer use | ,230 | ,056 | 4,088 | *** | par_14 |
| Adequate furniture | <-> | Ratio Pauses Hour | -,045 | ,021 | -2,152 | ,031 | par_15 |
| Ratio Pauses Hour | <-> | Daily time Computer use | -1,094 | ,137 | -8,010 | *** | par_16 |
| Age | <-> | Ratio Pauses Hour | ,083 | ,371 | ,224 | ,822 | par_17 |
| Ratio Pauses Hour | <-> | Intense Physical activity | ,057 | ,130 | ,436 | ,663 | par_18 |
| Age | <-> | Daily time Computer use | -,094 | 1,023 | -,092 | ,927 | par_19 |

| | | | Estimate | S.E. | C.R. | P | Label |
|---|---|---|---|---|---|---|---|
| Age | <-> | Adequate furniture | -,009 | ,182 | -,048 | ,962 | par_20 |
| Age | <-> | Adequate ICT devices | -,326 | ,186 | -1,753 | ,080 | par_21 |
| Age | <-> | Intense Physical activity | -2,857 | 1,175 | -2,431 | ,015 | par_22 |
| Daily time Computer use | <-> | Intense Physical activity | -,233 | ,356 | -,655 | ,512 | par_23 |
| Adequate furniture | <-> | Intense Physical activity | ,010 | ,063 | ,165 | ,869 | par_24 |
| Adequate ICT devices | <-> | Intense Physical activity | ,093 | ,065 | 1,437 | ,151 | par_25 |
| Adequate ICT devices | <-> | Ratio Pauses Hour | -,001 | ,021 | -,027 | ,978 | par_26 |

**Correlations: (Men – Default model)**

| | | | Estimate |
|---|---|---|---|
| Adequate furniture | <-> | Adequate ICT devices | ,446 |
| Adequate ICT devices | <-> | Daily time Computer use | ,041 |
| Adequate furniture | <-> | Daily time Computer use | ,181 |
| Adequate furniture | <-> | Ratio Pauses Hour | -,101 |
| Ratio Pauses Hour | <-> | Daily time Computer use | -,437 |
| Age | <-> | Ratio Pauses Hour | ,010 |
| Ratio Pauses Hour | <-> | Intense Physical activity | ,020 |
| Age | <-> | Daily time Computer use | -,004 |
| Age | <-> | Adequate furniture | -,002 |
| Age | <-> | Adequate ICT devices | -,077 |
| Age | <-> | Intense Physical activity | -,107 |
| Daily time Computer use | <-> | Intense Physical activity | -,029 |
| Adequate furniture | <-> | Intense Physical activity | ,007 |
| Adequate ICT devices | <-> | Intense Physical activity | ,063 |
| Adequate ICT devices | <-> | Ratio Pauses Hour | -,001 |

**Variances: (Men – Default model)**

| | Estimate | S.E. | C.R. | P | Label |
|---|---|---|---|---|---|
| **Age** | 77,161 | 4,757 | 16,219 | *** | par_73 |
| **Adequate furniture** | ,225 | ,014 | 16,176 | *** | par_74 |
| **Adequate ICT devices** | ,234 | ,014 | 16,174 | *** | par_75 |
| **Ratio Pauses Hour** | ,879 | ,060 | 14,704 | *** | par_76 |
| **Daily time Computer use** | 7,139 | ,440 | 16,219 | *** | par_77 |
| **Intense Physical activity** | 9,312 | ,574 | 16,219 | *** | par_78 |
| **eMWL** | ,843 | ,053 | 15,868 | *** | par_79 |
| **eMSD** | 6,658 | ,414 | 16,089 | *** | par_80 |

**Squared Multiple Correlations: (Men – Default model)**

| | Estimate |
|---|---|
| **MentalWLFac** | ,072 |
| **MSD-Upper back** | ,166 |

**Matrices (Men – Default model)**

**Implied (for all variables) Covariances (Men – Default model)**

| | Intense Physical activity | Daily time Computer use | Ratio Pauses Hour | Adequate ICT devices | Adequate furniture | Age | MentalWLFac | MSD-Upper back |
|---|---|---|---|---|---|---|---|---|
| Intense Physical activity | 9,312 | | | | | | | |
| Daily time Computer use | -,233 | 7,139 | | | | | | |
| Ratio Pauses Hour | ,057 | -1,094 | ,879 | | | | | |
| Adequate ICT devices | ,093 | ,053 | -,001 | ,234 | | | | |
| Adequate furniture | ,010 | ,230 | -,045 | ,102 | ,225 | | | |
| Age | -2,857 | -,094 | ,083 | -,326 | -,009 | 77,161 | | |
| MentalWLFac | ,145 | ,568 | -,120 | -,040 | -,021 | -,587 | ,908 | |
| MSD-Upper back | ,268 | ,359 | -,028 | -,135 | -,103 | -,589 | 1,073 | 7,984 |

**Implied (for all variables) Correlations (Men – Default model)**

| | Intense Physical activity | Daily time Computer use | Ratio Pauses Hour | Adequate ICT devices | Adequate furniture | Age | MentalWLFac | MSD-Upper back |
|---|---|---|---|---|---|---|---|---|
| Intense Physical activity | 1,000 | | | | | | | |
| Daily time Computer use | -,029 | 1,000 | | | | | | |
| Ratio Pauses Hour | ,020 | -,437 | 1,000 | | | | | |
| Adequate ICT devices | ,063 | ,041 | -,001 | 1,000 | | | | |
| Adequate furniture | ,007 | ,181 | -,101 | ,446 | 1,000 | | | |
| Age | -,107 | -,004 | ,010 | -,077 | -,002 | 1,000 | | |
| MentalWLFac | ,050 | ,223 | -,134 | -,087 | -,046 | -,070 | 1,000 | |
| MSD-Upper back | ,031 | ,047 | -,010 | -,098 | -,077 | -,024 | ,399 | 1,000 |

**Implied (for all variables) Means (Men – Default model)**

| | Intense Physical activity | Daily time Computer use | Ratio Pauses Hour | Adequate ICT devices | Adequate furniture | Age | MentalWLFac | MSD-Upper back |
|---|---|---|---|---|---|---|---|---|
| | 4,304 | 4,780 | ,859 | ,626 | ,656 | 45,004 | -,005 | 4,224 |

**Implied Covariances (Men – Default model)**

| | Intense Physical activity | Daily time Computer use | Ratio Pauses Hour | Adequate ICT devices | Adequate furniture | Age | MentalWLFac | MSD-Upper back |
|---|---|---|---|---|---|---|---|---|
| Intense Physical activity | 9,312 | | | | | | | |
| Daily time Computer use | -,233 | 7,139 | | | | | | |
| Ratio Pauses Hour | ,057 | -1,094 | ,879 | | | | | |

| | Intense Physical activity | Daily time Computer use | Ratio Pauses Hour | Adequate ICT devices | Adequate furniture | Age | MentalWLFac | MSD-Upper back |
|---|---|---|---|---|---|---|---|---|
| Adequate ICT devices | ,093 | ,053 | -,001 | ,234 | | | | |
| Adequate furniture | ,010 | ,230 | -,045 | ,102 | ,225 | | | |
| Age | -2,857 | -,094 | ,083 | -,326 | -,009 | 77,161 | | |
| MentalWLFac | ,145 | ,568 | -,120 | -,040 | -,021 | -,587 | ,908 | |
| MSD-Upper back | ,268 | ,359 | -,028 | -,135 | -,103 | -,589 | 1,073 | 7,984 |

**Implied Correlations (Men – Default model)**

| | Intense Physical activity | Daily time Computer use | Ratio Pauses Hour | Adequate ICT devices | Adequate furniture | Age | MentalWLFac | MSD-Upper back |
|---|---|---|---|---|---|---|---|---|
| Intense Physical activity | 1,000 | | | | | | | |
| Daily time Computer use | -,029 | 1,000 | | | | | | |
| Ratio Pauses Hour | ,020 | -,437 | 1,000 | | | | | |
| Adequate ICT devices | ,063 | ,041 | -,001 | 1,000 | | | | |
| Adequate furniture | ,007 | ,181 | -,101 | ,446 | 1,000 | | | |
| Age | -,107 | -,004 | ,010 | -,077 | -,002 | 1,000 | | |
| MentalWLFac | ,050 | ,223 | -,134 | -,087 | -,046 | -,070 | 1,000 | |
| MSD-Upper back | ,031 | ,047 | -,010 | -,098 | -,077 | -,024 | ,399 | 1,000 |

**Implied Means (Men – Default model)**

| | Intense Physical activity | Daily time Computer use | Ratio Pauses Hour | Adequate ICT devices | Adequate furniture | Age | MentalWLFac | MSD-Upper back |
|---|---|---|---|---|---|---|---|---|
| | 4,304 | 4,780 | ,859 | ,626 | ,656 | 45,004 | -,005 | 4,224 |

**Factor Score Weights (Men – Default model)**

**Total Effects (Men – Default model)**

| | Intense Physical activity | Daily time Computer use | Ratio Pauses Hour | Adequate ICT devices | Adequate furniture | Age | MentalWLFac |
|---|---|---|---|---|---|---|---|
| MentalWLFac | ,017 | ,078 | -,046 | -,158 | -,110 | -,008 | ,000 |
| MSD-Upper back | ,033 | ,071 | ,039 | -,483 | -,303 | -,008 | 1,189 |

**Standardized Total Effects (Men – Default model)**

| | Intense Physical activity | Daily time Computer use | Ratio Pauses Hour | Adequate ICT devices | Adequate furniture | Age | MentalWLFac |
|---|---|---|---|---|---|---|---|
| MentalWLFac | ,055 | ,218 | -,045 | -,080 | -,055 | -,069 | ,000 |
| MSD-Upper back | ,035 | ,067 | ,013 | -,083 | -,051 | -,026 | ,401 |

**Direct Effects (Men – Default model)**

| | Intense Physical activity | Daily time Computer use | Ratio Pauses Hour | Adequate ICT devices | Adequate furniture | Age | MentalWLFac |
|---|---|---|---|---|---|---|---|
| **MentalWLFac** | ,017 | ,078 | -,046 | -,158 | -,110 | -,008 | ,000 |
| **MSD-Upper back** | ,012 | -,022 | ,094 | -,294 | -,172 | ,000 | 1,189 |

**Standardized Direct Effects (Men – Default model)**

| | Intense Physical activity | Daily time Computer use | Ratio Pauses Hour | Adequate ICT devices | Adequate furniture | Age | MentalWLFac |
|---|---|---|---|---|---|---|---|
| **MentalWLFac** | ,055 | ,218 | -,045 | -,080 | -,055 | -,069 | ,000 |
| **MSD-Upper back** | ,013 | -,021 | ,031 | -,050 | -,029 | ,002 | ,401 |

**Indirect Effects (Men – Default model)**

| | Intense Physical activity | Daily time Computer use | Ratio Pauses Hour | Adequate ICT devices | Adequate furniture | Age | MentalWLFac |
|---|---|---|---|---|---|---|---|
| **MentalWLFac** | ,000 | ,000 | ,000 | ,000 | ,000 | ,000 | ,000 |
| **MSD-Upper back** | ,020 | ,092 | -,055 | -,188 | -,131 | -,009 | ,000 |

**Standardized Indirect Effects (Men – Default model)**

| | Intense Physical activity | Daily time Computer use | Ratio Pauses Hour | Adequate ICT devices | Adequate furniture | Age | MentalWLFac |
|---|---|---|---|---|---|---|---|
| **MentalWLFac** | ,000 | ,000 | ,000 | ,000 | ,000 | ,000 | ,000 |
| **MSD-Upper back** | ,022 | ,087 | -,018 | -,032 | -,022 | -,028 | ,000 |

**Women (Women – Default model)**

**Estimates (Women – Default model)**

**Scalar Estimates (Women – Default model)**

**Maximum Likelihood Estimates**

**Regression Weights: (Women – Default model)**

| | | | Estimate | S.E. | C.R. | P | Label |
|---|---|---|---|---|---|---|---|
| MentalWLFac | ←- | Age | ,002 | ,005 | ,344 | ,731 | par_38 |
| MentalWLFac | ←- | Adequate furniture | -,156 | ,096 | -1,626 | ,104 | par_40 |
| MentalWLFac | ←- | Adequate ICT devices | -,263 | ,091 | -2,883 | ,004 | par_41 |
| MentalWLFac | ←- | Daily time Computer use | ,073 | ,021 | 3,528 | *** | par_46 |
| MentalWLFac | ←- | Ratio Pauses Hour | -,305 | ,077 | -3,961 | *** | par_47 |
| MentalWLFac | ←- | Intense Physical activity | ,013 | ,016 | ,863 | ,388 | par_63 |
| MSD-Upper back | ←- | MentalWLFac | 1,132 | ,114 | 9,975 | *** | par_37 |
| MSD-Upper back | ←- | Age | -,042 | ,013 | -3,279 | ,001 | par_39 |

| | | | Estimate | S.E. | C.R. | P | Label |
|---|---|---|---|---|---|---|---|
| MSD-Upper back | ←- | Ratio Pauses Hour | ,231 | ,210 | 1,100 | ,271 | par_42 |
| MSD-Upper back | ←- | Daily time Computer use | ,117 | ,056 | 2,103 | ,035 | par_43 |
| MSD-Upper back | ←- | Adequate furniture | -,222 | ,258 | -,858 | ,391 | par_44 |
| MSD-Upper back | ←- | Adequate ICT devices | -,407 | ,247 | -1,648 | ,099 | par_45 |
| MSD-Upper back | ←- | Intense Physical activity | ,000 | ,042 | ,001 | ,999 | par_64 |

**Standardized Regression Weights: (Women – Default model)**

| | | | Estimate |
|---|---|---|---|
| MentalWLFac | ←- | Age | ,013 |
| MentalWLFac | ←- | Adequate furniture | -,069 |
| MentalWLFac | ←- | Adequate ICT devices | -,124 |
| MentalWLFac | ←- | Daily time Computer use | ,152 |
| MentalWLFac | ←- | Ratio Pauses Hour | -,171 |
| MentalWLFac | ←- | Intense Physical activity | ,033 |
| MSD-Upper back | ←- | MentalWLFac | ,386 |
| MSD-Upper back | ←- | Age | -,116 |
| MSD-Upper back | ←- | Ratio Pauses Hour | ,044 |
| MSD-Upper back | ←- | Daily time Computer use | ,083 |
| MSD-Upper back | ←- | Adequate furniture | -,034 |
| MSD-Upper back | ←- | Adequate ICT devices | -,065 |
| MSD-Upper back | ←- | Intense Physical activity | ,000 |

**Means: (Women – Default model)**

| | Estimate | S.E. | C.R. | P | Label |
|---|---|---|---|---|---|
| **Daily time Computer use** | 5,791 | ,084 | 68,910 | *** | par_66 |
| **Ratio Pauses Hour** | ,514 | ,023 | 22,587 | *** | par_68 |
| **Age** | 42,925 | ,324 | 132,541 | *** | par_69 |
| **Adequate ICT devices** | ,600 | ,019 | 31,672 | *** | par_70 |
| **Adequate furniture** | ,686 | ,018 | 38,271 | *** | par_71 |
| **Intense Physical activity** | 2,674 | ,098 | 27,229 | *** | par_72 |

**Intercepts: (Women – Default model)**

| | Estimate | S.E. | C.R. | P | Label |
|---|---|---|---|---|---|
| **MentalWLFac** | -,111 | ,269 | -,412 | ,680 | par_65 |
| **MSD-Upper back** | 7,018 | ,721 | 9,734 | *** | par_67 |

**Covariances: (Women – Default model)**

| | | | Estimate | S.E. | C.R. | P | Label |
|---|---|---|---|---|---|---|---|
| Adequate furniture | <-> | Adequate ICT devices | ,100 | ,010 | 10,417 | *** | par_48 |
| Adequate ICT devices | <-> | Daily time Computer use | ,006 | ,041 | ,139 | ,890 | par_49 |
| Adequate furniture | <-> | Daily time Computer use | ,077 | ,039 | 1,965 | ,049 | par_50 |
| Adequate furniture | <-> | Ratio Pauses Hour | -,001 | ,011 | -,069 | ,945 | par_51 |
| Ratio Pauses Hour | <-> | Daily time Computer use | -,552 | ,057 | -9,721 | *** | par_52 |
| Age | <-> | Ratio Pauses Hour | ,201 | ,190 | 1,054 | ,292 | par_53 |
| Ratio Pauses Hour | <-> | Intense Physical activity | ,033 | ,058 | ,576 | ,564 | par_54 |
| Age | <-> | Daily time Computer use | -1,372 | ,706 | -1,942 | ,052 | par_55 |
| Age | <-> | Adequate furniture | -,057 | ,151 | -,381 | ,703 | par_56 |
| Age | <-> | Adequate ICT devices | -,290 | ,160 | -1,813 | ,070 | par_57 |
| Age | <-> | Intense Physical activity | -,665 | ,823 | -,808 | ,419 | par_58 |
| Daily time Computer use | <-> | Intense Physical activity | -,160 | ,214 | -,751 | ,453 | par_59 |
| Adequate furniture | <-> | Intense Physical activity | ,036 | ,046 | ,783 | ,434 | par_60 |
| Adequate ICT devices | <-> | Intense Physical activity | ,091 | ,048 | 1,882 | ,060 | par_61 |
| Adequate ICT devices | <-> | Ratio Pauses Hour | ,023 | ,011 | 2,028 | ,043 | par_62 |

**Correlations: (Women – Default model)**

| | | | Estimate |
|---|---|---|---|
| Adequate furniture | <-> | Adequate ICT devices | ,440 |
| Adequate ICT devices | <-> | Daily time Computer use | ,005 |
| Adequate furniture | <-> | Daily time Computer use | ,076 |
| Adequate furniture | <-> | Ratio Pauses Hour | -,003 |
| Ratio Pauses Hour | <-> | Daily time Computer use | -,435 |
| Age | <-> | Ratio Pauses Hour | ,041 |
| Ratio Pauses Hour | <-> | Intense Physical activity | ,023 |
| Age | <-> | Daily time Computer use | -,075 |
| Age | <-> | Adequate furniture | -,015 |
| Age | <-> | Adequate ICT devices | -,071 |
| Age | <-> | Intense Physical activity | -,031 |
| Daily time Computer use | <-> | Intense Physical activity | -,029 |
| Adequate furniture | <-> | Intense Physical activity | ,030 |
| Adequate ICT devices | <-> | Intense Physical activity | ,073 |
| Adequate ICT devices | <-> | Ratio Pauses Hour | ,079 |

**Variances: (Women – Default model)**

| | Estimate | S.E. | C.R. | P | Label |
|---|---|---|---|---|---|
| **Age** | 70,263 | 3,839 | 18,301 | *** | par_81 |
| **Adequate furniture** | ,215 | ,012 | 18,288 | *** | par_82 |
| **Adequate ICT devices** | ,240 | ,013 | 18,289 | *** | par_83 |

| | Estimate | S.E. | C.R. | P | Label |
|---|---|---|---|---|---|
| **Ratio Pauses Hour** | ,340 | ,019 | 17,643 | *** | par_84 |
| **Daily time Computer use** | 4,731 | ,259 | 18,301 | *** | par_85 |
| **Intense Physical activity** | 6,458 | ,353 | 18,301 | *** | par_86 |
| **eMWL** | ,968 | ,056 | 17,295 | *** | par_87 |
| **eMSD** | 7,542 | ,420 | 17,942 | *** | par_88 |

**Squared Multiple Correlations: (Women – Default model)**

| | Estimate |
|---|---|
| **MentalWLFac** | ,104 |
| **MSD-Upper back** | ,191 |

**Matrices (Women – Default model)**

**Implied (for all variables) Covariances (Women – Default model)**

| | Intense Physical activity | Daily time Computer use | Ratio Pauses Hour | Adequate ICT devices | Adequate furniture | Age | MentalWLFac | MSD-Upper back |
|---|---|---|---|---|---|---|---|---|
| **Intense Physical activity** | 6,458 | | | | | | | |
| **Daily time Computer use** | -,160 | 4,731 | | | | | | |
| **Ratio Pauses Hour** | ,033 | -,552 | ,340 | | | | | |
| **Adequate ICT devices** | ,091 | ,006 | ,023 | ,240 | | | | |
| **Adequate furniture** | ,036 | ,077 | -,001 | ,100 | ,215 | | | |
| **Age** | -,665 | -1,372 | ,201 | -,290 | -,057 | 70,263 | | |
| **MentalWLFac** | ,034 | ,494 | -,149 | -,084 | -,054 | ,031 | 1,080 | |
| **MSD-Upper back** | ,011 | 1,023 | -,172 | -,197 | -,138 | -2,906 | 1,292 | 9,318 |

**Implied (for all variables) Correlations (Women – Default model)**

| | Intense Physical activity | Daily time Computer use | Ratio Pauses Hour | Adequate ICT devices | Adequate furniture | Age | MentalWLFac | MSD-Upper back |
|---|---|---|---|---|---|---|---|---|
| **Intense Physical activity** | 1,000 | | | | | | | |
| **Daily time Computer use** | -,029 | 1,000 | | | | | | |
| **Ratio Pauses Hour** | ,023 | -,435 | 1,000 | | | | | |
| **Adequate ICT devices** | ,073 | ,005 | ,079 | 1,000 | | | | |
| **Adequate furniture** | ,030 | ,076 | -,003 | ,440 | 1,000 | | | |
| **Age** | -,031 | -,075 | ,041 | -,071 | -,015 | 1,000 | | |
| **MentalWLFac** | ,013 | ,218 | -,245 | -,166 | -,111 | ,004 | 1,000 | |
| **MSD-Upper back** | ,001 | ,154 | -,097 | -,132 | -,097 | -,114 | ,407 | 1,000 |

**Implied (for all variables) Means (Women – Default model)**

| | Intense Physical activity | Daily time Computer use | Ratio Pauses Hour | Adequate ICT devices | Adequate furniture | Age | MentalWLFac | MSD-Upper back |
|---|---|---|---|---|---|---|---|---|
| | 2,674 | 5,791 | ,514 | ,600 | ,686 | 42,925 | -,006 | 5,605 |

**Implied Covariances (Women – Default model)**

| | Intense Physical activity | Daily time Computer use | Ratio Pauses Hour | Adequate ICT devices | Adequate furniture | Age | MentalWLFac | MSD-Upper back |
|---|---|---|---|---|---|---|---|---|
| **Intense Physical activity** | 6,458 | | | | | | | |
| **Daily time Computer use** | -,160 | 4,731 | | | | | | |
| **Ratio Pauses Hour** | ,033 | -,552 | ,340 | | | | | |
| **Adequate ICT devices** | ,091 | ,006 | ,023 | ,240 | | | | |
| **Adequate furniture** | ,036 | ,077 | -,001 | ,100 | ,215 | | | |
| **Age** | -,665 | -1,372 | ,201 | -,290 | -,057 | 70,263 | | |
| **MentalWLFac** | ,034 | ,494 | -,149 | -,084 | -,054 | ,031 | 1,080 | |
| **MSD-Upper back** | ,011 | 1,023 | -,172 | -,197 | -,138 | -2,906 | 1,292 | 9,318 |

**Implied Correlations (Women – Default model)**

| | Intense Physical activity | Daily time Computer use | Ratio Pauses Hour | Adequate ICT devices | Adequate furniture | Age | MentalWLFac | MSD-Upper back |
|---|---|---|---|---|---|---|---|---|
| **Intense Physical activity** | 1,000 | | | | | | | |
| **Daily time Computer use** | -,029 | 1,000 | | | | | | |
| **Ratio Pauses Hour** | ,023 | -,435 | 1,000 | | | | | |
| **Adequate ICT devices** | ,073 | ,005 | ,079 | 1,000 | | | | |
| **Adequate furniture** | ,030 | ,076 | -,003 | ,440 | 1,000 | | | |
| **Age** | -,031 | -,075 | ,041 | -,071 | -,015 | 1,000 | | |
| **MentalWLFac** | ,013 | ,218 | -,245 | -,166 | -,111 | ,004 | 1,000 | |
| **MSD-Upper back** | ,001 | ,154 | -,097 | -,132 | -,097 | -,114 | ,407 | 1,000 |

**Implied Means (Women – Default model)**

| | Intense Physical activity | Daily time Computer use | Ratio Pauses Hour | Adequate ICT devices | Adequate furniture | Age | MentalWLFac | MSD-Upper back |
|---|---|---|---|---|---|---|---|---|
| | 2,674 | 5,791 | ,514 | ,600 | ,686 | 42,925 | -,006 | 5,605 |

**Factor Score Weights (Women – Default model)**

¤

**Total Effects (Women – Default model)**

| | Intense Physical activity | Daily time Computer use | Ratio Pauses Hour | Adequate ICT devices | Adequate furniture | Age | MentalWLFac |
|---|---|---|---|---|---|---|---|
| **MentalWLFac** | ,013 | ,073 | -,305 | -,263 | -,156 | ,002 | ,000 |
| **MSD-Upper back** | ,015 | ,199 | -,115 | -,705 | -,398 | -,040 | 1,132 |

**Standardized Total Effects (Women – Default model)**

| | Intense Physical activity | Daily time Computer use | Ratio Pauses Hour | Adequate ICT devices | Adequate furniture | Age | MentalWLFac |
|---|---|---|---|---|---|---|---|
| **MentalWLFac** | ,033 | ,152 | -,171 | -,124 | -,069 | ,013 | ,000 |
| **MSD-Upper back** | ,013 | ,142 | -,022 | -,113 | -,060 | -,110 | ,386 |

**Direct Effects (Women – Default model)**

| | Intense Physical activity | Daily time Computer use | Ratio Pauses Hour | Adequate ICT devices | Adequate furniture | Age | MentalWLFac |
|---|---|---|---|---|---|---|---|
| **MentalWLFac** | ,013 | ,073 | -,305 | -,263 | -,156 | ,002 | ,000 |
| **MSD-Upper back** | ,000 | ,117 | ,231 | -,407 | -,222 | -,042 | 1,132 |

**Standardized Direct Effects (Women – Default model)**

| | Intense Physical activity | Daily time Computer use | Ratio Pauses Hour | Adequate ICT devices | Adequate furniture | Age | MentalWLFac |
|---|---|---|---|---|---|---|---|
| **MentalWLFac** | ,033 | ,152 | -,171 | -,124 | -,069 | ,013 | ,000 |
| **MSD-Upper back** | ,000 | ,083 | ,044 | -,065 | -,034 | -,116 | ,386 |

**Indirect Effects (Women – Default model)**

| | Intense Physical activity | Daily time Computer use | Ratio Pauses Hour | Adequate ICT devices | Adequate furniture | Age | MentalWLFac |
|---|---|---|---|---|---|---|---|
| **MentalWLFac** | ,000 | ,000 | ,000 | ,000 | ,000 | ,000 | ,000 |
| **MSD-Upper back** | ,015 | ,082 | -,345 | -,298 | -,176 | ,002 | ,000 |

**Standardized Indirect Effects (Women – Default model)**

| | Intense Physical activity | Daily time Computer use | Ratio Pauses Hour | Adequate ICT devices | Adequate furniture | Age | MentalWLFac |
|---|---|---|---|---|---|---|---|
| **MentalWLFac** | ,000 | ,000 | ,000 | ,000 | ,000 | ,000 | ,000 |
| **MSD-Upper back** | ,013 | ,059 | -,066 | -,048 | -,027 | ,005 | ,000 |

**Minimization History (Default model)**

| Iteration | | Negative eigenvalues | Condition # | Smallest eigenvalue | Diameter | F | Ntries | Ratio |
|---|---|---|---|---|---|---|---|---|
| **0** | e | 10 | | -,820 | 9999,000 | 43317,246 | 0 | 9999,000 |
| **1** | e | 9 | | -,234 | ,659 | 13126,758 | 12 | ,811 |
| **2** | e | 7 | | -,297 | 1,273 | 3373,424 | 8 | ,846 |
| **3** | e* | 2 | | -,210 | ,810 | 1490,138 | 5 | 1,008 |
| **4** | e | 0 | 70459,309 | | ,331 | 979,550 | 5 | ,948 |
| **5** | e | 0 | 46637,988 | | ,528 | 847,908 | 6 | ,000 |
| **6** | e | 0 | 45083,430 | | 1,016 | 391,242 | 2 | ,000 |

| Iteration | | Negative eigenvalues | Condition # | Smallest eigenvalue | Diameter | F | Ntries | Ratio |
|---|---|---|---|---|---|---|---|---|
| **7** | e | 0 | 43636,576 | | 1,198 | 56,824 | 1 | 1,185 |
| **8** | e | 0 | 43159,822 | | ,530 | 4,138 | 1 | 1,144 |
| **9** | e | 0 | 42894,960 | | ,208 | ,080 | 1 | 1,079 |
| **10** | e | 0 | 43210,629 | | ,037 | ,000 | 1 | 1,020 |
| **11** | e | 0 | 43186,300 | | ,001 | ,000 | 1 | 1,001 |
| **12** | e | 0 | 43186,302 | | ,000 | ,000 | 1 | 1,000 |

**Miscellaneous**

**Model Fit Summary**

**CMIN**

| Model | NPAR | CMIN | DF | P | CMIN/DF |
|---|---|---|---|---|---|
| **Default model** | 88 | ,000 | 0 | | |
| **Saturated model** | 88 | ,000 | 0 | | |
| **Independence model** | 16 | 837,209 | 72 | ,000 | 11,628 |

**Baseline Comparisons**

| Model | NFI Delta1 | RFI rho1 | IFI Delta2 | TLI rho2 | CFI |
|---|---|---|---|---|---|
| **Default model** | 1,000 | | 1,000 | | 1,000 |
| **Saturated model** | 1,000 | | 1,000 | | 1,000 |
| **Independence model** | ,000 | ,000 | ,000 | ,000 | ,000 |

**Parsimony-Adjusted Measures**

| Model | PRATIO | PNFI | PCFI |
|---|---|---|---|
| **Default model** | ,000 | ,000 | ,000 |
| **Saturated model** | ,000 | ,000 | ,000 |
| **Independence model** | 1,000 | ,000 | ,000 |

**NCP**

| Model | NCP | LO 90 | HI 90 |
|---|---|---|---|
| **Default model** | ,000 | ,000 | ,000 |
| **Saturated model** | ,000 | ,000 | ,000 |
| **Independence model** | 765,209 | 675,772 | 862,084 |

**FMIN**

| Model | FMIN | F0 | LO 90 | HI 90 |
|---|---|---|---|---|
| Default model | ,000 | ,000 | ,000 | ,000 |
| Saturated model | ,000 | ,000 | ,000 | ,000 |
| Independence model | ,700 | ,640 | ,565 | ,721 |

**RMSEA**

| Model | RMSEA | LO 90 | HI 90 | PCLOSE |
|---|---|---|---|---|
| Independence model | ,094 | ,089 | ,100 | ,000 |

**AIC**

| Model | AIC | BCC | BIC | CAIC |
|---|---|---|---|---|
| Default model | 176,000 | 178,735 | | |
| Saturated model | 176,000 | 178,735 | | |
| Independence model | 869,209 | 869,706 | | |

**ECVI**

| Model | ECVI | LO 90 | HI 90 | MECVI |
|---|---|---|---|---|
| Default model | ,147 | ,147 | ,147 | ,149 |
| Saturated model | ,147 | ,147 | ,147 | ,149 |
| Independence model | ,727 | ,652 | ,808 | ,727 |

**HOELTER**

| Model | HOELTER .05 | HOELTER .01 |
|---|---|---|
| Default model | | |
| Independence model | 134 | 148 |

**Execution time summary**

| | |
|---|---|
| **Minimization:** | ,033 |
| **Miscellaneous:** | ,505 |
| **Bootstrap:** | ,000 |
